# The distribution of fitness effects of nonsynonymous mutations varies phylogenetically across animals

**DOI:** 10.1371/journal.pbio.3003976

**Published:** 2026-09-08

**Authors:** Meixi Lin, Sneha Chakraborty, Carlos Eduardo G. Amorim, Sergio F. Nigenda-Morales, Annabel C. Beichman, Paulina G. Nuñez-Valencia, Jonathan C. Mah, Jacqueline A. Robinson, Christopher C. Kyriazis, Christian D. Huber, Andrew E. Webb, Sarah D. Kocher, Frederick I. Archer, Andrés Moreno-Estrada, Robert K. Wayne, Kirk E. Lohmueller

**Affiliations:** 1 Department of Ecology and Evolutionary Biology, University of California, Los Angeles, Los Angeles, California, United States of America; 2 Department of Integrative Biology, University of California, Berkeley, Berkeley, California, United States of America; 3 Center for Evolution and Medicine, School of Human Evolution and Social Change, Arizona State University, Tempe, Arizona, United States of America; 4 Advanced Genomics Unit, National Laboratory of Genomics for Biodiversity (Langebio), Center for Research and Advanced Studies (Cinvestav), Irapuato, Guanajuato, Mexico; 5 Department of Biological Sciences, California State University San Marcos, San Marcos, California, United States of America; 6 Department of Genome Sciences, University of Washington, Seattle, Washington, United States of America; 7 Centro de Ciencias Genómicas, Universidad Nacional Autónoma de México (UNAM), Cuernavaca, Morelos, Mexico; 8 Bioinformatics Interdepartmental Program, University of California, Los Angeles, Los Angeles, California, United States of America; 9 Department of Ecology and Evolutionary Biology, Princeton University, Princeton, New Jersey, United States of America; 10 Conservation Genetics, San Diego Zoo Wildlife Alliance, Escondido, California, United States of America; 11 Department of Biology, Pennsylvania State University, University Park, Pennsylvania, United States of America; 12 Howard Hughes Medical Institute, Chevy Chase, Maryland, United States of America; 13 Marine Mammal and Turtle Division, Southwest Fisheries Science Center, La Jolla, California, United States of America; University of Bath, UNITED KINGDOM OF GREAT BRITAIN AND NORTHERN IRELAND

## Abstract

The distribution of fitness effects (DFE) describes the selection coefficients of newly arising mutations and fundamentally influences population genetic processes. However, the extent and mechanisms of differences in the DFE for non-synonymous mutations have not been systematically investigated across species with divergent phylogenetic histories and ecologies. Here, we inferred the DFE in natural populations of 11 animal (sub)species, including humans, mice, fin whales, vaquitas, wolves, collared flycatchers, pied flycatchers, halictid bees, *Drosophila*, and mosquitoes. We found that mammals have a higher proportion of strongly deleterious mutations (defined as s≤−0.01; 22% to 47% in mammals; 0.0% to 5.4% in insects and birds) and a lower proportion of weakly deleterious mutations than insects and birds. Further, the DFE co-varies with phylogeny, such that the mean mutation effects are more similar in closely related species (Pagel’s λ = 0.84, *P* = 0.01). Next, we investigated whether various summary statistics of the DFE were related to variation in life-history traits across these organisms. We found some support for genome size, body mass, and long-term effective population size being correlated with the DFE. Overall, our findings are consistent with predictions derived independently from the Fisher’s Geometric Model (FGM), which defines organismal complexity as the number of phenotypes under selection. FGM predicts that mutations are more deleterious in complex organisms, while strongly deleterious mutations occur more frequently in smaller populations. Our study demonstrates strong phylogenetic signal in the evolution of a fundamental population genetics parameter, and proposes that, through mechanisms of epistasis, long-term population size and organismal complexity could be underlying variation in the DFE across animals.

## Abstract 2

The distribution of fitness effects (DFE) describes the impact of mutations on an organism’s ability to reproduce, but how and why does the DFE vary among species? This study analyzes genetic variation from 11 animal species, finding that fitness effects are influenced by phylogeny, population size and organismal complexity.

## Introduction

Mutations are the raw material on which other evolutionary forces act. The distribution of fitness effects (DFE) represents the relative proportion of new mutations with different selection coefficients (*s*) affecting the fitness of the individuals carrying them. In addition to its intrinsic value for understanding how mutations affect fitness, the DFE is a fundamental quantity in evolutionary genetics [1]. The DFE is essential for studying the maintenance of genetic variation, identifying causal variants underlying complex human disease, detecting selective sweeps while considering negative selection, and understanding the long-term survival of small populations [1–6]. Consequently, considerable effort has been put into characterizing the biological factors underlying DFE evolution, and estimating the DFE for a given species of interest [1,7]. However, the patterns of and mechanisms behind variation in the DFE have not been systematically surveyed across many species on a phylogenetic timescale [3].

Several theoretical models have proposed that organismal complexity and long-term effective population size ($N_{a}$) are pivotal biological factors influencing how the DFE might differ across species. While these two factors are common to many studies, predictions for how they affect the DFE vary across models [8–10]. While intuitive, a precise definition of complexity remains elusive. For example, complexity can refer to the number of unique cell types and protein-protein interactions [11,12]. Theoretically, complexity can refer to the number of genetically uncorrelated phenotypic traits under selection, denoting the dimensionality of phenotypic space in Fisher’s Geometric Model (FGM; [13,14]). Nevertheless, how complexity influences the DFE depends on the underlying model and associated assumptions. On the one hand, in complex organisms, highly connected networks confer robustness against the deleterious effects of mutations, therefore, more complex organisms are predicted to have fewer deleterious mutations [9,15]. Alternatively, assuming that the average effect of a mutation on each individual phenotype does not decrease with increasing complexity, the increased dimensionality of the phenotypic space results in larger effect sizes for random mutations, predicting a higher proportion of deleterious mutations in complex organisms [7,10,16].

Similarly, the effect of long-term population size on the DFE is multifaceted. In species with larger effective population sizes, more efficient natural selection favors stable proteins that are then less susceptible to disruption of function by subsequent mutations, thereby diminishing the average deleterious effects of new mutations [8,17]. In species with smaller effective population sizes, the reduced selective strength leads to the accumulation of deleterious mutations (termed “drift load"), moving the population from the fitness optimum. Consequently, a higher proportion of beneficial mutations is expected in species with smaller $N_{a}$ to compensate for this drift load [10]. Taken together, it has been hypothesized that the population-scaled selection coefficient ($2N_{a}s$) could be conserved across species, as the interplay between selective strength and protein stability may offset variation in long-term population sizes [8].

Despite the growing number of published DFEs in animal species, a lack of standardized DFE comparisons across lineages has hindered testing hypotheses regarding the evolutionary factors underlying potential variation in the DFE across species [3]. In natural populations, the DFE is usually estimated by contrasting allele frequencies, summarized by the site frequency spectrum (SFS), of neutrally evolving and selected variants in genomic data sets [18,19]. While DFE estimates for model organisms like humans [19,20], *Drosophila* [18,21], mice [7,22], and nematodes [23] have been available, the DFEs for non-model organisms, such as flycatchers [24], great apes [25], *Populus* [26], Hawaiian monk seals [27], and vaquitas [28], are emerging either species by species or within groups of closely related taxa. These DFE estimates agree that most amino-acid changing mutations are nearly neutral ($-10^{-5}<s\leq 0$) or weakly deleterious ($-0.001<s\leq -10^{-5}$), whereas beneficial (*s* > 0) and strongly deleterious (s≤−0.01) mutations are less common [1]. Additionally, the DFE appears to vary across divergent species [24,25,27,28]. For example, humans were found to carry a higher proportion of strongly deleterious mutations (s≤−0.01) than *Drosophila* [7,22]. Interestingly, closely related species demonstrated similar inferred DFE parameters [25,29]. Unfortunately, the scarcity of high-quality genomic data and genetic parameter estimates (e.g., mutation rates) in non-model organisms [30], coupled with methodological differences and computational inefficiencies in DFE inference [20,31,32], have prevented a more comprehensive comparison.

The recent increase in available genomic resources for non-model organisms now offers unprecedented opportunities for comparative population genomics [30]. Using such data, here we infer the DFE in 11 animal (sub)species from 10 species (*Canis lupus lupus* and *Canis lupus arctos* are subspecies of wolves) with diverse phylogenetic relationships and life history traits using *varDFE* (this study). We find signals of phylogenetic dependence in the evolution of DFE across species, with mutations being more deleterious in mammals compared with insects and birds. To evaluate the biological processes behind the variation of this fundamental distribution across species, we correlate proxies for organismal complexity and long-term population size with the DFE in different species. Overall, we find that mutations are more deleterious in more complex species and species with smaller long-term population sizes. These findings are consistent with Fisher’s Geometric Model and imply that epistasis could have played a role in DFE evolution.

## Results

### Population genetic inference for 11 animal (sub)species

**Fig 1:**
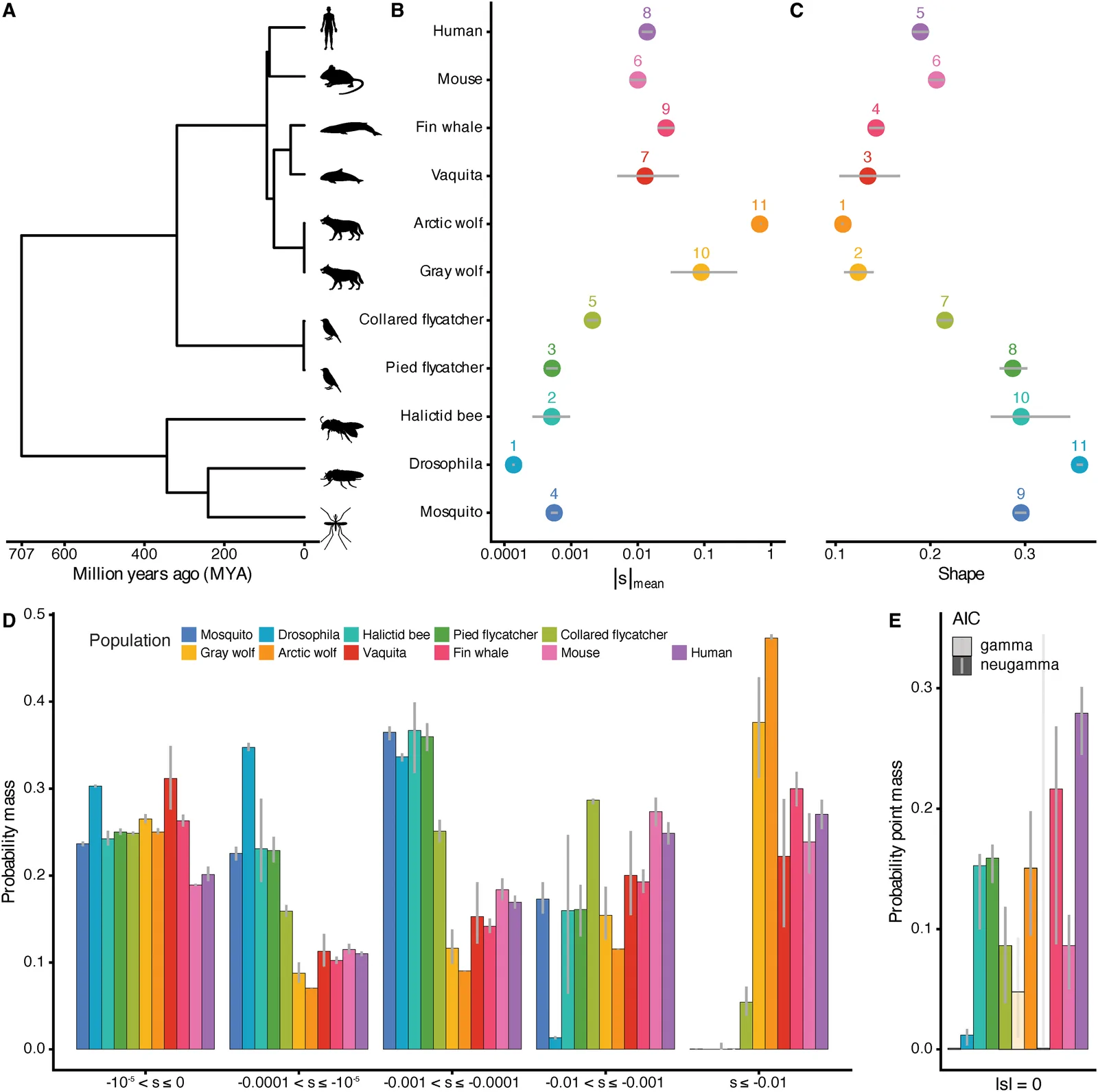
Mutations are more deleterious in mammals compared to insects and birds. **(A)** The phylogenetic relationship among the (sub)species included in this study. **(B)** The mean deleterious mutation effects (|*s*|_mean_) for 11 (sub)species, assuming a gamma-distributed DFE of neutral and deleterious mutations. From top to bottom, species are increasingly divergent from humans: mice, fin whales, vaquitas, arctic wolves, gray wolves, collared flycatchers, pied flycatchers, halictid bees, *Drosophila* and mosquitoes. Numbers show the ranks of |*s*|_mean_. **(C)** The shape parameter (α) of the gamma-distributed DFE for 11 (sub)species. Numbers show the ranks of α. **(D)** Proportions of mutations in various categories of |*s*|. From left to right, mutations range from (nearly) neutral ($-10^{-5}<s\leq 0$) to strongly deleterious (s≤−0.01). **(E)** The inferred probability point mass at *s* = 0 (*p*_neu_) assuming a gamma-distributed DFE with a neutral point mass (neugamma DFE). Bars are translucent for species where the Akaike Information Criterion (AIC) for the neugamma DFE is higher than that for the gamma DFE (which include mosquitoes, gray wolves and vaquitas). **(B–E)** Gray lines represent 95% confidence intervals derived from 200 Poisson-resampled SFS. The data and code needed to generate this Figure can be found in https://zenodo.org/records/21802302.

We retrieved high-quality population-level polymorphism datasets for 11 animal (sub)species. These data include three insect species (mosquitoes [33], *Drosophila* [7], and halictid bees [34]), two bird species (pied flycatchers and collared flycatchers [35]), and six mammal (sub)species from five species (arctic wolves [36,37], gray wolves [38], vaquitas [28], fin whales [39], mice [22], and humans [7]; arctic wolves and gray wolves are subspecies of wolves; Table 1, Text A in S1 Appendix). To further understand the mechanisms of DFE evolution, we also compiled several life history traits and genomic attributes for each species from literature, including mutation rate (Text B in S1 Appendix), long-term population size, body mass, generation time, age at maturity, maximum longevity, genome size and recombination rate (sources and citations in S1 Data). The 11 (sub)species we evaluated are distinctive in phylogenetic position (Fig 1A) and the compiled biological features that could affect the DFE. For example, the literature-derived long-term population sizes ($N_{a}^{\text{LIT}}$) varied from 4,485 (vaquitas) to $1.7\times 10^{6}$ (*Drosophila*) individuals. All datasets were filtered with the same stringency to retain only high-confidence genotype calls and at least eight diploid high-quality individuals in coding regions. We tallied the number of variants at different minor allele frequencies and generated folded site frequency spectra (SFS) for synonymous and nonsynonymous/missense variants (SYN-SFS and MIS-SFS, respectively) for each species (Fig A in S1 Appendix, S2 Data). To implement a robust but flexible workflow to compare the DFE across species, we developed *varDFE* (this study), a Python package based on the ∂a∂i and Fit∂a∂i packages (Fig B in S1 Appendix; [20,40]).

**Table 1:** Summary of DFE inference in 11 (sub)species’ datasets.

Common name	Scientific name	Class	Demography	SFS mask	$N_{a}^{SYN}$	|*s*|_median_	α	$\beta^{\prime }$
mosquito	*Anopheles coluzzii*	Insecta	three-epoch	TRUE	2,286,875	0.00013	0.30	0.0019
fruit fly	*Drosophila melanogaster*	Insecta	two-epoch	FALSE	2,766,461	$4.3\times 10^{-5}$	0.36	0.00038
halictid bee	*Lasioglossum calceatum*	Insecta	two-epoch	TRUE	280,467	0.00012	0.30	0.0017
pied flycatcher	*Ficedula hypoleuca*	Aves	two-epoch	FALSE	246,918	0.00012	0.29	0.0018
collared flycatcher	*Ficedula albicollis*	Aves	three-epoch	FALSE	138,027	0.00026	0.22	0.0097
gray wolf	*Canis lupus lupus*	Mammalia	three-epoch	FALSE	45,517	0.0017	0.12	0.72
arctic wolf	*Canis lupus arctos*	Mammalia	two-epoch	FALSE	74,139	0.0062	0.11	6.30
vaquita	*Phocoena sinus*	Mammalia	two-epoch	FALSE	6,264	0.00034	0.13	0.096
fin whale	*Balaenoptera physalus*	Mammalia	two-epoch	FALSE	12,411	0.00091	0.14	0.19
house mouse	*Mus musculus castaneus*	Mammalia	two-epoch	FALSE	207,682	0.0011	0.21	0.048
human	*Homo sapiens*	Mammalia	two-epoch	FALSE	7,041	0.0012	0.19	0.073

Summary of DFE inference in 11 (sub)species’ datasets, assuming a gamma-distributed DFE for new neutral or deleterious mutations (s≤0). “Demography” is the demographic model of choice used for long-term population size inference. “SFS mask” reports if singletons are masked in the site-frequency spectrum during the inference. “$N_{a}^{\text{SYN}}$” is the synonymous-site-based long-term population size in each dataset. “|*s*|_median_” is the estimated median mutation effects (s≤0). “α” and “$\beta^{\prime }$” are the inferred shape and scale parameters, respectively, for the gamma DFE.

We inferred demographic parameters from the SYN-SFS (Fig C in S1 Appendix, S3 Data). Although the demographic models we infer from synonymous variants could be biased by selection at linked sites, these demographic models nevertheless control for the distortion in the MIS-SFS caused by selection at linked sites and population history when inferring the DFE parameters [20]. Demographic models were selected using a stepwise likelihood-based procedure (Fig D and Text A in S1 Appendix). A demographic model with a single change in population size (i.e., a two-epoch model) provided a good fit to SYN-SFS and reasonable parameter estimates for most species except for the mosquitoes, halictid bees, gray wolves, and collared flycatchers. For the gray wolf and collared flycatcher datasets, the three-epoch demographic model (i.e., two changes in population size) performed better with more realistic parameter estimates or drastically improved fit to the SYN-SFS. For the mosquitoes and halictid bees datasets, no demographic model using the full SYN-SFS fit the data well, and using the singleton-masked SYN-SFS substantially improved model fit (S4 Data, Text A in S1 Appendix). All the inferred demographic models are qualitatively consistent with previous estimates (S5 Data). There is a strong correlation between literature-based estimates of $N_{a}^{\text{LIT}}$ and estimates of long-term population size from synonymous variants $N_{a}^{\text{SYN}}$ ($R_{\text{adj}}^{2}$ = 0.94, Fig E in S1 Appendix), suggesting that selection at linked sites has a limited effect on demographic inferences. We utilized the best-fit demographic parameters from the chosen models (Table 1) for downstream DFE inference.

### Mutations are more deleterious in mammals compared with insects and birds

Conditional on the inferred demography, we estimated the DFE for new nonsynonymous mutations for each species, assuming that the DFE follows a gamma distribution [20], mutations are neutral or deleterious (s≤0), and mutations act in an additive manner (*h* = 0.5). The gamma distribution yielded a robust fit to the observed MIS-SFS across all datasets (Fig F in S1 Appendix, S6 Data).

Overall, mutations are 4.8 to 4,923 times more deleterious in mammals (the mean selection coefficient, |*s*|_mean_, is 0.010 in mice to 0.67 in arctic wolves) compared to insects (|*s*|_mean_ = 0.00014 in *Drosophila* to 0.00056 in mosquitoes) and birds (|*s*|_mean_ = 0.00052 in pied flycatchers and |*s*|_mean_ = 0.0021 in collared flycatchers; Fig 1B). Noticeably, the scale ($\beta^{\prime }$) parameter for the arctic wolves reached the upper boundary of the parameter space during inference. To rule out potential data artifacts, we analyzed an independently generated, whole-genome dataset from Russian Karelia gray wolves [41], which also yielded a high $\beta^{\prime }$ value, confirming |*s*|_mean_ is very deleterious in wolves. Since |*s*|_mean_ may be heavily influenced by the strongly deleterious tail of the DFE, which we have less ability to accurately estimate, we also examined the median |*s*| (|*s*|_median_) values across species. Consistent with patterns in |*s*|_mean_, median selective effects are more deleterious in mammals (|*s*|_median_ = 0.00034 in vaquitas to 0.0062 in arctic wolves) compared to insects and birds (|*s*|_median_ = $4.3\times 10^{-5}$ in *Drosophila* to 0.00026 in collared flycatchers; Fig G in S1 Appendix). The shape (α) parameter of the gamma distribution differs between species groups as well. On average, mammals have lower estimates for the shape parameter (α = 0.11 in arctic wolves to α = 0.21 in mice) compared to insects and birds (α = 0.22 in collared flycatchers to α = 0.36 in *Drosophila*; Fig 1C). The extent of variation in α that we detected is in line with previous findings [29].

We also observed that mammals have higher proportions of strongly deleterious mutations (s≤−0.01) than insects and birds, consistent with these species having more deleterious |*s*|_mean_ estimates (Fig 1D). The maximum-likelihood gamma distribution for each species revealed that 22% (vaquitas) to 47% (arctic wolves) of mutations in mammals are strongly deleterious (s≤−0.01), compared to 0.0% (*Drosophila*) to 5.4% (collared flycatchers) in insects and birds. On the other hand, insects and birds have a larger proportion of weakly deleterious mutations ($-0.001<s\leq -10^{-5}$, 41% in collared flycatchers to 68% in *Drosophila*), compared to mammals (16% in arctic wolves to 30% in mice). The proportions of neutral to nearly neutral mutations ($-10^{-5}<s\leq 0$) are similar in all 11 (sub)species. To directly estimate the proportion of (nearly) neutral mutations, we considered a different functional form, where DFE followed a mixture of gamma distribution with an additional point mass at neutrality (neugamma DFE [7], eq 1, S7 Data). This neugamma DFE improved model fit for most datasets (lower AIC scores) compared to the gamma DFE (Figs H and I in S1 Appendix), except for mosquitoes, gray wolves, and vaquitas. Similar to the patterns obtained assuming a gamma DFE (Fig 1D), the inferred proportion of neutral mutations (*p*_neu_) varied across lineages (Figs 1E, Fig J in S1 Appendix). *p*_neu_ varied from 0.081% (mosquitoes) to 16% (pied flycatchers) in insects and birds, and from 0.089% (vaquitas) to 28% (humans) in mammals. We also attempted to directly estimate the proportion of lethal mutations (s=−0.5) [20,42]. However, our approach likely lacks sufficient power to confidently infer the proportion of lethal mutations, due to poor model fit and unrealistic estimates of the proportion of lethal mutations (Figs I–K and Text C in S1 Appendix, S8 Data).

To evaluate the robustness of our analysis, we tested whether several technical treatments affected the DFE inference. First, we examined different probability distributions for the DFE, including the gamma, neugamma, and lognormal distributions (Figs L and M in S1 Appendix, S9 Data), and compared the median selective effects (|*s*|_median_) across distributions. When assuming a lognormal DFE, we focused on comparing median (|*s*|_median_) rather than mean selective effects (|*s*|_mean_), as the inferred distribution includes a long tail of strongly deleterious mutations. In this case, the median provides a more robust and interpretable summary. When assuming the neugamma DFE, |*s*|_median_ remained more deleterious in mammals compared with insects and birds (Fig G in S1 Appendix). When assuming the lognormal DFE, this pattern was largely maintained, although the |*s*|_median_ estimate in vaquitas (|*s*|_median_ = 0.00029) became lower than those in mosquitoes (0.00034) and collared flycatchers (0.00037; Fig G in S1 Appendix). However, the AIC for the lognormal DFE is the lowest only in four datasets (mosquitoes, gray wolves, arctic wolves, and vaquitas; Fig N in S1 Appendix). Furthermore, the lognormal distribution exhibits a long tail of strongly deleterious mutations (Figs L and O in S1 Appendix), where there is the least confidence of ∂a∂i-based DFE inference [42]. Both of these factors suggest that a lognormal-distributed DFE is less supported by the data. The gamma distribution was confirmed as an adequate functional form for DFE comparisons, given its parsimony, and its second-lowest AIC across most datasets (Fig N in S1 Appendix), and good visual fit to the observed SFS (Fig F in S1 Appendix). Next, we tested the potential impacts of demographic model misspecification, singleton-masking treatments, mutation rate variation, differences in nonsynonymous to synonymous mutation rate ratio ($r_{L}$), and GC-biased gene conversion, on the inference of the DFE. The average mutation effects and proportion of each mutation category remain unchanged qualitatively across these different technical treatments (Figs P-U and Text D in S1 Appendix, S10 Data - S14 Data).

To confirm our DFE inference can confidently detect strongly deleterious mutations (s≤−0.01) even in very large populations, we performed a SLiM simulation using demographic parameters inferred from *Drosophila* and DFE parameters inferred from humans. The long-term population sizes ($N_{a}$) inferred from synonymous and non-coding mutations in the simulation are both lower than the true *Drosophila* population size used, as expected due to selection at linked sites (Fig V in S1 Appendix). Using a sample size of 100, $N_{a}$ was underestimated by 3.8%–5.0% when using the SFS from synonymous variants and by 2.8%–3.5% when using the SFS from non-coding variants. With a sample size of 10, underestimation was 3.1%–5.2% and 1.7%–2.5%, respectively. The DFE inferred from the simulation closely matches the true human DFE used in the simulation, where the assumed human |*s*|_mean_ is 0.014 (Table 1). The average inferred |*s*|_mean_ is 0.014 (95% CI: 0.012–0.017) when using an SFS sample size of 100, and 0.015 (95% CI: 0.010–0.023) with a sample size of 10 (Fig W in S1 Appendix). This simulation indicates that our methods can confidently detect strongly deleterious mutations (s≤−0.01) and correctly infer |*s*|_mean_ in large populations, even when estimates of long-term population size from synonymous variants are biased due to selection at linked sites. To assess whether accurate inference is robust to misspecification of the assumed functional form of the DFE, we also fit a lognormal distribution to the simulated data, and compared its median to the true human DFE used to generate the data. The average inferred |*s*|_median_ assuming a lognormal-distributed DFE is 0.014 (95% CI: 0.012–0.017) when using an SFS sample size of 100, and 0.015 (95% CI: 0.010–0.023) with a sample size of 10. These estimates are larger than the true gamma-distributed human |*s*|_median_ of 0.0012. However, the direction of the bias is away from the more neutral *Drosophila*-like DFE, suggesting that estimates of |*s*|_median_ are not under-estimated in extremely large populations (Fig X in S1 Appendix). Thus, the lack of strongly deleterious mutations inferred in species with large population sizes is likely not due to biases in our inference procedure, and instead reflects true differences in the DFE.

In summary, the distribution of fitness effects is more similar in closely related species, with mammals harboring more strongly deleterious and less weakly deleterious variations compared with insects and birds.

### The DFE co-varies with phylogeny in animals

The apparent co-variation of the DFE with phylogeny motivated us to test for a phylogenetic signal more formally. Pagel’s λ quantifies how trait evolution depends on the underlying phylogenetic tree across species, where λ = 0 suggests no phylogenetic signal and λ = 1 indicates strong phylogenetic dependence [43]. Assuming a gamma-distributed DFE, we calculated Pagel’s λ for log10 transformed mean selection coefficients [$\log_{10}$(|*s*|_mean_)], log10 transformed median selection coefficients [$\log_{10}$(|*s*|_median_)], and the shape parameter (α, Fig 1B – 1C). For $\log_{10}$(|*s*|_mean_), Pagel’s λ was 0.84 (*P* = 0.01, likelihood ratio test [LRT]). A strong phylogenetic signal was also observed for $\log_{10}$(|*s*|_median_) (λ = 0.76, *P* = 0.03) and the shape parameter (λ = 0.86, *P* = 0.004). Since the DFE parameters for arctic wolves reached upper boundaries, Pagel’s λ was recalculated excluding the arctic wolves dataset. Strong phylogenetic signal was consistently observed for all metrics (λ = 0.86, *P* = 0.008 for $\log_{10}$(|*s*|_mean_); λ = 0.81, *P* = 0.02 for $\log_{10}$(|*s*|_median_); λ = 0.81, *P* = 0.01 for α). To account for the long phylogenetic branches between mammals and insects, we repeated Pagel’s λ analysis only within mammals. Signals of phylogenetic dependence were reduced (λ = 0.45, *P* = 0.49 for $\log_{10}$(|*s*|_mean_); λ = 0.15, *P* = 0.88 for $\log_{10}$(|*s*|_median_); λ = 0.90, *P* = 0.08 for α), which could either be due to less of a signal within mammals or reduced statistical power due to the small number of species analyzed. Overall, these analyses suggest that the evolution of DFE is influenced by the shared evolutionary history determined by the phylogenetic structure, but phylogenetic dependence is less evident within mammals (S15 Data).

**Fig 2:**
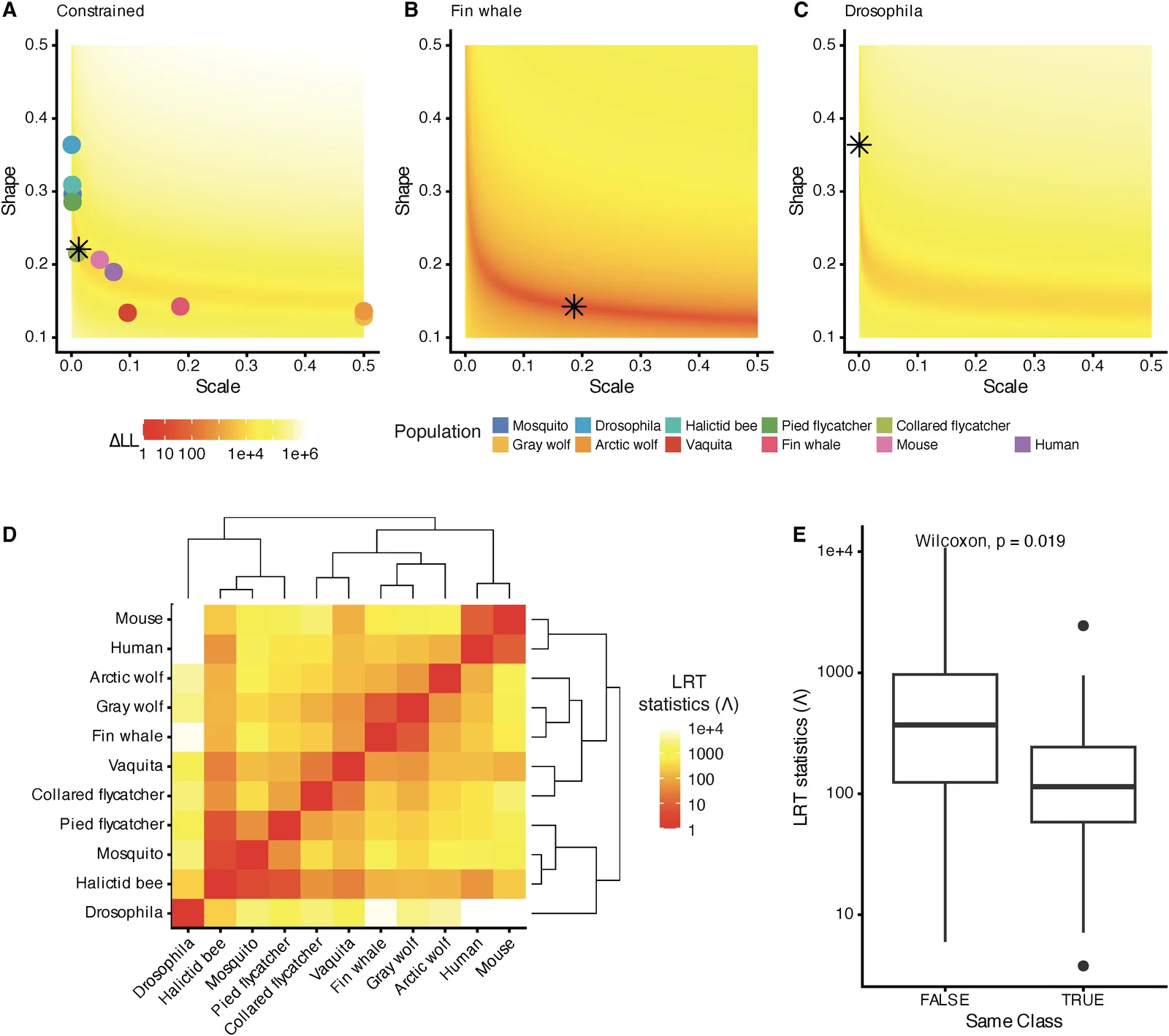
Grid-search-based likelihood-ratio tests confirm the phylogenetic signal in DFE evolution. **(A–C)** The log-likelihood surfaces for the shape (α) and scale ($\beta^{\prime }$) parameters (**A**) under the null model where all species are constrained to the same parameters or under the alternative model allowing species’ parameters to vary. Representative log-likelihood surfaces are shown for (**B**) fin whales and **(C)** *Drosophila* datasets (other species’ log-likelihood surfaces shown in Fig Y in S1 Appendix). Background colors from yellow to red indicate the differences in log-likelihood for given parameters to data. On each log-likelihood surface, the maximum likelihood estimate (MLE) derived for (**A**) the null model or (**B–C**) the alternative model in respective species is overlaid as the asterisk. In **(A)**, the colored points showing MLEs for each species derived from the alternative model are overlaid for comparison. The two wolves datasets’ MLEs derived from parameter optimization procedures (Fig 1) exceed the grid-search derived MLE plotted here. For all other species, the MLEs from the grid search and parameter optimization are the same. **(D)** Pairwise LRT statistics (Λ) for each species pair are colored on a log10 scale and hierarchically clustered. Darker cells represent more similar DFE estimates in the species compared, such as the human-mouse pair, whereas lighter cells represent more distinct DFEs, such as the human-*Drosophila* pair. The dendrogram derived from hierarchical clustering is annotated. **(E)** The boxplot of pairwise LRT statistics in **(D)**, categorized by whether the species pair belongs to the same Class. The y-axis is on a log10 scale. The data and code needed to generate this Figure can be found in https://zenodo.org/records/21802302.

To formally test whether the parameters of a gamma-distributed DFE vary across species, we examined a null model where the shape and scale parameters of the gamma distribution were constrained to be the same across the 11 (sub)species datasets, while still accounting for their individual demographic histories. Using a grid-search approach, we calculated the Poisson likelihood of the expected MIS-SFS given each parameter combination in each species. The maximum likelihood estimate (MLE) for this null model is found by summing the individual likelihood surfaces of each species’ DFE (Fig 2A). The alternative model allows each species to have its own shape and scale parameters, as described in previous sections (Fig 2B – 2C). This alternative model fits the data (MIS-SFS) significantly better than the null model (LRT, Λ = 18,203, df = 20, *P* < 10^−16^; Figs 2A – 2C, Fig Y in S1 Appendix). Therefore, the variation of the DFE across taxa is statistically supported by our analyses.

To further investigate the relationship between phylogenetic proximity and DFE similarity, we next used the likelihood ratio test (LRT) framework to compare pairs of species. Here, the null model is relaxed to constrain DFE parameters to be identical only for the two species compared, rather than across all species. The resulting LRT statistic (Λ) serves as a measure of DFE similarity between species pairs, with lower values indicating higher similarity (eq 2). To visualize pairwise comparisons, we conducted hierarchical clustering of log10-scaled pairwise LRT statistics [$\log_{10}(\Lambda )$]. The insects and mammals clustered into two main lineages, with birds being split between the two: the pied flycatchers fell into the insect lineage while the collared flycatchers clustered with vaquitas (Fig 2D). In addition, $\log_{10}(\Lambda )$ is significantly lower (*P* = 0.02, Wilcoxon test) in pairwise comparisons within the same taxonomic class (average $\log_{10}(\Lambda )$ = 2.06±0.71), such as mammal-mammal pairs, compared to inter-class pairwise comparisons (average $\log_{10}(\Lambda )$ = 2.59±0.76), such as mammal-insect pairs (Fig 2E). In summary, species that are more closely related phylogenetically, tend to have more similar DFEs compared to species that are more distantly related to each other.

### The relationship between life history traits and the DFE

**Fig 3:**
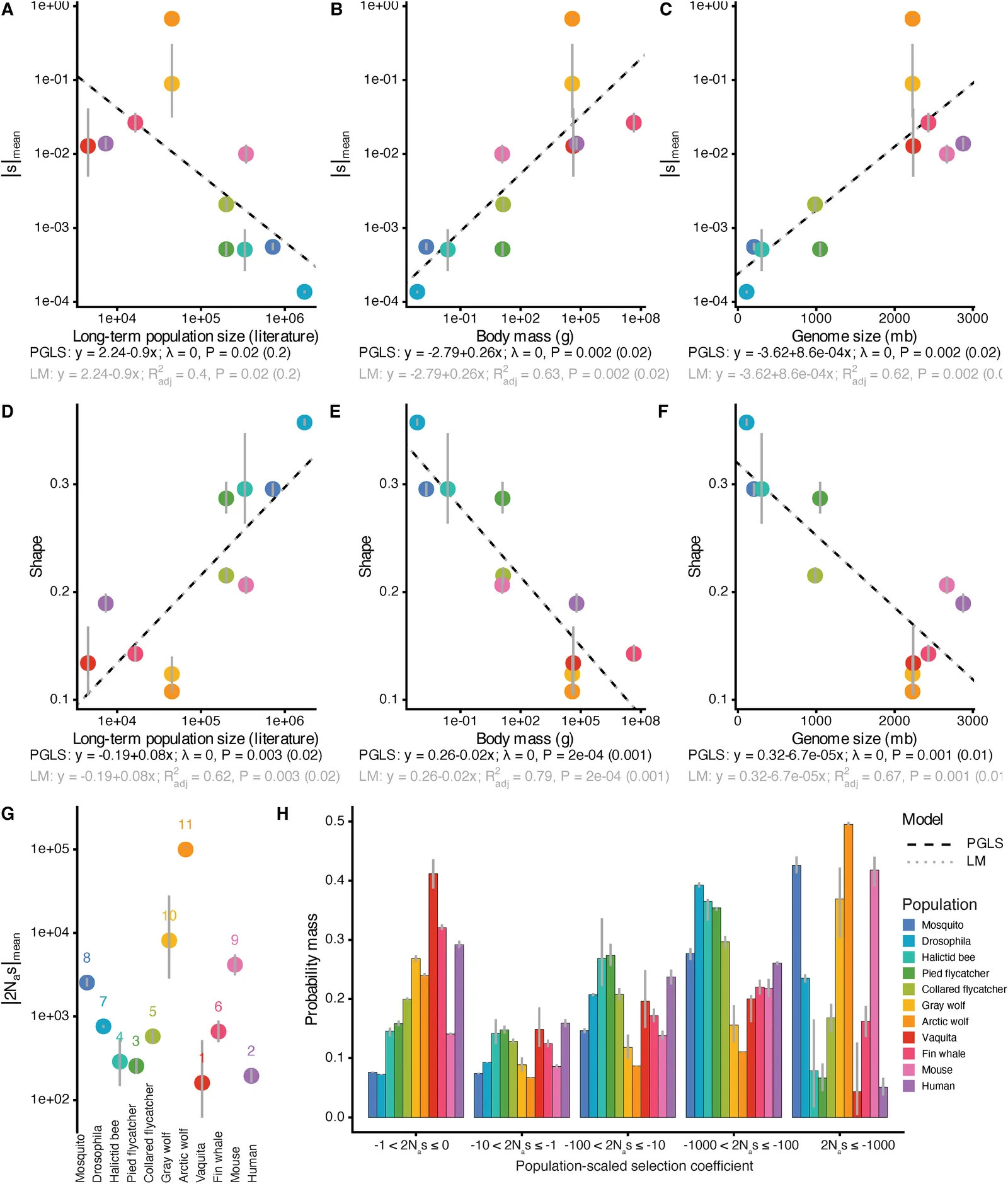
Testing for associations between life-history traits and the DFE across species. The phylogenetic generalized least squares model with a simultaneously inferred phylogenetic signal (${\text{PGLS}}_{\lambda }$) was fitted for |*s*|_mean_ with **(A)** long-term population size, **(B)** body mass, and **(C)** genome size. **(D–F)** The relationship between the shape parameter (α) of the gamma DFE and the same life-history traits shown above. The ${\text{PGLS}}_{\lambda }$ results are overlaid as a dashed black line, with equations, inferred phylogenetic signal (λ), and likelihood-ratio derived *p*-value annotated at the bottom. The linear regression results are overlaid as a dotted gray line, with equations and *p*-values annotated at the bottom. *P*-values in parentheses include a Bonferroni correction for nine life-history traits. **(G)** Assuming a gamma-distributed DFE, the mean population-scaled deleterious mutation effects ($|2N_{a}s{|}_{\text{mean}}$) are not equal across 11 species. Numbers show the ranks of $|2N_{a}s{|}_{\text{mean}}$. **(A–G)** Each point represents one species, with gray vertical lines representing confidence intervals. All axes are on a log10 scale, except for the genome size and the shape parameter. **(H)** Proportions of mutations with various ranges of $\left|2N_{a}s\right|$. From left to right, mutations ranged from (nearly) neutral ($-1<2N_{a}s\leq 0$) to strongly deleterious ($2N_{a}s\leq -1000$). Gray lines represent 95% confidence intervals. The data and code needed to generate this Figure can be found in https://zenodo.org/records/21802302.

We next explored the relationship between the DFE and candidate life history traits related to long-term population size and organismal complexity, using the phylogenetic generalized least squares model (${\text{PGLS}}_{\lambda }$ [44]). ${\text{PGLS}}_{\lambda }$ estimates the amount of phylogenetic signal (λ) in the regression residuals and incorporates λ in regression parameter estimates to control for interspecific autocorrelation due to phylogeny. Using this framework, we examined the relationship between the gamma-distributed DFE parameters (|*s*|_mean_, |*s*|_median_ and the shape parameter) and nine different life history traits. First, we explored the relationship between the DFE and long-term effective population size. While it would seem natural to test for a correlation between |*s*|_mean_ and $N_{a}^{\text{SYN}}$, $N_{a}^{\text{SYN}}$ was used to convert $2N_{a}s$ to *s*, which could induce a negative correlation between |*s*|_mean_ and $N_{a}^{\text{SYN}}$ (Text E in S1 Appendix). To partially mitigate this effect, we instead used published independently-derived estimates of long-term population size made from noncoding variants (Fig E in S1 Appendix). We observed a marginally significant negative correlation (Fig 3A) between the literature-based long-term population sizes and log10 transformed mean selection effects $\log_{10}(|s{|}_{\text{mean}})\sim \log_{10}(N_{a}^{\text{LIT}})$, (${\text{PGLS}}_{\lambda }$, slope (*b*) = −0.90, λ = 0, *P* = 0.02), though this correlation was no longer significant after Bonferroni correction for nine traits (*P*_adj_ = 0.2). On the other hand, statistically stronger positive correlations were observed for body mass and genome size with |*s*|_mean_ (body mass: ${\text{PGLS}}_{\lambda }$, *b* = 0.26, λ = 0, *P* = 0.002 [0.02]; genome size: ${\text{PGLS}}_{\lambda }$, *b* = $8.6\times 10^{-4}$, λ = 0, *P* = 0.002 [0.02]; Fig 3B – 3C). Other parameters tested, including the generation time, age at maturity, maximum longevity, recombination rates, and mutation rates, were not significantly correlated with |*s*|_mean_ after considering phylogenetic dependence (Fig Z in S1 Appendix). The two wolf (sub)species’ DFE deviated the most from the regression, likely due to their very deleterious estimated DFEs.

As |*s*|_mean_ is sensitive to the value of $N_{a}$ to unscale the scale parameter in the gamma distribution, we next focused on the shape (α) parameter of the gamma distribution. The shape parameter is not influenced by $N_{a}^{\text{SYN}}$ (Text E in S1 Appendix), and often estimated more confidently than the scale parameter in DFE inference [20,29]. We performed the ${\text{PGLS}}_{\lambda }$ analysis between α and life history traits (Fig AA in S1 Appendix). Literature-based long-term population size was positively correlated with α (*P* = 0.003 [0.02 after bonferroni-adjustment], Fig 3D), whereas body mass and genome size were negatively correlated with α (*P* = $2\times 10^{-4}$ [0.001], *P* = 0.001 [0.01], respectively; Fig 3E – 3F). Although not significantly correlated with |*s*|_mean_, age at maturity is negatively correlated with α (*P* = 0.004 [0.04], Fig AA in S1 Appendix). We performed the ${\text{PGLS}}_{\lambda }$ analysis between $\log_{10}(|s{|}_{\text{median}})$ and life history traits as well (Fig AB in S1 Appendix). The correlation with $N_{a}$ is less pronounced, with synonymous-site-based, but not literature-based, long-term population size weakly correlating with $\log_{10}(|s{|}_{\text{median}})$ (*P* = 0.04 [0.4]). Body mass and genome size remain correlated with $\log_{10}(|s{|}_{\text{median}})$ (*P* = 0.006 [0.06], *P* = 0.001 [0.01], respectively). We also repeated the ${\text{PGLS}}_{\lambda }$ analyses excluding the arctic wolves outlier. Literature-based long-term population size, body mass, and genome size remain correlated at various degrees with |*s*|_mean_, |*s*|_median_ and α (S16 Data). To account for the long phylogenetic branches between mammals and insects, we repeated the ${\text{PGLS}}_{\lambda }$ analyses only within mammals. None of the traits tested were significantly correlated with |*s*|_mean_, |*s*|_median_ or α after Bonferroni-adjustment. Only a marginally significant correlation between the shape parameter and genome size was observed (*P* = 0.01 [0.1]; Figs AC–AE in S1 Appendix).

The marginally significant negative correlation of population size and mean mutation effects (|*s*|_mean_) suggests potential constancy of population-scaled mutation effects across species ($\gamma =2N_{a}s$) as previously hypothesized [8]. Therefore, we next sought to determine if population size by itself can explain the variation in the DFE observed across species by testing whether the population-scaled DFE varies across species. Assuming the population-scaled DFE follows a gamma distribution, the mean mutation effects ($|2N_{a}s{|}_{\text{mean}}$) still varied across species (Fig 3G–3H). While |*s*|_mean_ co-varied with phylogeny, this same phylogenetic signal was not observed in estimates of $|2N_{a}s{|}_{\text{mean}}$ (λ = 0, *P* = 1 for $\log_{10}$($|2N_{a}s{|}_{\text{mean}}$)). The mean population-scaled mutation effects are the highest in two wolves populations ($|2N_{a}s{|}_{\text{mean}}$ = $1.0\times 10^{5}$ in arctic wolves and 8,120 in gray wolves), and the lowest in the vaquitas (161) and humans (195; Fig 3G). The proportion of mutations with different population-scaled selection coefficients varied substantially within Classes as well (Fig 3H). For example, humans were estimated to have only 5.12% mutations with $2N_{a}s<-1000$, while mice have 41.8% of mutations in this category. Adopting the grid-search analysis for population-scaled DFE comparisons, we found that the alternative model where each species has its own population-scaled shape and scale parameters fit the data significantly better than the null model assuming a constant γ in different species (LRT, Λ = 99,508, df = 20, *P* < 10^−16^; Fig AF in S1 Appendix). When comparing the population-scaled DFE between species pairs, $\log_{10}(\Lambda )$ for γ remained significantly lower (*P* = 0.01, Wilcoxon test) in pairwise comparisons within the same taxonomic class ($\log_{10}(\Lambda )$ = 2.98±0.62) compared to inter-class comparisons (3.47±0.75; Fig AG in S1 Appendix), as observed in the $\log_{10}(\Lambda )$ statistics for *s* (Fig 2E). However, we repeated the ${\text{PGLS}}_{\lambda }$ analyses for $|2N_{a}s{|}_{\text{mean}}$, and none of the nine traits tested was significantly correlated with $|2N_{a}s{|}_{\text{mean}}$ (Fig AH in S1 Appendix).

### Fisher’s geometric model enables concurrent examination of the influence of population size and organismal complexity on the DFE

Previous work has suggested that Fisher’s geometric model (FGM) can explain patterns of DFE variation across divergent species. FGM models the fitness landscape with a minimal set of parameters to study the emergent properties of mutational effects [14]. In FGM, the fitness of an organism is characterized by a multi-dimensional Gaussian decay function with a local optimum in *n*-dimension phenotype space, where the phenotype dimensionality *n* is termed “organismal complexity". Mutations create new phenotypes, modeled as random vectors with effect scale σ that act within a subset of *m* phenotype dimensions (m≤n, *m* is defined as mutation pleiotropy) [10]. Both *m* and σ reflect intrinsic features of an organism’s genotype–phenotype-fitness architecture, and serve as proxies for organismal complexity from different perspectives [7,16]. When the population is at the fitness optimum and mutations are universally pleiotropic (*m* = *n*), the derived DFE is a gamma distribution with only neutral or deleterious mutations (s≤0) [16]. When the population is under mutation-selection-drift equilibrium, the increase in fitness from beneficial mutations should counteract the drift load, i.e., the decrease in fitness caused by fixed deleterious mutations, and the population has an equilibrium phenotypic distance ($z_{\text{eq}};z_{\text{eq}}>0$) to the fitness optimum. The formula for this FGM-derived DFE, which models the full DFE (−0.5≤s≤0.5), is a function of three key parameters in FGM (eq 3): long-term population size ($N_{a}^{\text{FGM}}$), mutation pleiotropy (*m*), and scale of mutation effects (σ).

**Fig 4:**
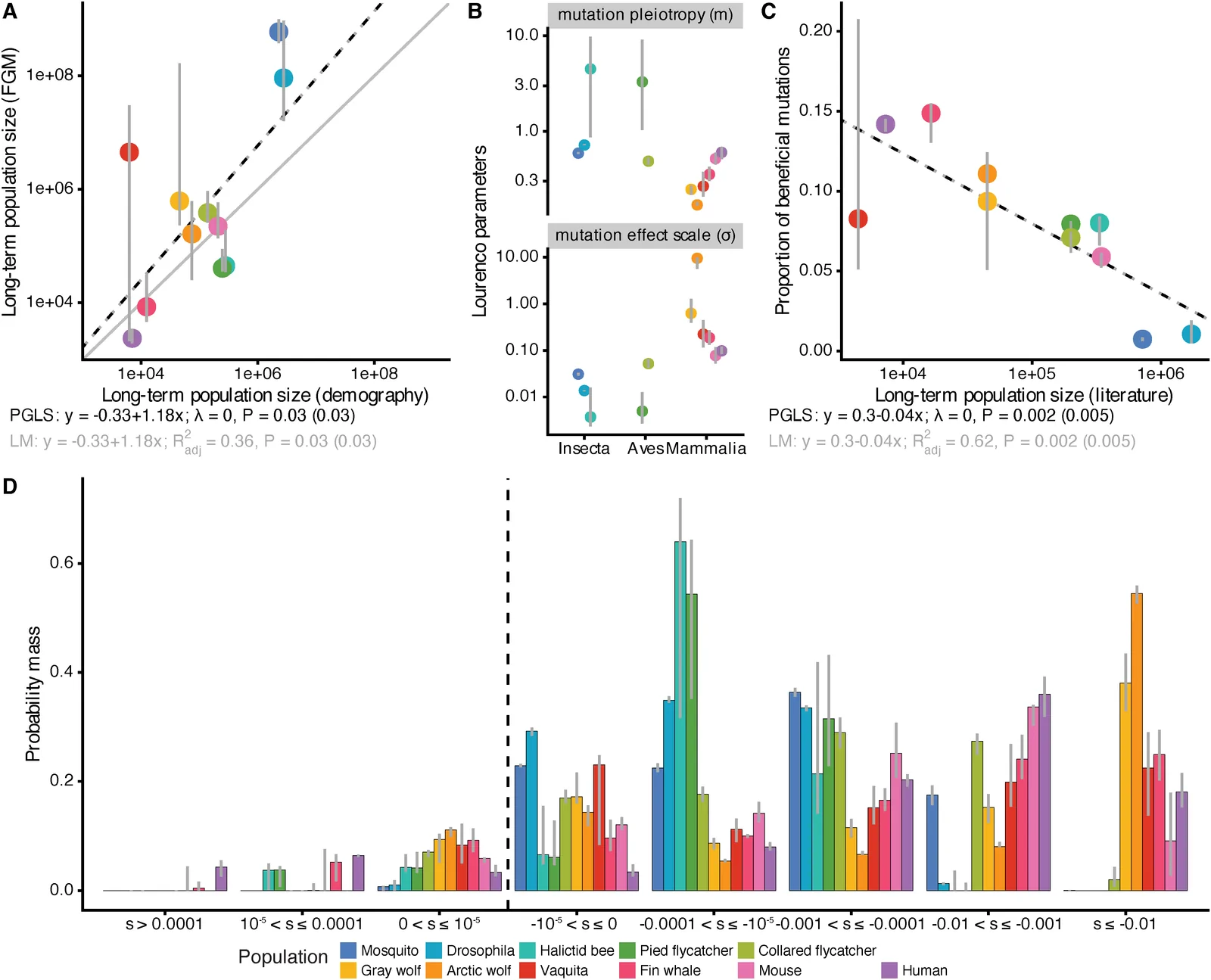
Fitting a Fisher’s Geometric Model (FGM)-derived DFE. **(A)** The inferred long-term effective population size ($N_{a}^{FGM}$) from the FGM-derived DFE (y-axis) is correlated with the effective size estimates from our demographic inference (x-axis). Axes are on a log10 scale. **(B)** The estimated scale of mutation effects (σ) and mutation pleiotropy (*m*) parameters for 11 (sub)species, grouped by the Class of each species. Note that σ is higher in mammals compared to insects. The y-axis is on a log10 scale. **(C)** The estimated proportion of beneficial mutations (*s* > 0) is negatively correlated with estimates of long-term population size from the literature. The x-axis is on a log10 scale. **(D)** Proportions of mutations in various categories of *s* estimated from the FGM-derived DFE for each species. From left to right, mutations range from beneficial (*s* > 0), (nearly) neutral ($-10^{-5}<s\leq 0$) to strongly deleterious (s≤−0.01). Dashed line marks *s* = 0. Gray lines represent 95% confidence intervals. The data and code needed to generate this Figure can be found in https://zenodo.org/records/21802302.

We inferred the parameters for this FGM-derived DFE for 11 animal (sub)species (eq 3, derived in [10]). The FGM-derived DFE provided an equally good or better fit to the MIS-SFS compared to the gamma DFE (Figs AI and AJ in S1 Appendix, S17 Data). In addition, the inferred long-term population sizes from the FGM-derived DFE ($N_{a}^{\text{FGM}}$) largely agree with the estimates of $N_{a}^{\text{SYN}}$ derived from demographic inference using genetic variation data ($R_{\text{adj}}^{2}$ = 0.36, linear regression; *P* = 0.03, ${\text{PGLS}}_{\lambda }$; Fig 4A), providing additional validation of this model. However, $N_{a}^{\text{FGM}}$ estimates are often higher than $N_{a}^{\text{SYN}}$, which could be due to the different biological interpretations in these two $N_{a}$ estimates. $N_{a}^{\text{SYN}}$ is inferred from demographic modeling using putatively neutral polymorphisms, whereas $N_{a}^{\text{FGM}}$ is inferred from deleterious variation at nonsynonymous sites. $N_{a}^{\text{FGM}}$ reflects the equilibrium distance from the fitness optimum, and likely captures a much longer time period than the neutral coalescent time that $N_{a}^{\text{SYN}}$ represents [45]. The vaquita population’s $N_{a}$ estimate deviated the most between the two methods, likely reflective of its long-term small population size and recent bottleneck [28].

We found some support that the mutation effect scale (σ) is reflective of a species’ phylogenetic position. Mammals have larger σ (0.077 to 9.5) compared with insects and birds (0.0037 to 0.052; Fig 4B). The mutation pleiotropy (*m*) is negatively correlated with the inferred σ ($R_{\text{adj}}^{2}$ = 0.76, linear regression, *P* < 0.001, ${\text{PGLS}}_{\lambda }$; Fig AK in S1 Appendix), suggesting potential auto-correlations during inference. Although less conserved within lineages, mammals tend to have smaller *m* (0.17 to 0.60) compared with insects and birds (0.49 to 4.5). However, Pagel’s λ did not suggest phylogenetic signal for either σ (λ = 0, *P* = 1) or *m* (λ = 0.51, *P* = 0.67; S15 Data).

Next we examined the proportion of mutations with various ranges of *s* predicted from the FGM-derived DFE (Fig 4D). Similar to the findings assuming the gamma DFE, strongly deleterious mutations are scarce in insect and bird populations (0.0% to 2.0% in collared flycatchers), but much more abundant in mammals (9.1% in mice to 55% in arctic wolves). Additionally, we found that the proportion of beneficial mutations varied among populations. FGM predicts that as the population size decreases, the proportion of beneficial mutations increases to counteract the increased drift load [10]. Indeed, assuming FGM, we found that the proportion of beneficial mutations is negatively correlated with the long-term effective population size taken from the literature ($R_{\text{adj}}^{2}$ = 0.73, linear regression; *P* < 0.001, ${\text{PGLS}}_{\lambda }$; Fig 4C), providing further support for this model. Note that here we are relating the proportion of beneficial mutations to the effective population sizes estimated from the literature, and as such, this correlation is not directly induced by the FGM being fit to the data.

## Discussion

Here, we evaluated the long-standing question of the evolutionary stability of the DFE across species, taking advantage of the advances in whole genome sequencing of non-model organisms. To do this, we curated high-quality whole-genome-based polymorphism data with a large sample size per species. Our results suggest that evolution of the DFE is contingent upon the underlying phylogenetic relationships across species, with closely-related species sharing a more similar DFE (Fig 2). Overall, mutations are more deleterious in mammals, compared to insects and birds (Figs 1, 4). We also observe potential correlations between the DFE with long-term population size and organismal complexity that warrant future investigation (Figs 3, 4).

The DFE estimates we obtained align with those of previous studies (S18 Data). In humans, the inferred gamma distribution shape parameter in our study (0.19 ± 0.0048), falls within the range of previous studies: from 0.12 ~ 0.16 [29], 0.16 [25], 0.18 ~ 0.21 [19], 0.17 ~ 0.21 [20], to 0.2 [18]. In *Drosophila*, our inferred shape parameter of 0.36 ± 0.0017 aligns with previous estimates: from 0.35 [18], to 0.32 ~ 0.41 [29]. Even in non-model organisms, our DFE inferences are concordant with published studies [22,24,28,29]. The consistency of our DFE estimates with the literature further confirms that the DFE variation observed across species is likely of biological relevance. Notably, we found an unusually high proportion of deleterious mutations in the DFE in two wolf species (Fig 1B). As we currently lack a satisfying explanation for the distinct DFE in canids, it is worthy of further investigation [46]. Additionally, our results are broadly consistent with the selective constraint inferred from nonsynonymous to synonymous divergence across species [22]. The ratio of nonsynonymous to synonymous substitution (*dN*/*dS*) is influenced by $2N_{a}s$ [47]. Therefore, most of the nonsynonymous divergence that accumulates across species is nearly neutral at the population level ($-1<2N_{a}s<0$). Here we observed the highest proportion of mutations with $-1<2N_{a}s<0$ in humans, followed by mice and then *Drosophila* (Fig 3H), matching previous observations that *dN*/*dS* is highest in humans, intermediate in mice, and lowest in flies [22].

Our study has a number of important limitations. Similar to prior research, the DFEs inferred in this study are restricted to nonsynonymous mutations, and we assume that such mutations have additive effects within and across loci. The relationship between dominance effects and selection coefficients is important, yet hard to characterize [48,49]. When a single dominance coefficient is assumed for nonsynonymous mutations in humans, *h* values above 0.15 all fit the human MIS-SFS well [49], supporting the use of *h* = 0.5 as a reasonable approximation. Nevertheless, incorrect assumptions about dominance could bias DFE parameter estimates, especially given that strongly deleterious mutations are predicted to be more recessive [42,50]. Although we controlled for selection at linked synonymous variants during demographic inference, direct selection can occur on synonymous variants and distort the SFS, through codon usage bias [51], selection on exonic splice enhancers [52], and GC-biased gene conversion (BGC) [24]. Repeating the DFE inferences on a filtered set of mutations unaffected by BGC resulted in slight differences in DFE estimates for mice, but far less pronounced than the contrasts observed across species (Fig U in S1 Appendix). A previous simulation study found that estimates of the nonsynonymous DFE remain reliable with small amounts of selection on synonymous mutations, unless the mean selection coefficient (*s*) for synonymous mutations is greater than 10^−4^ [53]. Additionally, variation of the DFE across species in non-coding regions remains to be explored. In humans, estimates suggest that approximately 7%–9% of the mutations in the noncoding genome are deleterious, with mutations being more deleterious in promoters than enhancers [54–56]. Conversely, in *Drosophila*, noncoding regions are responsible for the majority of new deleterious mutations within populations [57]. Overall, future studies are needed to jointly infer the DFE with dominance, and to explore whether the DFEs for non-coding and structural variants differ across species. Beyond these technical limitations described above, the DFE itself is a composite distribution as it averages over genetic and environmental backgrounds. The selection coefficients we are attempting to measure from polymorphism data are an aggregate measure of how the mutations affect phenotypes and how those phenotypes relate to fitness. Thus, differences in the inferred DFEs across species could be due to a variety of interacting molecular processes [58].

Despite the multifaceted nature of the DFE, we nevertheless found significant phylogenetic correlations during the evolution of the DFE in animals (Pagel’s λ = 0.84, Fig 1B), and the pairwise likelihood ratio tests showed that the DFE is more similar in species-pairs within the same taxonomic class (Fig 2E). Several studies have suggested that the DFE is similar in closely related species, but this phylogenetic signal has not been systematically evaluated until now. For example, correlated DFEs were reported between populations within species in humans, *Drosophila* and wild tomato [50]. This stability extends to experimental evolution, where the fitness effects of deleterious mutations in an *Escherichia coli* lineage remained remarkably similar over 50,000 generations [59]. In addition, stark DFE differences have been consistently observed between species with early divergences, such as humans and *Drosophila* [7,18]. Previous work comparing the DFE across diverse groups of animals had reported among-taxa variation [29,60], similar to our findings. Chen and colleagues [29] acknowledged the differences in DFE between invertebrates and vertebrates, whereas the uncertainties for |*s*|_mean_ in their study prevented further investigations. Although Galtier and Rousselle [60] considered the strong group effect on the shape parameter to be a methodological artifact and deemed the DFE to be the same across species, it was later shown that their model’s inclusion of lethal mutations flattened the variation across SFSs, and was unsuitable for fitting a single gamma DFE to multi-species data [61]. Recently, between-species, but not within-species, variation in the DFE was reported for six forest tree species, and 38 catarrhine primates, corroborating the phylogenetic constraints we found in the animal DFEs [62,63]. Emergent studies in comparative population genomics suggest that phylogenetic correlations exist not only in mutation’s fitness effects, as demonstrated here, but also in other fundamental population genetics parameters, such as mutation rates and mutational spectra in vertebrates [64,65]. Although our study did not find correlations of mutation rate with |*s*|_mean_ after correcting for phylogenetic signal (Fig Z in S1 Appendix), simulations have suggested a complex joint distribution of selection coefficients and mutation rates [66]. Finally, while our study was not designed as a comprehensive meta-analysis, we note that still relatively few non-model organisms across the tree of life have high-quality reference genomes and gene annotations, well-characterized mutation rates, and population-level high-coverage whole-genome data, which are essential for robust DFE inference. Expanding the availability of such datasets is important to distinguish the co-evolution of mutation rates and their effects, and to address core evolutionary hypotheses, such as adaptive evolution and Lewontin’s paradox [22,30,67].

Fisher’s geometric model (FGM) offers a simplistic, yet versatile, genotype-phenotype-fitness landscape to explain the mechanism underlying phylogenetic signals in the DFE variation across species. There are three lines of evidence suggesting that FGM is a reasonable model to explain the variation of the DFE across species. First, we found the DFEs derived under FGM assuming mutation-selection-drift equilibrium (eq. 3) fit the SFS of our 11 species. Second, a key prediction from FGM about how the DFE is expected to vary with population size $N_{a}$ is satisfied by our analysis. Specifically, as $N_{a}$ decreases, a species’ phenotype at mutation-selection-drift equilibrium is farther away from its fitness optimum, leading to the increased proportions of both beneficial and strongly deleterious mutations in smaller populations (Fig AL in S1 Appendix). While assuming FGM, we inferred the proportion of beneficial mutations to offset this drift load. This proportion of beneficial mutations was negatively correlated with the long-term population size (Fig 4C). Moreover, mammals with lower $N_{a}$ exhibited a markedly higher proportion of highly deleterious mutations compared to insects and birds when the deleterious component of the DFE was modeled by the gamma distribution (Fig 1D). As this trend was observed when not assuming FGM, it provides additional support for the FGM in explaining these observations. Third, another key prediction from FGM about how the DFE is expected to vary with species complexity is satisfied by our analysis. FGM predicts that the expected effects of mutations become more deleterious as organismal complexity increases, because mutations are more likely to disrupt something important when more dimensions are available in the phenotype space [7]. While biological complexity lacks a straightforward definition, evidence suggests that mammals are more complex than insects. This is demonstrated by their greater number of unique cell types, larger and more complex genomes, and more extensive protein-protein interactions and transcriptional regulatory networks (Text F in S1 Appendix). Indeed, we found that mutations are, on average, more deleterious in the more complex mammal species compared with insects (Fig 1). Of note, this pattern was present regardless of the functional form of the DFE assumed, and thus is not merely a trivial consequence of fitting FGM to the data. Thus, a number of analyses suggest that FGM can explain the differences in the DFE across species.

Assuming that Fisher’s geometric model explains the variation in the DFE across species, this model implies widespread macroscopic epistasis. In other words, a mutation’s fitness effect depends on the population’s background fitness [68,69]. Theoretically, a stable mutation-selection-drift equilibrium requires that the selective coefficients depend on the background fitness. To maintain stable fitness at equilibrium, the distribution of *s* needs to broaden as fitness declines [70]. With a log-concave fitness function, $w(z)=exp(-z^{2})$, where *z* is the distance to phenotype optimum, the FGM has a built-in mechanism of epistasis [7,14]. This leads to the prediction that the DFE variance increases as the equilibrium distance *z* increases in species with smaller ancestral population size and higher complexity (Fig AL in S1 Appendix). Evidence for macroscopic epistasis comes not only from DFEs derived from genetic variation data, as this study shows, but also from experimental findings. For example, the observation that larger populations have a lower proportion of beneficial mutations (Fig 4C), is consistent with the widely observed diminishing-return epistasis in experimental microbial evolution, where the strength of beneficial mutations quickly declines as populations gain fitness in laboratory settings [59,71].

To further understand the biological factors driving variation of the DFE across species, we tested for correlations between summaries of the DFE and various life-history traits. Overall, we found robust positive correlations between |*s*|_mean_ and genome size as well as |*s*|_mean_ and body mass. Nonsynonymous mutations in species with larger body mass or larger genomes tended to be more deleterious, on average. The correlation between |*s*|_mean_ and $N_{a}$ was less consistent across analyses and was no longer significant after correcting for multiple testing. Other traits, like recombination rates and longevity, showed little evidence of a correlation. These analyses should be interpreted with caution, as we sampled only a limited number of taxa. Thus, a lack of correlation could be due to insufficient statistical power. Further analyses focusing on additional taxa will be necessary to reliably disentangle which life history traits are independently associated with DFE variation. Nevertheless, a negative correlation between long-term population size and |*s*|_mean_ could be expected under conditions with pervasive epistasis. Natural selection is more efficient in larger populations than in smaller populations. As such, in larger populations, positive selection will be more effective at fixing mutations that increase protein stability, function, and robustness (Fig AM in S1 Appendix). When new deleterious mutations occur in large populations, these deleterious mutations occur in the optimal background and may have only weak effects. These are the mutations whose fitness effects have been measured in our present analysis. In smaller populations, however, selection will be unable to fine-tune the proteome (Fig AM in S1 Appendix), resulting in unstable proteins. When subsequent deleterious mutations occur in smaller populations, they occur on a less optimal background, and as such, they are more likely to be strongly deleterious. For non-coding mutations, we speculate that macroscopic epistasis could still apply, but through different mechanisms such as maintaining binding-site stability [72]. However, more non-coding DFE estimates across species are needed to formally test this hypothesis.

Our findings on the phylogenetic dependence of DFE variation have several practical implications. In conservation biology, genomics-informed simulations are gaining popularity to estimate the genetic health and quantify the deleterious load of small and fragmented populations [28,39,73,74]. The relative conservation of the DFE parameters within closely-related lineages suggests that it is reasonable to assume DFE parameters from closely-related species when the study species’ DFE parameter is not available. However, we caution against using constant DFE parameters across divergent lineages (e.g., mammals versus insects), as significant variation in the DFE exists. Our study also provides additional insights for adaptive evolution, by offering a phylogenetic constraint in the constant evolutionary processes of genetic drift and purifying selection, allowing for a more appropriate evolutionary null model for identifying adaptive variants [4].

Overall, we speculate that while $N_{a}$ and its changes are similar in closely-related species due to shared evolutionary histories, they are not inherently phylogenetically constrained. Long-term population size and demographic events significantly influence a population’s DFE by modifying the balance between selection and drift, therefore altering the fitness background. Conversely, organismal complexity reflects the physiological constraints imposed by phylogenetic structure, and probably governs the DFE at the mutation-phenotype mapping level through molecular mechanisms, such as protein stability and functional redundancy. Notably, we observed that mutations tend to be more deleterious in more complex mammal species (Fig 1), contradicting Kimura’s classical proposition that more mutations are neutral in more complex species [15]. In the future, we propose extending Fisher’s geometric model from a single-species to a multi-species model where organismal complexity across species is regulated by a hyperparameter that adjusts the degree of phylogenetic dependence. In conclusion, our study demonstrates strong phylogenetic correlations in the DFE variation across animals.

## Materials and methods

### Data sets and site frequency spectra

Polymorphism datasets from 11 animal (sub)species were compiled from various whole genome resequencing projects using a standardized curation workflow designed to retain only high-quality variants and samples for DFE inference (Text A in S1 Appendix). Most datasets had pre-annotated variant effects. If not, we annotated the variant call files (VCF) using default options in snpEff (v.5.1 [75]). To retain only high-quality variants, we selected genomic sites that: 1. passed the filters set in the original dataset; 2. were in canonical coding region (CDS) but not in soft-masked, unknown or CpG islands of the genome; 3. were monomorphic or biallelic single-nucleotide polymorphisms (SNP) that were annotated as “synonymous" or “nonsynonymous/missense". To retain only high-quality samples from as close to a panmictic population as possible, we selected at least eight diploid samples for each species that: 1. had at least 8x average sequencing depth; 2. belonged to one natural population with no population substructure or admixture identified by principal component analysis (PCA); 3. were not related to other samples identified by kinship analysis.

To summarize polymorphism data, the obtained VCFs were further filtered to exclude sites with more than 20% missing or 75% heterozygous genotypes. We projected down the sample size and computed folded synonymous and nonsynonymous/missense site frequency spectra (SYN-SFS and MIS-SFS, respectively) for each species. Computing a folded SFS mitigates uncertainties in ancestral state classifications. The projected sample size was calculated using the hypergeometric probability distribution to maximize the number of SNPs available and account for the sporadic missing genotypes (S1 Data). The total synonymous and nonsynonymous sequence lengths (*L*_SYN_ and *L*_MIS_) were obtained using previous estimates of nonsynonymous to synonymous mutation rate ratio $r_{L}=\frac{\theta_{\text{MIS}}}{\theta_{\text{SYN}}}$ [7].

We also compiled several life history traits and genomic attributes for each species from the literature (sources and citations in S1 Data).

### Demographic inference

To control for how population size changes influence the SFS, we first inferred demographic parameters from the SYN-SFS using the Demog1D_sizechangeFIM module in *varDFE* (v.0.1.0, Fig B in S1 Appendix, based on ∂a∂i v.2.1.1 [20,40] and Python v.3.10.2). For each species, we fitted four models, including the standard neutral, two-epoch, three-epoch, and four-epoch models. To account for the uncertainties in genotype calls, we repeated the demographic inference by masking the singleton entries in the SYN-SFS. In total, eight demographic inference runs (four demographic models with or without masking singletons) for each species were conducted. All settings were the same across datasets except for the starting parameter positions, which were set based on knowledge from prior demographic inference (S1 Data, S3 Data). For each run, the best-fit parameters with the maximum multinomial log-likelihood in 100 replicates were chosen, and their uncertainties were estimated through Fisher’s Information Matrix (FIM).

To choose the best-fit demographic model and singleton-masking treatment out of the eight runs per (sub)species for downstream DFE inference, we performed a stepwise likelihood-based model selection procedure (Fig D and Text A in S1 Appendix, S4 Data). At the start of model selection, the two-epoch demographic model based on full SYN-SFS run was chosen for all datasets. However, if it resulted in poor model fit, we chose a more complex demographic model or masked the singletons in the SYN-SFS. We calculated the population-scaled synonymous mutation rate $\theta_{\text{SYN}}$, using the optimal_sfs_scaling function in ∂a∂i. $\theta_{\text{SYN}}$ is only equivalent to Watterson’s $\theta_{w}$ under the standard neutral model, and changes with demographic model choice [40]. As exon mutation rates are hard to estimate in non-model organisms, and existing estimates can vary [64], we compiled a list of literature-reported mutation rates, and selected the most widely used, phylogenetic-based and whole-genome-based mutation rate estimates in each species (Text B in S1 Appendix). For each dataset, synonymous-site-based ancestral population size, or long-term population size, is defined as the reference population size in ∂a∂i, and is estimated using $N_{a}^{\text{SYN}}=\frac{\theta_{\text{SYN}}}{4\mu L_{\text{SYN}}}$ in the chosen demographic run for downstream analyses (Table 1).

### DFE inference

Conditional on the inferred demographic models, we estimated the DFE for new nonsynonymous mutations using methods from Fit∂a∂i [20]. Briefly, Fit∂a∂i takes advantage of the pattern that deleterious mutations are less likely to segregate in the sample of individuals and those that are segregating are more likely to be at lower frequency compared to neutral mutations. Assuming the MIS-SFS is under selection while the SYN-SFS is neutral, the best DFE parameters should fit the differences between MIS-SFS and SYN-SFS in each minor allele frequency bin. We first computed and stored the expected MIS-SFS for a range of population-scaled selection coefficients $\gamma =2N_{a}s$ (−10,000 ≤γ≤ −10^−5^ and 10^−5^ ≤γ≤ 100) in each species, given their best fit demographic scenarios, using the DFE1D_refspectra module in *varDFE*. Note that *varDFE* assumes semi-dominance of the mutations involved (*h* = 0.5), and *s* refers to the selection coefficient against heterozygotes.

To infer the deleterious-only DFE, we assumed that the DFE follows a gamma distribution, given its strong theoretical and empirical support [7,16,19]. We parameterized the DFE for each species as γ~Gamma(α,β), where α (α>0; shape) and β (β>0; scale) are parameters to be inferred. For each species, 100 replicates were run from a permuted starting parameter of α = 0.2 and β = 4,000 using the DFE1D_inferenceFIM module in *varDFE* [7]. The best-fit parameters with the maximum Poisson log-likelihood were chosen, and the corresponding Akaike information criterion (AIC) was calculated. To quantify uncertainty of the parameter estimates, we Poisson resampled the MIS-SFS 200 times, and repeated the DFE inference to obtain the 95% confidence interval [19,20]. Recall that we optimized the gamma distribution parameters for the population-scaled selection coefficient γ. To obtain the distribution of *s*, we unscaled the gamma distribution by $1/(2N_{a}^{\text{SYN}})$, therefore, $s\sim Gamma(\alpha ,\beta^{\prime })$, where $\beta^{\prime }=\beta /2N_{a}^{\text{SYN}}$ and $N_{a}^{\text{SYN}}$ had been inferred from demography estimation. The mean selection coefficient |*s*|_mean_ for each species was calculated given the gamma distribution’s property that $|s{|}_{\text{mean}}=\alpha \beta^{\prime }$. Similarly, $|2N_{a}s{|}_{\text{mean}}=\alpha \beta$. The median selection coefficient |*s*|_median_ was found as the value of *s* where the cumulative distribution function equals 0.5 using the pgamma function in R (v.4.4.2 [76]). To compute the proportion of mutations with different values of *s* or $2N_{a}s$, we found the cumulative probability in the given range using the pgamma function.

To estimate the proportion of effectively neutral mutations, we added a point mass at neutrality ($p_{\text{neu}}\geq 0$) to the original gamma distribution. The DFE function can be written as $\gamma \sim Neugamma(\alpha ,\beta ,p_{\text{neu}})$.


$$\begin{array}{c} f(\gamma )=\left\{ \begin{array}{cc} \frac{p_{\text{neu}}}{10^{-5}}+(1-p_{\text{neu}})*Gamma(\gamma |\alpha ,\beta ), & 0\leq |\gamma |<10^{-5}\\ (1-p_{\text{neu}})*Gamma(\gamma |\alpha ,\beta ), & |\gamma |\geq 10^{-5} \end{array}\right. \end{array}$$ (1)

The distribution of *s* is $s\sim Neugamma(\alpha ,\beta^{\prime },p_{\text{neu}})$, where $\beta^{\prime }=\beta /(2N_{a}^{\text{SYN}})$. The mean selection coefficient |*s*|_mean_ for each species is $|s{|}_{\text{mean}}=\frac{p_{\text{neu}}}{2*2N_{a}^{\text{SYN}}}*10^{-5}+(1-p_{\text{neu}})\alpha \beta^{\prime }$. The median selection coefficient |*s*|_median_ was found where the cumulative distribution function equals 0.5.

To examine the robustness of the DFE inference, we conducted additional analyses assessing the effects of the assumed functional form of the DFE, demographic model misspecification, singleton-masking treatments, mutation rate variation, differences in nonsynonymous to synonymous mutation rate ratio ($r_{L}$), and GC-biased gene conversion (detailed in Text D in S1 Appendix). To account for uncertainty in the functional form of the DFE, we performed DFE inference assuming three different functional forms (gamma, neugamma, and lognormal) under the best-fit demographic models and singleton masking treatments. For the lognormal distribution, we parameterized the DFE for each species as $\gamma \sim Lognormal(\mu_{s},\sigma^{2})$. The distribution of *s* was unscaled as $s\sim Lognormal(\mu_{s}^{{\;}^{\prime }},\sigma^{2})$, where $\mu_{s}^{{\;}^{\prime }}=\mu_{s}-ln(2N_{a}^{\text{SYN}})$. The median selective effects |*s*|_median_ for each species was calculated using the lognormal distribution’s property that $\left|s{|}_{\text{median}}=exp(\mu_{s}^{{\;}^{\prime }}\right)$.

### Phylogenetic signal detection

To evaluate whether the DFE co-varies with phylogeny, we calculated Pagel’s λ for the log10 transformed mean selection coefficient ($\log_{10}(|s{|}_{\text{mean}})$), the log10 transformed median selection coefficient ($\log_{10}(|s{|}_{\text{median}})$), and the shape parameter (α) in the gamma-distributed DFE, life history traits and genomic attributes using the phytools package (v.2.4.4 [77]). The underlying phylogenetic structure and divergence times for 11 (sub)species were compiled from the TimeTree database (http://timetree.org/). To test if the candidate life history traits and genomic attributes (*X*) are correlated with the observed DFE parameters, and correct for phylogenetic dependency when appropriate, we utilized the ${\text{PGLS}}_{\lambda }$ method in phytools. The regression was specified as $\log_{10}(|s{|}_{\text{mean}})\sim \log_{10}(X)$, $\log_{10}(|s{|}_{\text{median}})\sim \log_{10}(X)$, or $\alpha \sim \log_{10}(X)$ to normalize the data, except when *X* was genome size, in which case *X* was not log-transformed. The λ estimated in ${\text{PGLS}}_{\lambda }$ does not equate to the Pagel’s λ estimated individually for *X* or *Y*, but represents whether the correlation of *X* and *Y* can be accounted for by phylogenetic dependence in residual error. Candidate explanatory variables (*X*) include synonymous-site-based and literature-based long-term population size ($N_{a}^{\text{SYN}}$ and $N_{a}^{\text{LIT}}$) in diploids, body mass in grams, generation time in years per generation, age at maturity in days, maximum longevity in years, genome size in Mb, recombination rate in recombination events per bp per generation, and mutation rate in mutations per bp per generation. *P*-values were adjusted using a Bonferroni correction across the nine explanatory variables considered. To control for outliers or account for the long branches between mammals and insects, we repeated the Pagel’s λ and ${\text{PGLS}}_{\lambda }$ analysis excluding the arctic wolves, or only within mammals (S15 Data, S16 Data).

To formally test for variation in the DFE across species, we used a grid-search approach (DFE1D_gridsearch in *varDFE*) to compare each species’ DFE estimates to a null model where the DFE was constrained to be the same across species by likelihood-ratio tests (LRT). 2000 grid points were evenly spaced in biologically meaningful ranges for shape (α = 0.1 to 0.5) and scale ($\beta^{\prime }$ = 0.0001 to 0.5) parameters. Using integrate in ∂a∂i  we obtained the expected SFS for each $\alpha -\beta^{\prime }$ pair and calculated the Poisson log-likelihood relative to the empirical MIS-SFS in each species. For the null model, we summed log-likelihoods across species to find the MLE. To test whether the shape (α) and scale ($\beta^{\prime }$) are different in any *x* number of species, the LRT was constructed as $\Lambda =-2*ln(L_{0}/L_{1})=-2*(LL_{0}-LL_{1})$, where *LL*_0_ is the log-likelihood for the null model, with two DFE parameters inferred ($\hat{\alpha_{1}}=\hat{\alpha_{2}}=...=\hat{\alpha_{x}},\hat{\beta_{1}^{\prime }}=\hat{\beta_{2}^{\prime }}=...=\hat{\beta_{x}^{\prime }}$; params = 2) given the inferred demographic parameters for each species ($\Theta_{D,1},\Theta_{D,2},...,\Theta_{D,x}$), and *LL*_1_ is the log-likelihood for the alternate model, where each species is allowed to have its own DFE parameters ($\hat{\alpha_{1}},\hat{\alpha_{2}},\hat{\alpha_{x}},...,\hat{\beta_{1}^{\prime }},\hat{\beta_{2}^{\prime }},\hat{\beta_{x}^{\prime }}$; params = 2*x*). Across the 11 (sub)species tested, asymptotically, Λ should follow a $\chi^{2}$ distribution with df=2x−2=20. However, as Λ is a composite likelihood, its empirical distribution under the null model of constrained DFE parameters is broader than the $\chi^{2}$ distribution [7]. Although small *P*-values (e.g., *P* < 10^−16^) should not be interpreted literally, simulations showed that Λ did not exceed 34 under the null model in a two-species comparison, suggesting that values of Λ like those seen here would be unlikely under the null hypothesis [7].

We also calculated the pairwise LRT statistics in all possible pairs of species (*x* = 2). For example, the LRT statistics comparing humans (population 1) and *Drosophila* (population 2) DFE can be written as follows:


$$\begin{array}{c} \Lambda =-2*ln[\frac{L(\hat{\alpha_{1}=\alpha_{2}},\hat{\beta_{1}^{\prime }=\beta_{2}^{\prime }}|\Theta_{D,1},\Theta_{D,2})}{L(\hat{\alpha_{1}},\hat{\alpha_{2}},\hat{\beta_{1}^{\prime }},\hat{\beta_{2}^{\prime }}|\Theta_{D,1},\Theta_{D,2})}]. \end{array}$$ (2)

Hierarchical clustering for $\log_{10}(\Lambda )$ was performed by the hclust function in R. To test the significance of the variation of $\gamma =2N_{a}s$ across species, we repeated the LRT by evenly spacing 2000 grid points in shape (α = 0.1 to 0.5) and population-scaled scale (β = 100 to $2.5\times 10^{4}$) parameters. We also calculated Pagel’s λ for the log10 transformed population-level mean selection coefficient ($\log_{10}(|2N_{a}s{|}_{\text{mean}})$). We repeated ${\text{PGLS}}_{\lambda }$ analysis with $\log_{10}(|2N_{a}s{|}_{\text{mean}})$ as the response variable, following methods described above.

### Fitting the FGM-derived DFE

To investigate the genetic mechanisms underlying DFE variation, we implemented a functional form of the DFE directly derived from the Fisher’s geometric model [10]. The FGM proposes that fitness (*w*) can be described as a Gaussian function of the distance from the optimum (*z*), $w(z)=exp(-z^{2})$, in an *n*-dimension phenotypic space. Random mutations affect a subset of *m* phenotypes (m≤n), with fitness effect size *r* following a zero-mean Gaussian distribution with scale σ. The DFE for a population at mutation-selection-drift equilibrium can be described using the mutation pleiotropy (*m*), scale of mutation effects (σ) and long-term population size ($N_{a}^{\text{FGM}}$). As this DFE was derived originally by Lourenco and colleagues [10], we refer to it as $s\sim Lourenco(m,\sigma ,N_{a}^{\text{FGM}})$. Here Γ(.) is the gamma function and *K*(.) is the modified Bessel function of the second kind [10].


$$\begin{array}{c} f(s)=\frac{2^{\frac{1-m}{2}}\sqrt{N_{a}^{\text{FGM}}}(|s|{)}^{\frac{m-1}{2}}(1+\frac{1}{N_{a}^{\text{FGM}}\sigma^{2}}{)}^{\frac{1-m}{4}}exp(-N_{a}^{\text{FGM}}s)}{\sqrt{\pi }\sigma^{m}\Gamma (\frac{m}{2})}\times K_{\frac{m-1}{2}}(N_{a}^{\text{FGM}}|s|\sqrt{1+\frac{1}{N_{a}^{\text{FGM}}\sigma^{2}}}) \end{array}$$ (3)

While complex in its expression, this FGM-derived distribution is functionally equivalent to the previously described gamma and lognormal distributions for generating mutational effects. Therefore, we estimated its parameters (*m*, σ, and $N_{a}^{\text{FGM}}$) following methods described in the DFE inference section, through maximum-likelihood optimization by fitting the observed differences between MIS-SFS and SYN-SFS across minor allele frequency bins. To compute the proportion of mutations with different values of *s*, we found the cumulative probability for *s* in given ranges using the scipy.integrate.quad function (v.1.8.0) in Python. The proportion of beneficial mutations was obtained by calculating the cumulative probability for *s* > 0. We performed two ${\text{PGLS}}_{\lambda }$ regressions using the methods described above: first, $\log_{10}(N_{a}^{\text{FGM}})$ inferred in this FGM-derived DFE and the $\log_{10}(N_{a}^{\text{SYN}})$ obtained in demographic inference; second, the proportion of beneficial mutations and $\log_{10}(N_{a}^{\text{LIT}})$. To test for phylogenetic signal in the inferred *m* and σ parameters, we calculated Pagel’s λ (S15 Data).

### Simulating a human DFE with the *Drosophila* demography

To investigate whether Fit∂a∂i can accurately detect strongly deleterious mutations in species with large population sizes, we performed a simulation study using *Drosophila* demography and the human DFE. We used the stdpopsim library to obtain coding sequence (CDS) annotations for *Drosophila melanogaster* chromosomes 2L, 2R, 3L, and 3R from the FlyBase_BDGP6.32.51_CDS [78]. CDS regions were labeled as “exon", and non-coding regions as “bkgd". To simulate realistic genomic regions, we randomly sampled 108 1-Mb segments from these chromosomes with probabilities proportional to chromosome lengths. Annotations for each segment were subset and converted to SLiM’s coordinate format. Next, we implemented a two-epoch *Drosophila* demographic model in which the ancestral population size was set to 2,766,461, and the population expanded to 7,482,090 over 507,689 generations. We used a uniform recombination rate of $2.03806\times 10^{-8}$ which is an average of recombination rates for *Drosophila* chromosomes from the stdpopsim catalog. The mutation rate was set to $1.5\times 10^{-9}$ per bp per generation. Deleterious mutations followed a human-like gamma DFE (mean = –0.0276, shape = 0.189) with a nonsynonymous-to-synonymous ratio of 2.85. Background (noncoding) regions were assigned neutral mutations only. All mutations were additive (semi-dominant). From each simulation run on the randomly sampled 1-Mb *Drosophila* chromosome segments, we output SFS using a sample size of 100 or a sample size of 10. To reduce computational cost, we applied a scaling factor *Q* = 100, adjusting population size, mutation rate, recombination rate, and selection coefficients accordingly. One out of 108 simulations did not complete due to time constraints on the cluster and was discarded. To match the total length of chromosomes 2L, 2R, 3L, and 3R in *Drosophila*, we sampled the SFS outputs from 107 successful simulations with replacement, and summed 107 SFS outputs to generate one bootstrapped SFS that mimics a resampled version of the simulated *Drosophila* genome (~ 107 Mb). To assess inference uncertainty, we repeated the sampling 50 times, producing 50 bootstrapped SFSs for each sample size (2*n* = 100 or 2*n* = 10). We then inferred the DFE on the 50 bootstrapped SFSs as done for the empirical data. We assumed either a gamma-distributed or a lognormal-distributed DFE during inference. To evaluate the influence of selection at linked sites on synonymous mutations, we also inferred demographic parameters from the “bkgd" (non-coding) regions to compare with the demographic parameters inferred from synonymous mutations.

## Supporting information

S1 Appendix Supporting information Figs A–AM and Text A–F. The data and code needed to generate Figs A–AM can be found in https://zenodo.org/records/21802302.
(PDF)

S1 Data Polymorphism dataset and life history traits summary. (XLSX)

S2 Data Synonymous and missense site-frequency spectra built from each subspecies, and from SSWW-only sites in humans and mice. (TXT)

S3 Data Demographic inference summary. (TXT)

S4 Data Demographic inference model selection results. (TXT)

S5 Data Demographic inference comparisons with previous studies. (XLSX)

S6 Data DFE inference summary assuming that the DFE follows a gamma distribution. (TXT)

S7 Data DFE inference summary assuming that the DFE follows a neugamma distribution. (TXT)

S8 Data DFE inference summary assuming that the DFE follows a gammalet distribution. (TXT)

S9 Data DFE inference summary assuming that the DFE follows a lognormal distribution. (TXT)

S10 Data DFE inference summary assuming that the DFE follows a gamma distribution under different demographic model assumptions. (TXT)

S11 Data DFE inference summary assuming that the DFE follows a gamma distribution under different singleton-masking treatments. (TXT)

S12 Data DFE inference summary assuming that the DFE follows a gamma distribution with alternative mutation rates. (TXT)

S13 Data DFE inference summary assuming that the DFE follows a gamma distribution with alternative nonsynonymous-to-synonymous mutation rate ratios (*r_L_*). (TXT)

S14 Data DFE inference summary assuming that the DFE follows a gamma distribution in SSWW-only sites in humans and mice. (TXT)

S15 Data Pagel’s *λ* analysis summary. (TXT)

S16 Data Phylogenetic generalized least squares model summary. (TXT)

S17 Data DFE inference summary assuming that the DFE follows a Fisher’s geometric model-derived distribution (Lourenco DFE). (TXT)

S18 Data DFE inference comparisons with previous studies. (XLSX)

## Abbreviations

AIC: Akaike Information Criterion
BGC: biased gene conversion
CDS: coding sequence
DFE: distribution of fitness effects
FGM: Fisher’s Geometric Model
LRT: likelihood ratio test
MLE: maximum likelihood estimate
PCA: principal component analysis
SFS: site frequency spectrum
SNP: single-nucleotide polymorphisms
