# Supplementary material for: The distribution of fitness effects of nonsynonymous mutations varies phylogenetically across animals
Source: PLoS Biol. 2026 Sep 8;24(9):e3003976. doi: 10.1371/journal.pbio.3003976 (PMC13592725; doi:10.1371/journal.pbio.3003976)
Supplement: Supplementary file 1 [file pbio.3003976.s001.pdf]

Supporting Information for
The distribution of fitness effects of nonsynonymous mutations varies phylogenetically across animals

Meixi Lin[1,2,a], Sneha Chakraborty[1], Carlos Eduardo G. Amorim[3], Sergio F. Nigenda-Morales[4,5], Annabel C. Beichman[6], Paulina G. Nuñez-Valencia[4,7,b], Jonathan C. Mah[8], Jacqueline A. Robinson[9], Christopher C. Kyriazis[1,10], Christian D. Huber[11], Andrew E. Webb[2,12], Sarah D. Kocher[2,12], Frederick I. Archer[13], Andrés Moreno-Estrada[4], Robert K. Wayne[1,c], and Kirk E. Lohmueller[1,8,a]

[1]Department of Ecology and Evolutionary Biology, University of California, Los Angeles, Los Angeles, California, 90095, USA
[2]Department of Integrative Biology, University of California, Berkeley, Berkeley, California, 94720, USA
[3]Center for Evolution and Medicine, School of Human Evolution and Social Change, Arizona State University, Tempe, Arizona, 85287, USA
[4]Advanced Genomics Unit, National Laboratory of Genomics for Biodiversity (Langebio), Center for Research and Advanced Studies (Cinvestav), Irapuato, Guanajuato, 36824, Mexico
[5]Department of Biological Sciences, California State University San Marcos, San Marcos, California, 92096, USA
[6]Department of Genome Sciences, University of Washington, Seattle, Washington, 98195, USA
[7]Centro de Ciencias Genómicas, Universidad Nacional Autónoma de México (UNAM), Cuernavaca, Morelos, 62210, Mexico
[8]Bioinformatics Interdepartmental Program, University of California, Los Angeles, Los Angeles, California, 90095, USA
[9]Department of Ecology and Evolutionary Biology, Princeton University, Princeton, New Jersey, 08540, USA
[10]Conservation Genetics, San Diego Zoo Wildlife Alliance, Escondido, California, 92027, USA
[11]Department of Biology, Pennsylvania State University, University Park, Pennsylvania, 16802, USA
[12]Howard Hughes Medical Institute, Chevy Chase, Maryland, 20815, USA
[13]Marine Mammal and Turtle Division, Southwest Fisheries Science Center, La Jolla, California, 92037, USA
[a]Corresponding authors: meixilin@berkeley.edu; klohmueller@g.ucla.edu
[b]Current address: Centre for Genomic Regulation (CRG), The Barcelona Institute of Science and Technology, Barcelona, 08003, Spain; Universitat Pompeu Fabra (UPF), Barcelona, Spain.
[c]Deceased

August 22, 2026

This PDF file includes:
Text A to Text F
Fig A to Fig AM
SI References
Other supporting materials for this manuscript include the following:
S1 to S18 Data

A Text A: Materials and Methods

A.1 Data sets and initial processing

Polymorphism datasets from eleven animal (sub)species were compiled from various whole genome resequencing projects using a standardized curation workflow designed to retain only high-quality variants and samples for DFE inference (Fig B). To ensure variant quality and consistency, we began by selecting datasets with genotypes called using the GATK Best Practices Pipeline [1]. Most datasets had pre-annotated variant effects. For datasets without pre-built variant effect databases, which were halictid bees, collared flycatchers, pied flycatchers, and gray wolves, we annotated and predicted the effects of variants using default options in snpEff (v.5.1 [2]). To retain only high-quality variants, we selected genomic sites that: 1. passed the filters set in the original dataset; 2. were in the canonical coding region (CDS) but not in soft-masked, unknown, or CpG islands of the genome; 3. were monomorphic or biallelic single nucleotide polymorphisms (SNP) that are annotated as "synonymous" or "nonsynonymous/missense". To retain only high-quality samples from as close to a panmictic population as possible, we selected at least eight diploid samples for each species that: 1. had at least 8x average sequencing depth; 2. belonged to one natural population with no population substructure or admixture identified by principal component analysis (PCA); 3. were not related to other samples identified by kinship analysis.

To further understand the mechanisms of DFE evolution, we also compiled several life history traits and genomic attributes for each species from literature (sources and citations in Data S1). In some cases, the species' trait is not available, and the trait value from a closely related species was taken as a proxy. For example, the recombination rate of *Apis mellifera* was used a proxy for the recombination rate of halictid bees (Data S1).

The datasets for *Drosophila* [3], vaquitas [4], mice [5] and humans [3], had been quality controlled previously using similar methods described below and not described here.

A.1.1 Mosquitoes

For mosquitoes (*Anopheles gambiae coluzzii*), we queried the Ag1000G phase 3 (Ag3) release using the Python package `malariagen-data` (v.0.15.0 [6, 7]) on 2021-12-22 with custom Python scripts. We limited our search to a single-species *An. coluzzii* sample set (AG1000G-AO, n = 81) which were collected from breeding sites in Luanda, Angola in 2009 [8].

We first obtained coordinates of canonical CDS in autosomes (2R, 2L, 3R, 3L regions) by identifying the longest transcript of each gene. SNP calls were obtained and filtered using the `ag3.snp_call` method with the following criteria: 1. The sites passed `varian_filter_pass_gamb_colu` filters provided by the Ag3 release; 2. The sites fell into previously identified canonical CDS regions; 3. The sites were not in soft-masked or unknown regions of the genome (`a/t/c/g/N` bases in the genome sequence); 4. The sites were invariant or biallelic. Variants were annotated using the canonical CDS' annotations provided in `ag3.snp_effects`. The most deleterious annotation was retained for each variant. Only `SYNONYMOUS_CODING` (SYN) and `NON_SYNONYMOUS_CODING` (MIS) variants were downloaded as variant call format (VCF) files.

We performed PCA (function `snpgdsPCA`) and kinship analyses (function `snpgdsIBDKING`) for the LD-pruned SYN-VCF using the R package SNPRelate (v.1.16.0 [9]) to evaluate population structure. In total, 69 individuals SNP-only SYN-VCF and MIS-VCF were retained for downstream site frequency

spectrum projection, and 12 individuals were excluded due to high kinship (n = 9; kinship > 0.15) or being outliers in PCA analyses [n = 3; outliers defined as $\text{abs}(x - \text{median}(x)) > (6 * \text{sd}(x))$ in the PCA].

A.1.2 Halictid bees

For halictid bees (*Lasioglossum calceatum*), whole genome sequencing data were generated for 40 diploid females collected from two populations in the UK (north and south, n = 17 and 23 respectively). Reads were mapped to the chromosome-length *Lasioglossum calceatum* reference genome (LCAL2.1.1 [10]) and variants were called following the GATK Best Practices Guide [1].

A custom snpEff (v.5.1) database for LCAL2.1.1 was built from the gff3 file using default settings. We annotated and predicted the effects of variants using default options in snpEff. The most deleterious annotation was retained for each variant. Custom variant filtration was performed to retain only high-quality samples and genomic sites, with the following criteria: 1. The sites were within assembled autosomes; 2. The sites passed GATK-recommended hard filters (`QD < 2.0 || FS > 60.0 || MQ < 40.0 || MQRankSum < -12.5 || ReadPosRankSum < -8.0 || SOR > 3.0`); 3. The sites were outside of repeat regions identified by RepeatMasker [11]; 4. The sites had a sequencing depth of no more than 1200 or no less than 180; 5. The sites were invariant or biallelic. For the 153,244,615 sites that passed the above filters out of 282,627,975 total sites, we performed genotype-level filtration. Specifically, for each individual, genotypes with a minimum depth of four reads, a maximum depth of 2.5x mean depth or 30 reads (whichever is higher), and a minimum Phred score of six were kept. Genotypes that failed filtration were converted to missing. Individuals (n = 20 out of 40, $n_{\text{south}} = 9$, $n_{\text{north}} = 11$) with less than 20% of missing genotypes after genotype-level filtration were kept for downstream population structure analysis.

We investigated population structure by performing PCA and kinship analyses as described above for mosquitoes using all LD-pruned SNPs. The samples collected from the north and south populations separated in the PCA and had low kinship overall. We chose the north population for DFE inference. SNP-only SYN-VCF and MIS-VCF files for eleven individuals from the north population of halictid bees in the UK were retained for downstream SFS-based analyses.

A.1.3 Pied flycatchers and collared flycatchers

For pied flycatchers (*Ficedula hypoleuca*) and collared flycatchers (*Ficedula albicollis*), we downloaded filtered SNP-only VCF files for four flycatcher species from Dryad on 2022-03-24 [12]. This dataset was derived from a study assessing the genomic differentiation landscapes in Ficedula species pairs [13]. In this dataset, hard filter thresholds, repeat region masks, and genotype filters (`minDP = 5, maxDP = 200, minGQ=30`) had been applied [12].

We lifted the scaffold names and positions (`CHR` and `POS` fields) from the provided VCF file to the NCBI RefSeq chromosome coordinates using custom Python scripts (FicAlb1.5, `https://ftp.ncbi.nlm.nih.gov/genomes/all/GCF/000/247/815/GCF_000247815.1_FicAlb1.5/GCF_000247815.1_FicAlb1.5_assembly_structure`, accessed 2022-03-26). The lifted `REF` and `ALT` bases were verified using `BioPython` (v.1.79) and discordant sites were removed (0.01%).

We investigated population structure by performing PCA and kinship analyses as described above using all LD-pruned SNPs. A custom snpEff (v.5.1) database for FicAlb1.5 was built from the gtf file using default settings. We annotated and predicted the effects of variants using default options in snpEff. The most deleterious annotation was retained for each variant. Only `synonymous_variant` (SYN) and `missense_variant` (MIS) variants in assembled autosomes (chr1 to chr28) were retained. We additionally

masked sites (13.2%) that fell into repeat regions identified by WindowMasker (soft-masked bases in RefSeq genome; [14]) and CpG islands identified by UCSC genome browser [15].

SNP-only SYN-VCF and MIS-VCF files for nine individuals from the pied flycatcher population in mainland Sweden [16], were retained for downstream SFS analyses, and no individual was excluded. For the collared flycatcher population (n = 95) in Baltic Sea island Öland, Sweden [16], 88 individuals SNP-only SYN-VCF and MIS-VCF were retained for downstream SFS analyses, and seven individuals were excluded due to high kinship (n = 4; kinship > 0.15) or being outliers in the PCA [n = 3; outliers defined as $abs(x - median(x)) > (6 * sd(x))$ in PCA].

A.1.4 Arctic wolves

For arctic wolves (*Canis lupus arctos*), whole genome sequencing data were generated for 15 individuals collected from the arctic wolf population in Canada previously published [17, 18] and in this study. Variant calling was performed as described in previous studies [17, 18]. Only sites that passed variant quality filtration and did not fall in repeat regions or CpG islands were retained for this study. Variants were annotated using snpEff v.4.3.1, based on the dog reference genome annotation build CanFam3.1.75 available with snpEff installation. We considered sites where all three possible nucleotide changes were annotated as either `synonymous_variant` or `missense_variant` exclusively, and discarded sites with additional types of annotations (e.g., splicing sites and protein-truncating variants, such as stop-gained variants).

We estimated kinship and excluded relatives up to 4th degree (e.g. first cousins) using the KING-robust estimator [19] implemented in PLINK [20]. SNP-only SYN-VCF and MIS-VCF files for 14 individuals (n = 14) were retained for downstream SFS analyses, and one individual (n = 1) was excluded due to high kinship.

A.1.5 Gray wolves

For gray wolves (*Canis lupus lupus*), we downloaded filtered biallelic SNP-only VCF files for worldwide wolf and dog populations from Dryad on 2022-09-20 [21]. This dataset was derived from a study assessing dog introgression in the Fennoscandian wolf populations [22]. In this dataset, hard variant filters as recommended by the GATK Best practices Alternative protocol 2, in addition to variant depth, missingness and minor allele frequency filters, and genotype level filters (`minGQ = 30`) had been applied.

We validated the population structure reported in [22] by performing PCA and kinship analyses as described above using all LD-pruned SNPs. We annotated and predicted the effects of variants using default options in snpEff (v.5.1) based on the pre-built CanFam3.1.99 database (downloaded on 2022-10-10). The most deleterious annotation was retained for each variant. Only `synonymous_variant` and `missense_variant` variants in assembled autosomes (chr1 to chr38) were kept. We also masked sites that fell into repeat and low complexity regions (soft-masked bases in Ensemble genome release 99) and CpG islands identified by UCSC genome browser [15].

The Russian Karelian gray wolf population was selected for downstream SFS projection because of its high sequencing coverage (mean 30x), relatively large sample size (n = 15), clear clustering on the PCA, and historically large population size. SNP-only SYN-VCF and MIS-VCF files for thirteen individuals in this population were generated and two individuals were excluded due to high kinship ($kinship > 0.15$).

A.1.6 Fin whales

For fin whales (*Balaenoptera physalus*), we previously obtained VCF files from a historically stable population (n = 30) in the Eastern North Pacifics [23]. Only sites that passed variant quality filtration and did not fall in repeat regions or CpG islands were retained for this study.

Variants were annotated using SIFT4G v.6.0 [24] and snpEff v.4.3.1. The concordance of variant annotations was confirmed across the two software programs, and the most deleterious annotation derived from SIFT4G was used for each SNP.

We performed PCA and kinship analyses using all LD-pruned SNPs to evaluate population structure [23]. SNP-only SYN-VCF and MIS-VCF files for 27 individuals (n = 27) were retained for downstream SFS analyses, and three individuals were excluded due to admixture with another population (n = 2) or low genotyping rate (n = 1).

A.2 Calculation of site frequency spectra and sequence lengths

We summarized polymorphism data using the synonymous and nonsynonymous/missense site frequency spectra (SYN-SFS and MIS-SFS, respectively). SFS generation for *Drosophila* [3], vaquitas [4], mice [5] and humans [3], utilized similar methods as described below and in previous publications.

Here we describe the SFS generation procedures for mosquitoes, halictid bees, flycatchers, wolves and fin whales. Each SNP-only SYN-VCF and MIS-VCF file that passed the previously described quality control steps, was further filtered. Sites with more than 20% missing or 75% heterozygous genotypes were excluded using the GATK (v.3.8) `SelectVariants` [25] or bcftools (v.1.9) `filter` functions [26]. We projected down the sample size and computed folded SYN-SFS and MIS-SFS from SYN-VCF and MIS-VCF for each species using a modified `easySFS` module (`https://github.com/isaacovercast/easySFS`) and `make_data_dict_vcf`, `from_data_dict` methods in the $\partial a \partial i$ (v.2.1.1) package [27]. Computing a folded SFS mitigates uncertainties in ancestral state classifications. The projected sample size was calculated using the hypergeometric probability distribution to maximize the number of SNPs available and account for the sporadic missing genotypes (Data S1).

To calculate the total synonymous and nonsynonymous sequence lengths (L_{SYN} and L_{MIS}), we first obtained the length of coding regions (L_{CDS}) for each species. For mosquitoes, halictid bees, fin whales, and arctic wolves, variants had been called for all sites, including monomorphic sites, in the genome. We intersected the coordinates of coding regions with the sites that passed filters and had allele counts (`INFO/AC`) no less than the projected SFS sample size in each species' all-sites VCF (e.g. `INFO/AC` $\geq$ `136` for mosquitoes). For flycatchers, because the all-sites VCF is not available, we calculated an approximated L_{CDS} (11.8Mb) by multiplying the total CDS length (24.6Mb) from gtf file, with the proportion of callable sites (76.8%) reported for the autosomes (Table S1 in [12]), and the proportion of SNPs (62.7%) passing the additional filters applied during dataset QC. For gray wolves, neither all-sites VCF nor the proportion of callable sites were available as provided [22, 28]. Therefore, we utilized the proportion of callable sites reported in the Arctic wolves dataset as an approximate proxy.

For all species, we then calculated the synonymous and nonsynonymous sequence lengths (L_{SYN} and L_{MIS}; $L_{\mathrm{SYN}} + L_{\mathrm{MIS}} = L_{\mathrm{CDS}}$) from previous estimates of nonsynonymous to synonymous mutation rate ratio $r_L = \frac{\theta_{\mathrm{MIS}}}{\theta_{\mathrm{SYN}}}$. We used an r_L of 2.31 for vertebrates ($L_{\mathrm{MIS}} = 2.31 * L_{\mathrm{SYN}}$; $L_{\mathrm{MIS}} = \frac{2.31}{2.31+1} * L_{\mathrm{CDS}}$), and an r_L of 2.85 for invertebrates [3].

A.3 *varDFE* package description

We developed robust DFE comparison software, *varDFE*, as an extension for the $\partial a \partial i$ and Fit$\partial a \partial i$ packages. The *varDFE* package can be installed through `pip` in any Python environment (version $\geq$ 3.10). Our workflow consists of four main modules (Fig B):

1. `Demog1D_sizechangeFIM`: Demographic inference on putatively neutral SFS data.
2. `DFE1D_refspectra`: Precomputation of a reference SFS database under selection, given all possible selection coefficients s and the inferred demographic scenarios in step one.
3. `DFE1D_inferenceFIM`: DFE inference on selected SFS data, given the assumed functional form of the DFE and the pre-computed reference SFS database in step two.
4. `DFE1D_gridsearch`: DFE parameter space exploration through a grid search, given the assumed functional form of the DFE and the pre-computed reference SFS database in step two.

Similar to `dadi-cli`(`https://dadi-cli.readthedocs.io/en/latest/`), each module in *varDFE* offers a streamlined and easy-to-use command line tool that simplifies the application of $\partial a \partial i$ in research settings. In addition, our package extends the functionality of $\partial a \partial i$ with the ability to explore continuous positive selection coefficients, infer parameters for a greater range of functional forms of the DFE, and automate a grid search for optimal parameter values when considering multiple datasets. It also provides automatic quality control features, including plotting, convergence testing, and quantifying uncertainty using Fisher's Information Matrix.

Compared with DFE-alpha and polyDFE, *varDFE* excels in its flexibility to model complex demographic histories and to fit a wide range of distributions for the DFE. DFE-alpha is restricted to two- or three-epoch demographic models, with the three-epoch model often computationally demanding [29]. PolyDFE does not fit demographic models but accounts for demography via nuisance parameters [30, 31]. DFE-alpha only allows for gamma-distributed DFE functions, and polyDFE implements only gamma or discrete distributions for the deleterious DFE. In addition, automatic quality-control procedures and a modular design in *varDFE* further streamline complex DFE inference in multi-species analyses, enabling efficient replication of analyses across parameter sets and datasets. However, although *varDFE* can be extended to infer the full DFE including beneficial mutations segregating in the population, it does not incorporate divergence data as input, unlike DFE-alpha and polyDFE. Consequently, *varDFE* cannot estimate α, the proportion of nonsynonymous substitutions fixed by positive selection.

In the methods sections below, we illustrate the usage of the *varDFE* workflow and specific settings used in this study.

A.4 Demographic inference

We first inferred demographic parameters from the putatively neutral synonymous SFS (SYN-SFS) using the `Demog1D_sizechangeFIM` module in the *varDFE* package. For each species, we fit four models (parameters are described moving forward in time):

1. snm or one-epoch: standard neutral model with no population size change.
2. two-epoch: single population model with one size change event. The ratio of the current population size, i.e. the first size change, to the ancestral population size (`nua`) and the duration of size change (`Ta`, in units of $2N_a^{\mathrm{SYN}}$ generations) are inferred.
3. three-epoch: single population model with two size change events. The ratio of the first size change population size, to the ancestral population size (`nua`), the ratio of the current population size, i.e.

the second size change, to the ancestral population size (`nub`), and the duration of two size change events (`Ta` and `Tb`) are inferred.

4. four-epoch: single population model with three size change events. The ratio of the first size change population size to the ancestral population size (`nua`), the ratio of the second size change population size to the ancestral population size (`nub`), the ratio of the current population size, i.e. the third size change, to the ancestral population size (`nuc`), and the duration of three size change events (`Ta`, `Tb` and `Tc`) are inferred.

To account for increased uncertainty in calling genotypes of rare variants, we also repeated the demographic inference by masking the singleton entries in the SYN-SFS. In total, eight demographic inference runs (four demographic models with or without singleton masks) for each species were conducted. All settings were the same across datasets except for the starting parameter positions, which were based on prior demographic inference (Data S1, S3). For datasets without prior demographic inference information (halictid bees, collared flycatchers, arctic wolves and gray wolves), we set the parameters to `nua, nub, nuc, Ta, Tb, Tc = 1` for initial runs and used the inferred demographic parameters as the starting positions in the final runs.

For each run, 100 replicates from permuted starting parameters (`fold=1`) were run. Within each of the hundred replicates, we set the extrapolation grid points as sample sizes plus 5, 15, and 25 [27], the maximum number of iterations at 100, and performed parameter optimization using the `optimize_log` function based on the multinomial log-likelihood calculated from the expected SFS in each iteration. The best-fit parameters with the maximum multinomial log-likelihood in the 100 replicates were chosen for each run. We examined the convergence of the inference across replicates with different starting values by calculating the difference in log-likelihood in the 20 replicates with the highest log-likelihoods. The best-fit parameters' uncertainties were estimated through Fisher's Information Matrix (FIM, `Godambe.FIM_uncert` function in $\partial a \partial i$). To evaluate the best-fit parameters in the eight runs per species, we plotted each best-fit expected SFS with the observed SYN-SFS using ggplot2 (v.3.5.1 [32]) in R (v.4.4.2 [33]).

We calculated the population-scaled synonymous mutation rate θ_{SYN}, using the `optimal_sfs_scaling` function in $\partial a \partial i$. θ_{SYN} is only equivalent to Watterson's θ_w under the standard neutral model, and changes with demographic model choice [27]. It is important to note that the exon mutation rate is hard to estimate in non-model organisms, and existing estimates can vary [34]. To minimize the uncertainty in μ, we compiled a list of literature-reported mutation rates, and selected the most widely used, phylogenetic-based and whole-genome-based mutation rate estimates in each species (Original rate in Text B). For each dataset, synonymous-site-based ancestral population size, or long-term population size, is defined as the reference population size in $\partial a \partial i$, and is estimated using $N_a^{\mathrm{SYN}} = \frac{\theta_{\mathrm{SYN}}}{4\mu L_{\mathrm{SYN}}}$ in the chosen demographic run for downstream analyses (Table 1). L_{SYN} is the previously estimated synonymous region sequence length.

To choose the best-fit demographic model and singleton-masking treatment out of the eight runs per (sub)species for downstream DFE inference, we performed a step-wise model selection procedure (Fig D). To maximize the data available and use the most parsimonious model whenever possible, at the start of model evaluation, the two-epoch demographic model based on the full (unmasked) SYN-SFS run was chosen for all datasets. This "two-epoch, full SFS" run was chosen for a dataset, if it met all the criteria below:

1. The log-likelihood differences between the data (SYN-SFS) and model maximum likelihood $\Delta LL = LL_{\mathrm{data}} - LL_{\mathrm{demographic\ model}}$ for demographic inference is less than 200.

2. Based on the inferred demographic parameters from the evaluated run ("two-epoch, full SFS" in this case), the log-likelihood differences between the data (MIS-SFS) and model maximum likelihood $\Delta LL = LL_{\text{data}} - LL_{\text{DFE model|demographic model}}$ for the gamma DFE inference is less than 200.
3. The parameter estimates in the current demographic model are realistic. (unrealistic parameters defined as `nu` $< 10^{-5}$ or `nu` > 20 or `T` $< 10^{-5}$ or `T` > 9).

Otherwise, we proceeded to evaluate the more complex three- or four-epoch models with the same criteria. If the full/unmasked SFS resulted in poor model fit across all tested demographic models, we proceeded to mask the singletons, and continued the evaluation until one run met all criteria. This approach allows us to balance the risks of model overfitting and data loss, while ensuring a good model fit for each individual species.

Using this approach, the most parsimonious "two-epoch, full SFS" run was suitable for most species, except for the collared flycatchers, gray wolves, mosquitoes, and halictid bees datasets. For the collared flycatchers, the "three-epoch, full SFS" run was chosen based on the first criterion, as the fit of demographic models greatly improved over the "two-epoch, full SFS" run ($\Delta LL_{\text{two-epoch}} = 1517$ and $\Delta LL_{\text{three-epoch}} = 1.35$). For the gray wolves, the "three-epoch, full SFS" run was chosen based on the third criterion, as the parameter estimates were extreme for the "two-epoch, full SFS" run (`nua` $= 2.27$, `nub` $= 0.30$, `Ta` $= 1.59$, `Tb` $= 0.039$ for the three-epoch model and `nua` $= 8.5 \times 10^{-5}$, `Ta` $= 4.5 \times 10^{-6}$ for the two-epoch model). For mosquitoes and halictid bees, all models based on the unmasked SFS failed to provide a good fit due to various reasons. The "three-epoch, masked SFS" run passed all criteria for mosquitoes and the "two-epoch, masked SFS" run was selected for halictid bees (details can be found at Data S4).

For the fin whales and vaquitas datasets, additional demographic estimates derived from non-coding sites far from genes in highly recombining regions are available, utilizing the same pipelines described herein [4, 23]. The inferred N_a values are directly comparable and exhibit high concordance. In vaquitas, N_a is 4485 when inferred from non-coding variants, and 6264 when inferred from synonymous variants. In fin whales, N_a is 16475 when inferred from non-coding variants, and 12411 when inferred from synonymous variants.

A.5 DFE inference

Conditional on the inferred demographic models, we estimated the DFE for new nonsynonymous mutations based on the MIS-SFS for each species using methods from Fit$\partial a \partial i$ [35]. Briefly, Fit$\partial a \partial i$ takes advantage of the pattern that deleterious mutations are less likely to segregate in the sample of individuals and those that are segregating are more likely to be at lower frequency compared to neutral mutations. Assuming the MIS-SFS is under selection while the SYN-SFS is putatively neutral, the best DFE parameters should fit the differences between MIS-SFS and SYN-SFS in each minor allele frequency bin. Two modules in *varDFE*, `DFE1D_refspectra` and `DFE1D_inferenceFIM`, provide a flexible workflow to estimate both the full and deleterious-only DFE with any given demographic and DFE models. To ensure consistency across species, during DFE inference, we only allowed species-specific settings including demographic parameters, mutation rates (μ), length of nonsynonymous region (L_{MIS}), population-scaled mutation rates (θ) to vary across species.

We first computed and stored the expected SFS for a range of population-scaled selection coefficients $\gamma = 2N_a s$, given the best-fit demographic scenarios using the `DFE1D_refspectra` module in *varDFE*. Note that *varDFE*assumes semi-dominance of the mutations involved ($h = 0.5$), and s refers to the selection

coefficient against heterozygotes. Here we calculated the reference spectra given γ but not s, because the SFS reflects the effect of selection at population level, which depends on $\gamma = 2N_a s$ but not s alone. We implemented a slightly modified `Cache1D_mod` module in $\partial a \partial i$, named `Cache1D_mod2`, with a method to integrate over continuous positive gamma space. The range of positive gammas was set from $+10^{-5}$ to $+100$, with 701 points evenly distributed on the log10 scale, and negative gammas from -10000 to -10^{-5}, with 901 points evenly distributed on the log10 scale. The extrapolation points (`pts_l`) were set as `[1000,1200,1400]`. The reference spectra, from which the expected SFS given any γ can be computed, were cached to save time for recalculations and improve consistency.

A.5.1 DFE inference assuming the gamma DFE

To infer the deleterious-only DFE ($s \leq 0$), we assumed that the DFE follows a gamma distribution and performed the inference using the `DFE1D_inferenceFIM` module. We used the gamma distribution because it had strong support from theoretical models, such as the FGM [36], and was shown to fit the data well in previous studies [3, 37]. We parameterized the DFE for each species as $\gamma \sim Gamma(\alpha, \beta)$, where α ($\alpha > 0$; shape) and β ($\beta > 0$; scale) are parameters to be inferred.

To avoid finding local maxima, for each species, 100 replicates were run from a permuted starting parameter (fold=1) of $\alpha = 0.2$ and $\beta = 4000$, informed from prior DFE estimations [3]. We set parameter upper bounds at $\max(\alpha) = 2.0$ and $\max(\beta) = 10^6$, lower bounds at $\min(\alpha) = 0.001$ and $\min(\beta) = 0.01$, and the maximum number of iterations at 100. The population-scaled nonsynonymous mutation rate θ_{MIS} was pre-calculated from the θ_{SYN} in the best-fit demographic model, using a scaling factor r_L of 2.31 for vertebrates and r_L of 2.85 for invertebrates [3]. We performed parameter optimization using the `optimize_log` function based on Poisson log-likelihood calculated from the expected SFS, generated using the `integrate` method from the precomputed reference spectra, in each iteration.

The parameters with the maximum Poisson log-likelihood in the 100 replicates were chosen for each run. We evaluated replicate convergence across starting values by calculating the difference in log-likelihood in the 20 replicates with the highest log-likelihoods. The AIC for each run was calculated using the equation: $AIC = 2k - 2ln(\hat{L})$, where $ln(\hat{L})$ is the highest log-likelihood associated with the best-fit parameters in DFE inference, and k is the total number of estimated parameters for the DFE and underlying demographic model. For example, $k = 5$ in a DFE inference run assuming a gamma-distributed DFE and a two-epoch demographic model, because two parameters (shape and scale) are inferred during DFE inference, and three parameters (`nua`, `Ta`, and θ_{SYN}) are inferred during demographic inference. Recall that we optimized the gamma distribution parameters for the population-scaled selection coefficient γ. To obtain the distribution of s, we unscaled the gamma distribution by $1/(2N_a)$, therefore, $s \sim Gamma(\alpha, \beta')$, where $\beta' = \beta/2N_a^{\text{SYN}}$ and N_a^{SYN} had been inferred from demography estimation.

The mean selection coefficient $|s|_{\text{mean}}$ for each species was calculated from the best-fit parameters given the gamma distribution's property that $|s|_{\text{mean}} = \alpha\beta'$. The median selection coefficient $|s|_{\text{median}}$ was found as the value of s where the cumulative distribution function equals 0.5 using the `pgamma` function in R (v.4.4.2 [33]). To compute the proportion of mutations with different values of s in the maximum-likelihood gamma distribution, we found the cumulative probability for $|s|$ ranged in [0, 10^{-5}), [10^{-5}, 0.0001), [0.0001, 0.001), [0.001, 0.01), and [0.01, 0.5] using the `pgamma` function in R. The mean population-scaled mutation effects ($|2N_a s|_{\text{mean}}$) were calculated as $|2N_a s|_{\text{mean}} = \alpha\beta$. The cumulative probability for $|2N_a s|$ ranged in [0,1), [1, 10), [10, 100), [100, 1000) and [1000, Inf] was calculated using the `pgamma` function in R as well. To quantify statistical uncertainty, we followed the methods described in [35,

37]. Briefly, for each species, we Poisson resampled the MIS-SFS 200 times, and refit the DFE on the resampled data. We calculated the mean selection coefficients, and the proportion of mutations with different values of s using these 200 estimates. The 95% confidence intervals were determined using the 2.5% and 97.5% quantiles of the resampled estimates.

A.5.2 Inferring the proportion of neutral mutations

To estimate the proportion of effectively neutral mutations, we added a point mass at neutrality to the original gamma DFE distribution. The DFE function can be written as $\gamma \sim Neugamma(\alpha, \beta, p_{\text{neu}})$. α ($\alpha > 0$; shape) and β ($\beta > 0$; scale) are parameters from a gamma distribution, and p_{neu} ($p_{\text{neu}} \geq 0$) is the proportion of neutral mutations ($0 \leq |\gamma| < 10^{-5}$).

$$f(\gamma) = \begin{cases} \frac{p_{\text{neu}}}{10^{-5}} + (1 - p_{\text{neu}}) * Gamma(\gamma|\alpha, \beta), & 0 \leq |\gamma| < 10^{-5} \\ (1 - p_{\text{neu}}) * Gamma(\gamma|\alpha, \beta), & |\gamma| \geq 10^{-5} \end{cases} \tag{1}$$

During inference, we set the parameter start positions, upper bounds and lower bounds in the same way as we did for the gamma distribution for the α and β parameters. The starting position for p_{neu} is 0.3, the upper bound is $\max(p_{\text{neu}}) = 1$, and the lower bound is $\min(p_{\text{neu}}) = 10^{-5}$.

To obtain the distribution of s, we unscaled the neugamma distribution by $1/(2N_a)$, therefore, $s \sim Neugamma(\alpha, \beta', p_{\text{neu}})$, where $\beta' = \beta/(2N_a^{\text{SYN}})$ and N_a^{SYN} had been inferred from the demographic model. The mean selection coefficient $|s|_{\text{mean}}$ for each species was calculated from the best-fit parameters using $|s|_{\text{mean}} = \frac{p_{\text{neu}}}{2*2N_a} * 10^{-5} + (1 - p_{\text{neu}})\alpha\beta'$. The median selection coefficient $|s|_{\text{median}}$ was found as the point where the cumulative distribution function equals 0.5.

A.5.3 Inferring the proportion of lethal mutations

To estimate the proportion of effectively lethal mutations, we added a point mass at lethality to the original gamma DFE distribution. The DFE function can be written as $\gamma \sim Gammalet(\alpha, \beta, p_{\text{let}})$. α ($\alpha > 0$; shape) and β ($\beta > 0$; scale) are parameters from a gamma distribution, and p_{let} ($0 \leq p_{\text{let}} < 0.5$) is the proportion of lethal mutations ($|s| = 0.5$).

$$f(\gamma) = \begin{cases} (1 - p_{\text{let}}) * Gamma(\gamma|\alpha, \beta), & 0 \leq |\gamma| < N_a \\ p_{\text{let}}, & |\gamma| = N_a \end{cases} \tag{2}$$

During inference, we set the parameter start positions, upper bounds and lower bounds in the same way as we did for the gamma distribution for the α and β parameters. The starting position for p_{let} is 0.001, the upper bound is $\max(p_{\text{let}}) = 0.5$, the lower bound is $\min(p_{\text{let}}) = 10^{-5}$.

To obtain the distribution of s, we unscaled the gammalet distribution by $1/(2N_a)$, therefore, $s \sim Gammalet(\alpha, \beta', p_{\text{let}})$, where $\beta' = \beta/(2N_a^{\text{SYN}})$ and N_a^{SYN} had been inferred from demography estimation. The mean selection coefficient $|s|_{\text{mean}}$ for each species was not calculated for this distribution, given the presence of lethal mutations.

A.6 Assessing the robustness of DFE inference

To examine the robustness of our DFE inference, we implemented three functional forms of the DFE in addition to the gamma distribution: neugamma, gammalet, and lognormal. We kept the same demographic

models and singleton masking treatments as used in the original analysis shown in Table 1. The functional forms for the neugamma and gammalet distributions have been described above. For the lognormal distribution, we parameterized the DFE for each species as $\gamma \sim Lognormal(\mu_s, \sigma^2)$. During inference, we set the starting positions before perturbation as $\mu_s = 1, \sigma = 0.1$, upper bounds as $\max(\mu_s) = 100$, $\max(\sigma) = 100$, lower bounds as $\min(\mu_s) = -100$, $\min(\sigma) = 10^{-5}$. Since μ_s could take negative values, we used the `optimize` instead of `optimize_log` function for parameter optimization. To obtain the distribution of s, we unscaled the lognormal distribution, therefore, $s \sim Lognormal(\mu_s^{'}, \sigma^2)$, where $\mu_s^{'} = \mu_s - ln(2N_a^{\text{SYN}})$ and N_a^{SYN} had been inferred from demography estimation. Median mutation effects $|s|_{\text{median}}$ for each species was calculated given the lognormal distribution's property that $|s|_{\text{median}} = exp(\mu_s^{'})$ [3].

We investigated whether the specific choices of the demographic models influenced our results. The reference spectra for all species assuming two-epoch, three-epoch or four-epoch demographic models were calculated. Assuming a gamma-distributed DFE, we repeated the DFE inference process under different demographic assumptions with the best-fit singleton-masking treatments.

Next, we examined the effect of singleton masking on our inference. We repeated the DFE inference process for each species using both unmasked and singleton-masked SYN-SFS and MIS-SFS, assuming a gamma-distributed DFE and the best-fit demographic models.

As estimates of mutation rates can be variable within species, we compiled alternative estimates of μ from the literature. The alternative estimates μ' were from pedigree-based or mutation accumulation lines (Text B). As μ is only utilized during the unscaling of DFE parameters (Text E), assuming the best-fit demographic model and singleton-masking treatment for each species, we re-estimated the long-term population size $N_a^{'\text{SYN}} = \frac{\theta_{\text{SYN}}}{4\mu' L_{\text{SYN}}}$ using the alternative mutation rates. Using the re-estimated $N_a^{'\text{SYN}}$, we repeated the unscaling for the gamma DFE's scale parameter from the population-scaled scale parameter β, to the individual-level scale parameter following $\beta'' = \beta / 2N_a^{'\text{SYN}}$.

We utilized a lineage-specific nonsynonymous to synonymous mutation rate ratio (r_L) with $r_L = 2.31$ for vertebrates and $r_L = 2.85$ for invertebrates in the main analyses presented in the text. To evaluate if lineage-specific r_L biased the DFE inference, we repeated the DFE inference using the same $r_L = 2.5$ across species, assuming a gamma-distributed DFE, and the best-fit demographic model and singleton-masking treatment for each species.

To assess the potential impact of GC-biased gene conversion and CpG hypermutability on DFE inference, we filtered the human and mouse datasets to retain only strong-to-strong and weak-to-weak mutations (SSWW mutations), following [5]. We used an SSWW mutation rate of 3.14×10^{-9} for humans and 5.99×10^{-10} for mice, along with a nonsynonymous-to-synonymous mutation rate ratio of 5.21, as estimated by [5]. Assuming a two-epoch demographic model, we repeated demographic inference for the full SYN-SFS (not masking singletons) in both humans and mice using only SSWW mutations. Conditioned on the inferred demographic parameters and assuming a gamma-distributed DFE, we then repeated DFE inference for the MIS-SFS derived from SSWW mutations.

A.7 Testing for phylogenetic signal

To evaluate whether the DFE co-varies with phylogeny, we calculated Pagel's λ using the `phylosig` function from the phytools package (v.2.4.4 [38]) in R. The log10 transformed mean selection coefficient ($\log_{10}(|s|_{\text{mean}})$), the log10 transformed mean selection coefficient at the population-level ($\log_{10}(|2N_a s|_{\text{mean}})$), the log10 transformed median selection coefficient ($\log_{10}(|s|_{\text{median}})$), and the shape parameter (α), of the gamma-distributed DFE, were considered candidate traits to test for phylogenetic

dependence. In addition, we calculated the Pagel's λ for life history traits and genomic attributes, including the generation time, mutation rate, body mass, age at maturity, maximum longevity, recombination rate, genome size, and the literature-based or synonymous-site-based long-term population size (Data S15). The underlying phylogenetic structure and divergence times for eleven (sub)species were compiled from the TimeTree database (http://timetree.org/) based on their scientific names. To control for outliers or account for the long branches between mammals and insects, we repeated the Pagel's λ analysis excluding the arctic wolves data point, or only within mammal data points.

To formally test whether the DFE differs across different species, we compared each species' DFE estimates to a null model where the DFE was constrained to be the same across species by likelihood-ratio tests (LRT). To do this, we employed a grid-search approach using the `DFE1D_gridsearch` module in the *varDFE* package.

To test for differences in s across species, we evenly spaced the same 2000 grid points in biologically meaningful ranges of shape ($\alpha = 0.1$ to 0.5) and scale ($\beta' = 0.0001$ to 0.5) parameters. We obtained the expected SFS for each $\alpha - \beta'$ pair for each species using the `integrate` method from reference spectra, and calculated the Poisson log-likelihood relative to the empirical MIS-SFS. From four million $\alpha - \beta'$ pairs, we explored the full Poisson log-likelihood surface for each species simultaneously. To obtain the null model where the DFE is constrained to be the same across species, we assumed that each species' SFS is independent of each other given their distant phylogenetic divergence. Therefore, the log-likelihood for the null model can be calculated by summing the log-likelihood at each $\alpha - \beta'$ pair for each species and the MLE can be found from within the four million $\alpha - \beta'$ parameter grids. To formally test whether the shape (α) and scale (β') are different in any x number of species, we used a likelihood ratio test [3]. The LRT was constructed as $\Lambda = -2 * ln(L_0/L_1) = -2 * (LL_0 - LL_1)$, where LL_0 is the log-likelihood for the null model, with two DFE parameters inferred ($\hat{\alpha_1} = \hat{\alpha_2} = ... = \hat{\alpha_x}, \hat{\beta}'_1 = \hat{\beta}'_2 = ... = \hat{\beta}'_x$; params = 2) given the inferred demographic parameters for each species ($\Theta_{D,1}, \Theta_{D,2}, ..., \Theta_{D,x}$), and LL_1 is the log-likelihood for the alternate model, where each species is allowed to have its own DFE parameters ($\hat{\alpha_1}, \hat{\alpha_2}, \hat{\alpha_x}, ..., \hat{\beta}'_1, \hat{\beta}'_2, \hat{\beta}'_x$; params = $2x$). Across the eleven (sub)species tested, asymptotically, Λ should follow a χ^2 distribution with $df = 2x - 2 = 20$.

$$\Lambda = -2 * ln[\frac{L(\alpha_1 = \alpha_2 \widehat{=} ... = \alpha_x, \beta'_1 = \beta'_2 \widehat{=} ... = \beta'_x | \Theta_{D,1}, \Theta_{D,2}, ..., \Theta_{D,x})}{L(\hat{\alpha_1}, \hat{\alpha_2}, ..., \hat{\alpha_x}, \hat{\beta}'_1, \hat{\beta}'_2, ..., \hat{\beta}'_x | \Theta_{D,1}, \Theta_{D,2}, ..., \Theta_{D,x})}] \quad (3)$$

However, as Λ is a composite likelihood, its empirical distribution under the null model of constrained DFE parameters is broader than the χ^2 distribution [3]. Although small P-values (e.g. $P < 10^{-16}$) should not be interpreted literally, simulations showed that Λ does not exceed 34 under the null model in a two-species comparison, suggesting that the probability of encountering a Λ substantially greater than 34 remains extremely low [3].

To demonstrate the phylogenetic correlations in the DFE evolution, we calculated the pairwise likelihood ratio test statistics in all possible pairs of species ($x = 2$). For example, the LRT statistics comparing humans (population 1) and *Drosophila* (population 2) DFE can be written as follows:

$$\Lambda = -2 * ln[\frac{L(\widehat{\alpha_1 = \alpha_2}, \widehat{\beta'_1 = \beta'_2} | \Theta_{D,1}, \Theta_{D,2})}{L(\hat{\alpha_1}, \hat{\alpha_2}, \hat{\beta}'_1, \hat{\beta}'_2 | \Theta_{D,1}, \Theta_{D,2})}]. \quad (4)$$

Asymptotically, Λ should follow a χ^2 distribution with $df = 2x - 2 = 2$. We hierarchically clustered the log10 scaled LRT statistics [$\log_{10}(\Lambda)$] using the `hclust` function in R.

To test the significance of the variation of the population-scaled DFE, $\gamma = 2N_a s$, for each species, we conducted the grid search by evenly spacing the same 2000 grid points in biologically meaningful ranges of shape ($\alpha = 0.1$ to 0.5) and population-scaled scale ($\beta = 100$ to 2.5×10^4) parameters for population-scaled DFE, and repeated the overall and pairwise LRT analyses as outlined above.

For the overall LRT analyses in population-scaled DFE, the LRT statistics for x populations can be written as follows:

$$\Lambda = -2 * ln[\frac{L(\alpha_1 = \widehat{\alpha_2 = ...} = \alpha_x, \beta_1 = \widehat{\beta_2 = ...} = \beta_x | \Theta_{D,1}, \Theta_{D,2}, ..., \Theta_{D,x})}{L(\hat{\alpha_1}, \hat{\alpha_2}, ..., \hat{\alpha_x}, \hat{\beta_1}, \hat{\beta_2}, ..., \hat{\beta_x} | \Theta_{D,1}, \Theta_{D,2}, ..., \Theta_{D,x})}]. \tag{5}$$

For the pairwise LRT analyses in population-scaled DFE, the LRT statistics for two populations (population 1 and 2) can be written as follows:

$$\Lambda = -2 * ln[\frac{L(\alpha_1 \widehat{=} \alpha_2, \beta_1 \widehat{=} \beta_2 | \Theta_{D,1}, \Theta_{D,2})}{L(\hat{\alpha_1}, \hat{\alpha_2}, \hat{\beta_1}, \hat{\beta_2} | \Theta_{D,1}, \Theta_{D,2})}]. \tag{6}$$

A.8 Correlating life history traits with the DFE variation across species (PGLS$_\lambda$)

To test if the candidate life history traits (X) are correlated with the observed $|s|_{\text{mean}}$ (Y), and correct for phylogenetic dependency when appropriate, we utilized the PGLS$_\lambda$ method (phylogenetic generalized least squares; [39]), implemented by the ape (v.5.8.1 [40]), nlme (v.3.1.166 [41]) and phytools packages in R. The PGLS$_\lambda$ method optimizes the phylogenetic signal of the residuals of $|s|_{\text{mean}}$ (λ) simultaneously with the regression parameters (function `phyl.resid` in phytools). Importantly, the λ estimated in PGLS$_\lambda$ does not equate to the Pagel's λ estimated individually for X or Y (described in the previous section), but represents whether the correlation of X and Y can be accounted for by phylogenetic dependence in residual error. Compared to the default PGLS model with a Brownian motion correlation structure ($\lambda = 1$), it allows for a more flexible variance-covariance matrix, therefore, improving model performance by applying an optimized degree of phylogenetic correction.

In our analysis, the candidate explanatory variables (X) included are:

1. Long-term population size from synonymous-site-based demographic inference (N_a^{SYN}) in diploids;
2. Long-term population size from the literature (N_a^{LIT}) in diploids;
3. Body mass in grams;
4. Generation time in years per generation;
5. Age at maturity in days;
6. Maximum longevity in years;
7. Recombination rate in recombination events per bp per generation;
8. Genome size in Mb;
9. Mutation rate in mutations per bp per generation.

The regression was specified as $\log_{10}(|s|_{\text{mean}}) \sim \log_{10}(X)$ to normalize the data, except when X was genome size, in which case X was not log-transformed. Given that the shape (α) parameter is often estimated more confidently, and independent of N_a and mutation rates during inference, we repeated PGLS$_\lambda$ analysis for α, with the regression specified as $\alpha \sim \log_{10}(X)$, except when X was genome size, in which case X was not log-transformed. Additionally, we repeated PGLS$_\lambda$ analysis for the log10 transformed median selection coefficient ($\log_{10}(|s|_{\text{median}})$), with the regression specified as

$\log_{10}(|s|_{\text{median}}) \sim \log_{10}(X)$, except when X was genome size, in which case X was not log-transformed.

Since parameters for the arctic wolf dataset reached upper boundaries during inference (Fig 1B, Data S6), we tested the impact on regression outputs when this data point was excluded (Data S16).

To account for the deep branches between mammals and insects, we repeated the PGLS_λ analysis described above only within mammals (n = 6).

We also repeated the PGLS_λ analysis for the log10 transformed mean selection coefficient at the population-level ($\log_{10}(|2N_a s|_{\text{mean}})$), with the regression specified as $\log_{10}(|2N_a s|_{\text{mean}}) \sim \log_{10}(X)$, except when X was genome size, in which case X was not log-transformed.

A.9 Fitting the FGM-derived DFE

To investigate the genetic mechanisms underlying DFE variation and to incorporate beneficial mutations in our inference, we implemented a functional form of the DFE which is directly derived from Fisher's geometric model [42]. Briefly, FGM proposes that organisms can be seen as a collection of phenotypes under stabilizing selection around a local maximum. A genotype generates a single phenotype, which is characterized by a point in an n-dimension phenotypic space. The phenotypic space's dimensionality, in other words, the total number of phenotypes under selection, is defined as complexity (n). Fitness (w) can be described as a Gaussian function of the distance to the optimum (z), $w(z) = exp(-z^2)$. Random mutations do not impact all phenotypes equally, but will likely affect a subset of m phenotypes with fitness effect size r. Here, m refers to mutation pleiotropy [42]. Effect size r follows a zero-mean Gaussian distribution with scale σ. When the population is under mutation-selection-drift balance, the increase in fitness from beneficial mutations should counteract the drift load, the decrease in fitness caused by deleterious mutations. The population will have an equilibrium phenotypic distance (z_{eq}) to the fitness optimum. Therefore, the DFE for the well-adapted population (equation 15 in [42]) can be described using the mutation pleiotropy (m), scale of mutation effects (σ) and long-term population size (N_a^{FGM}), $s \sim \textit{Lourenco}(m, \sigma, N_a^{\text{FGM}})$. Here $\Gamma(.)$ is the gamma function and $K(.)$ is the modified Bessel function of the second kind [42]. We note that there is a missing exponential term in equation 15 in [42], which is corrected in the equation below:

$$f(s) = \frac{2^{\frac{1-m}{2}}\sqrt{N_a^{\text{FGM}}}(|s|)^{\frac{m-1}{2}}(1+\frac{1}{N_a^{\text{FGM}}\sigma^2})^{\frac{1-m}{4}}exp(-N_a^{\text{FGM}}s)}{\sqrt{\pi}\sigma^m\Gamma(\frac{m}{2})} \times K_{\frac{m-1}{2}}(N_a^{\text{FGM}}|s|\sqrt{1+\frac{1}{N_a^{\text{FGM}}\sigma^2}}). \quad (7)$$

While complex in its expression, this FGM-derived distribution is functionally equivalent to the previously described gamma and lognormal distributions for generating mutational effects. Therefore, we estimated its parameters (m, σ, and N_a^{FGM}) following methods described in the DFE inference section, through maximum-likelihood optimization by fitting the observed differences between MIS-SFS and SYN-SFS across minor allele frequency bins. This FGM-derived distribution is for individual-level selection coefficient (s), and $\partial a \partial i$ performs parameter optimization on population-level selection coefficient (γ). To obtain s during parameter optimization, we divided γ by $2N_a^{\text{SYN}}$ from demographic estimates. And N_a^{FGM} in $f(s)$ was kept as a free parameter to be optimized. We inferred the maximum likelihood estimates of the m, σ, and N_a^{FGM} parameters using the `DFE1D_inferenceFIM` module as described in the DFE inference section. Since this DFE considers beneficial mutations, we calculated the expected SFS using the `integrate_continuous_pos` method implemented in our *varDFE* package. The

`integrate_continuous_pos` method was implemented following equation 3 in [35]. It was implemented based on the `integrate` function in $\partial a \partial i$, and extends integration from $-\infty < \gamma \leq 0$ to $-\infty < \gamma < +\infty$. To compute the proportion of mutations with different values of s in the inferred FGM-derived DFE, we found the cumulative probability for s ranged in [-0.5, -0.01), [-0.01, -0.001), [-0.001, -0.0001), [-0.0001, -10^{-5}), [-10^{-5}, 0), [0, 10^{-5}), [10^{-5}, 0.0001), and [0.0001, 0.5] using the `scipy.integrate.quad` function (v.1.8.0) in Python. The proportion of beneficial mutations was obtained by calculating the cumulative probability for $s > 0$.

To compare the relationship between N_a inferred in this FGM-derived DFE with the N_a obtained in demographic inference, we performed a PGLS$_\lambda$ analysis on $\log_{10}(N_a^{\text{FGM}}) \sim \log_{10}(N_a^{\text{SYN}})$ using the methods described above. A PGLS$_\lambda$ analysis between the proportion of beneficial mutations and literature-based N_a^{LIT} ($P(s > 0) \sim \log_{10}(N_a^{\text{LIT}})$) was also performed using the same method. To test for phylogenetic signal in the inferred DFE parameters, we calculated Pagel's λ for m and σ using methods described above (Data S15). We also performed a PGLS$_\lambda$ analysis between the inferred m and σ parameters in this FGM-derived DFE function.

We note that beneficial mutations are more likely to be fixed as substitutions rather than segregating in the site-frequency spectrum. Therefore, although the methods described here can infer proportions of beneficial mutations, substitution data between closely-related species are preferred over the SFS to more reliably estimate the proportion of beneficial mutations [5]. Additionally, when the population is not under mutation-selection-drift equilibrium, its DFE has been derived as equation 8 in [42]. Specifically, the distance from optimum z is no longer determined by population size. Further, z and n become additional free parameters to be inferred. We did not include this model here given that it did not provide a better fit than the equilibrium DFE in [3].

A.10 Simulating human DFE with Drosophila demography

To investigate whether Fit$\partial a \partial i$ can accurately detect strongly deleterious mutations in species with large population sizes, we performed a simulation study using *Drosophila* demography and the human DFE. We used the stdpopsim library to obtain coding sequence (CDS) annotations for *Drosophila melanogaster* chromosomes 2L, 2R, 3L, and 3R from the FlyBase_BDGP6.32.51_CDS [43]. CDS regions were labeled as "exon", and non-coding regions as "bkgd". To simulate realistic genomic regions, we randomly sampled 108 1-Mb segments from these chromosomes with probabilities proportional to chromosome lengths. Annotations for each segment were subset and converted to SLiM's coordinate format. Next, we implemented a two-epoch *Drosophila* demographic model in which the ancestral population size was set to 2,766,461, and the population expanded to 7,482,090 over 507,689 generations. We used a uniform recombination rate of 2.03806×10^{-8} which is an average of recombination rates for *Drosophila* chromosomes from the stdpopsim catalog. The mutation rate was set to 1.5×10^{-9} per bp per generation. Deleterious mutations followed a human-like gamma DFE (mean = –0.0276, shape = 0.189) with a nonsynonymous-to-synonymous ratio of 2.85. Background (noncoding) regions were assigned neutral mutations only. All mutations were additive (semi-dominant). From each simulation run on the randomly sampled 1-Mb *Drosophila*chromosome segments, we output SFS using a sample size of 100 or a sample size of 10. To reduce computational cost, we applied a scaling factor Q = 100, adjusting population size, mutation rate, recombination rate, and selection coefficients accordingly. One out of 108 simulations did not complete due to time constraints on the cluster and was discarded. To match the total length of chromosomes 2L, 2R, 3L, and 3R in *Drosophila*, we sampled the SFS outputs from 107 successful

simulations with replacement, and summed 107 SFS outputs to generate one bootstrapped SFS that mimics a resampled version of the simulated *Drosophila* genome ($\sim$ 107 Mb). To assess inference uncertainty, we repeated the sampling 50 times, producing 50 bootstrapped SFSs for each sample size (2n = 100 or 2n = 10). We then inferred the DFE on the 50 bootstrapped SFSs as done for the empirical data. We assumed either a gamma-distributed or a lognormal-distributed DFE during inference. To evaluate the influence of selection at linked sites on synonymous mutations, we also inferred demographic parameters from the "bkgd" (non-coding) regions to compare with the demographic parameters inferred from synonymous mutations.

B Text B: Source of mutation rates

Scientific Name	Based on	Mutation Rate	Estimation Method	Citation
**Original mutation rate**				
Anopheles coluzzii	*Drosophila melanogaster*	1.50×10^{-9}	Divergence-based	[5]
Drosophila melanogaster	*Drosophila melanogaster*	1.50×10^{-9}	Divergence-based	[5]
Lasioglossum calceatum	*Drosophila melanogaster*	1.50×10^{-9}	Divergence-based	[5]
Ficedula hypoleuca	*Ficedula*	2.80×10^{-9}	Divergence-based	[16]
Ficedula albicollis	*Ficedula*	2.80×10^{-9}	Divergence-based	[16]
Canis lupus lupus	*Canis familiaris*	5.62×10^{-9}	Divergence-based	[17]
Canis lupus arctos	*Canis familiaris*	5.62×10^{-9}	Divergence-based	[17]
Phocoena sinus	*Phocoena sinus*	5.83×10^{-9}	Divergence-based	[4]
Balaenoptera physalus	*Balaenoptera acutorostrata*	2.77×10^{-8}	Divergence-based	[44]
Mus musculus castaneus	*Mus musculus*	5.40×10^{-9}	Mutation Accumulation Line	[5]
Homo sapiens	*Homo sapiens*	2.50×10^{-8}	Divergence-based	[3]
**Alternative mutation rate**				
Anopheles coluzzii	*Drosophila melanogaster*	3.50×10^{-9}	Mutation Accumulation Line	[45]
Drosophila melanogaster	*Drosophila melanogaster*	5.49×10^{-9}	Mutation Accumulation Line	[46]
Lasioglossum calceatum	*Apis mellifera*	3.40×10^{-9}	Parent-progeny	[47]
Ficedula hypoleuca	*Ficedula albicollis*	4.60×10^{-9}	Pedigree-based	[48]
Ficedula albicollis	*Ficedula albicollis*	4.60×10^{-9}	Pedigree-based	[48]
Canis lupus lupus	*Canis familiaris*	4.00×10^{-9}	Tip-dating	[49]
Canis lupus arctos	*Canis familiaris*	4.00×10^{-9}	Tip-dating	[49]
Phocoena sinus	*Phocoena sinus*	5.83×10^{-9}	No alternative	[4]
Balaenoptera physalus	*Mysticeti*	1.39×10^{-8}	Divergence-based	[50]
Mus musculus castaneus	*Mus musculus*	3.90×10^{-9}	Pedigree-based	[51]
Homo sapiens	*Homo sapiens*	1.29×10^{-8}	Composite-likelihood based on IBD	[52]

C Text C: Estimating the proportion of lethal mutations using the gammalet DFE

We attempted to directly estimate the proportion of effectively lethal mutations ($s = -0.5$) by assuming that the DFE follows a mixture of a gamma distribution with additional lethal point mass (gammalet DFE [53], Data S8).

Specifically, the gammalet DFE is constructed by adding a point mass at lethality ($0 \leq p_{\text{let}} < 0.5$) to the original gamma DFE distribution (Text A). $|s|_{\text{mean}}$ was not calculated for this distribution, given the presence of lethal mutations.

Only three datasets (mosquitoes, arctic wolves, vaquitas) had a lower AIC score for the gammalet DFE compared with the gamma DFE (Figs I, K). In these datasets, the proportion of lethal mutations reached unrealistic high values (31% in vaquitas to 50% in mosquitoes, Fig J).

Given that lethal mutations are more likely to be recessive, it challenges Fit$\partial a \partial i$'s underlying assumption of additive fitness effects for all mutations. Therefore, we suspected that our current approach lacks the power to confidently infer the proportion of lethal mutations [35, 53].

D Text D: DFE inference is robust across different technical treatments

We evaluated the sensitivity of our DFE inferences to the choice of demographic model, singleton-masking treatments, mutation rate, the nonsynonymous to synonymous mutation rate ratio (r_L), and GC-biased gene conversion.

We tested the potential impacts of demographic model misspecification, given that different demographic models were selected in each dataset. Assuming the DFE follows a gamma distribution, we repeated the DFE inference for each dataset using the demographic parameters inferred from two-, three-, and four-epoch models (Data S10). Notably, in some datasets, unreasonable demographic model assumptions could lead to failed DFE inference, with the difference between the log-likelihoods of data and model (ΔLL) exceeding 200 units, such as two-epoch models in the mosquitoes and collared flycatchers datasets (translucent points in Fig P(A)). Overall, the average mutation effects and proportion of each mutation category remain unchanged qualitatively (Figs P(A), Q). However, when assuming three-epoch or four-epoch model for the mouse dataset, $|s|_{\text{mean}}$ is reduced from 0.010 (two-epoch) to 0.00089 (three-epoch) or 0.0014 (four-epoch). Nevertheless, the two-epoch based $|s|_{\text{mean}}$ estimate is more appropriate for the mouse dataset, because there is minimal improvement in log-likelihood and visual fit for the three- and four-epoch models (Fig C), and the estimated parameter values in these more complex models reflect extreme demographic events (Data S4).

Next, we tested whether masking singletons in the SFS during demography and DFE inference altered the inference of $|s|_{\text{mean}}$ (Data S11). Again, the average mutation effects and proportion of each mutation category are largely qualitatively unchanged (Figs P(B), R), except that $|s|_{\text{mean}}$ is reduced from 0.013 (not masking singletons) to 0.0023 (masking) in the vaquita dataset.

As estimates of mutation rates can be variable within species, we compiled alternative estimates of μ from the literature. The original estimates of μ used in the main text were derived primarily from divergence-based estimates, and the alternative estimates μ' were from pedigree-based or mutation accumulation lines (Text B). As μ is only utilized during the unscaling of DFE parameters (Text E), we re-estimated the long-term population size $N_a' = \frac{\theta_{\text{SYN}}}{4\mu' L_{\text{SYN}}}$ using the alternative mutation rates. Using the re-estimated N_a', we repeated the unscaling for the gamma DFE's scale parameter from the population-scaled scale parameter β, to the individual-level scale parameter following $\beta'' = \beta/2N_a'$. Overall, mutations become more deleterious in insects and birds, given that the original mutation rates are lower than the alternative mutation rates in insects and birds (Fig S). However, the ranking of $|s|_{\text{mean}}$ estimates remains unchanged, with mutations being 2.1 to 956 times more deleterious in mammals compared to insects and birds (Pagel's $\lambda = 0.70$, P = 0.09). The mean selection coefficient, $|s|_{\text{mean}}$ is more deleterious in mammals ($|s|_{\text{mean}} = 0.007$ in humans to 0.47 in arctic wolves) compared to insects ($|s|_{\text{mean}} =$ 0.00050 in *Drosophila* to 0.0013 in mosquitoes) and birds ($|s|_{\text{mean}} = 0.00084$ in pied flycatchers and 0.0034 in collared flycatchers).

We utilized a lineage-specific nonsynonymous to synonymous mutation rate ratio (r_L) with $r_L = 2.31$ for vertebrates and $r_L = 2.85$ for invertebrates in the main analyses presented in the text. To evaluate if lineage-specific r_L biased the DFE inference, we repeated the DFE inference using the same $r_L = 2.5$ across species, assuming a gamma-distributed DFE, and the best-fit demographic model and singleton-masking treatments. Overall, mutations become less deleterious in insects, and more deleterious in birds and mammals, given that the original r_L is higher than the alternative r_L in insects (Fig T). The ranking of $|s|_{\text{mean}}$ estimates becomes more distinctive, with mutations being 4.7 to 7309 times more deleterious in mammals compared to insects and birds (Pagel's $\lambda = 0.90$, P = 0.003). The mean selection coefficient,

$|s|_{\text{mean}}$ is more deleterious in mammals ($|s|_{\text{mean}} = 0.014$ in mice to 0.72 in arctic wolves) compared to insects ($|s|_{\text{mean}} = 9.9 \times 10^{-5}$ in *Drosophila* to 0.00039 in mosquitoes) and birds ($|s|_{\text{mean}} = 0.00067$ in pied flycatchers and 0.0029 in collared flycatchers).

To assess the potential impact of GC-biased gene conversion and CpG hypermutability on DFE inference, we filtered the human and mouse datasets to retain only strong-to-strong and weak-to-weak mutations (SSWW mutations), following [5]. We used a SSWW mutation rate of 3.14×10^{-9} for humans and 5.99×10^{-10} for mice, along with a nonsynonymous-to-synonymous mutation rate ratio of 5.21, as estimated by [5]. Assuming a two-epoch demographic model, we repeated demographic inference for the SYN-SFS in both humans and mice using only SSWW mutations. Conditional on the inferred demographic parameters and assuming a gamma-distributed DFE, we then repeated DFE inference for the MIS-SFS derived from SSWW mutations. In humans, the inferred DFE was largely unchanged when restricted to SSWW mutations. The gamma shape parameter was 0.17 for the SSWW-restricted analysis versus 0.19 for the full SFS, and the scale parameter was 0.073 versus 0.10, respectively (Fig U). In mice, the DFE shifted modestly for SSWW mutations. The gamma shape parameter remained unchanged (0.21 in both analyses), whereas the scale parameter decreased from 0.048 in the full SFS to 0.029 in the SSWW-restricted analysis. This corresponded to a reduction in the inferred proportion of strongly deleterious mutations ($s > 0.1$), from 24% to 17%. Overall, the effects of restricting analyses to SSWW mutations were small relative to the DFE variation across species (Fig U), suggesting that GC-biased gene conversion and CpG hypermutability have limited influence on DFE inference in these datasets. However, broader evaluation across additional vertebrate species will be required to assess the generality of this pattern.

In summary, our examination of different functional forms of the DFE, demographic models, singleton masking strategies, different mutation rates, different nonsynonymous to synonymous mutation rate ratios (r_L), and GC-biased gene conversion, suggests that the DFE inference is robust to choices made during the analysis.

E Text E: The dependence of s on μ, r_L and N_a

In $\partial a \partial i$ and most SFS-based DFE inference methods, DFE estimates are obtained for the population-scaled selection coefficient γ. To obtain the distribution of individual-level selection coefficient s, several variables are involved to "unscale" γ. Here, assuming a gamma-distributed DFE, we give a methodological summary on how $|s|_{\text{mean}}$ depends on mutation rate μ, nonsynonymous to synonymous mutation rate ratio r_L, and long-term (i.e., ancestral) population size N_a.

During demographic inference, the population-scaled mutation rate in synonymous regions is inferred from the SYN-SFS as θ_{SYN}. The population-scaled mutation rate in nonsynonymous regions is obtained by $\theta_{\text{MIS}} = r_L \theta_{\text{SYN}}$. The shape α and scale β parameters of the gamma-distributed DFE for the population-scaled selection coefficient γ are inferred from the MIS-SFS given θ_{MIS}. Long-term (ancestral) population size from demographic inference is obtained by $N_a^{\text{SYN}} = \frac{\theta_{\text{SYN}}}{4\mu L_{\text{SYN}}}$. Throughout the inference process, μ is not involved in the population-scaled DFE parameter inference, but is used for N_a^{SYN} calculations. r_L influences population-scaled DFE parameter inference by changing the log-likelihood surface through θ_{MIS}.

To obtain individual-level selection coefficient s, the shape α parameter does not need to be unscaled, and is not influenced by different values of μ or N_a^{SYN}. However, different r_L changes θ_{MIS}, which could result in different α estimate. To obtain β', which is the individual-level scale parameter in the gamma-distributed DFE, the population-scaled scale parameter β is unscaled using the equation $\beta' = \beta / 2N_a^{\text{SYN}}$. The mean of the gamma-distributed DFE can be calculated using $|s|_{\text{mean}} = \alpha\beta' = \frac{\alpha\beta}{2N_a^{\text{SYN}}} = \frac{|\gamma|_{\text{mean}}}{2N_a^{\text{SYN}}}$. Therefore, regressing $|s|_{\text{mean}}$ against N_a^{SYN} could result in Pearson's spurious correlation of ratios. Similarly, because $N_a = \frac{\theta_{\text{SYN}}}{4\mu L_{\text{SYN}}}$, $|s|_{\text{mean}} = \frac{2\mu L_{\text{SYN}}|\gamma|_{\text{mean}}}{\theta_{\text{SYN}}}$, regressing $|s|_{\text{mean}}$ against μ could also result in Pearson's spurious correlation of ratios.

To attempt to account for N_a^{SYN} appearing in both the dependent and independent variables in the PGLS$_\lambda$ analysis, we used literature-based N_a^{LIT} instead of synonymous-site-based N_a^{SYN} as the independent variable in the PGLS$_\lambda$ analysis. In addition, we found a strong concordance between these independent N_a^{LIT} and N_a^{SYN} estimates (Fig E). While the potential for spurious correlations cannot be entirely eliminated between $|s|_{\text{mean}}$ and N_a, the fact that estimates of N_a^{LIT} not used to convert γ to s show a correlation with s, suggests that this correlation is not merely an artefact of the inference. Since α and $|2N_a s|_{\text{mean}}$ are calculated without inputs of N_a or μ, they are less likely to show spurious correlations with N_a or μ. The shape parameter α is significantly correlated with N_a^{LIT} (Fig 3D), whereas no correlation was found between $|2N_a s|_{\text{mean}}$ and N_a^{LIT} (Fig AH).

Overall, it is challenging to establish a clear causal relationship between long-term population size and individual-level selection coefficients s. Future studies are needed to develop methods to estimate s without inputs of long-term population sizes.

F Text F: Measurements of organismal complexity

Understanding organismal complexity has been central to biology, yet no single definition has achieved consensus. Here we adopted a pragmatic assumption common in comparative biology: mammals are generally considered more complex than insects, with birds being intermediate. To evaluate the molecular and cellular basis of this claim, we consider several independent proxies for complexity, particularly those derived from molecular phenotypes.

Historically, biological complexity was often associated with the C-value, the amount of DNA in the haploid genome. In our dataset, mutations appear more deleterious in species with larger genomes (Fig 3C). Mammals have larger genomes than birds, whose genomes are larger than those of insects. However, the C-value paradox highlights that genome size does not consistently correlate with organismal complexity across taxa [54].

Single-cell genomics now provides more direct estimates of organismal complexity through the number of unique cell types. Recent single-cell transcriptomic surveys report more than 475 human cell types [55], roughly 157 in chicken [56], and over 250 in Drosophila [57]. A meta-analysis of 227 species also found that vertebrates have more unique cell types than invertebrates [58]. These estimates support more diverse cell types in humans, but also suggest that the number of cell types varies less dramatically among animals than often assumed. The lower count in birds may partly reflect incomplete sampling rather than reduced complexity.

Complexity is also reflected in the diversity of proteins generated from a finite set of genes. Alternative splicing is the primary mechanism for this diversification. A comparative analysis across the tree of life shows that mammals and birds exhibit the highest levels of alternative splicing, whereas arthropods display intermediate levels, higher than unicellular eukaryotes but lower than vertebrates [59]. This pattern is consistent with the drift-barrier hypothesis: species with large effective population sizes, such as many insects, may maintain lower alternative splicing rates because purifying selection more efficiently optimizes splice signals [60].

Network architecture provides another perspective on complexity. Protein–protein interaction (PPI) and transcriptional regulatory networks capture how molecular information is integrated within cells. The human reference interactome HuRI contains approximately 53,000 binary protein interactions [61], whereas the Drosophila reference interactome DroRI reports 17,232 interactions among 6,511 proteins [62]. Since interactome maps are generated using different experimental strategies, direct quantitative comparisons across species remain challenging.

738 # Supplementary Figures

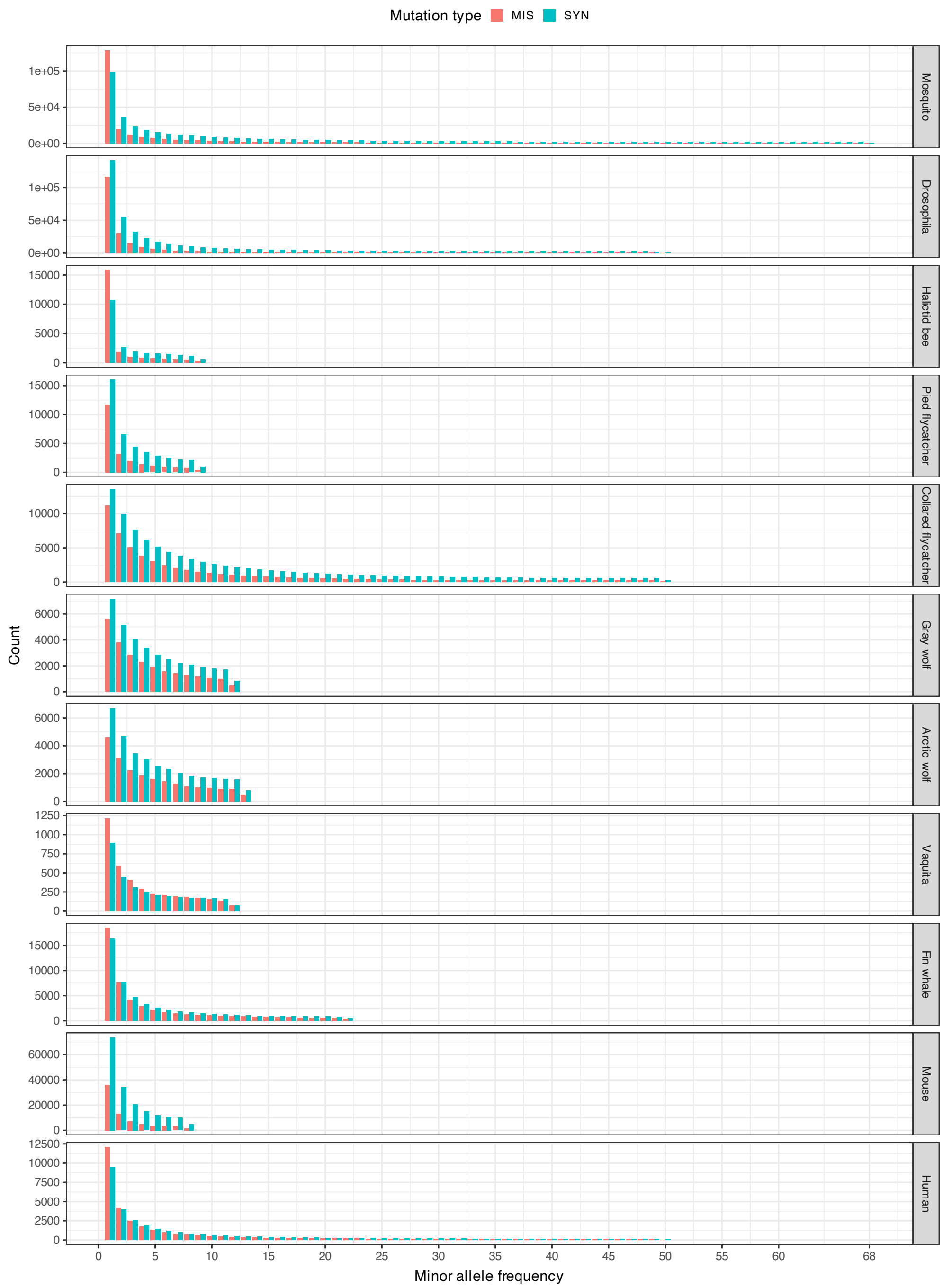

Figure A: The projected folded SFS for the eleven (sub)species datasets. The red bars represent the nonsynonymous/missense SFS while the cyan bars show the synonymous SFS. Dataset names are marked at the right.

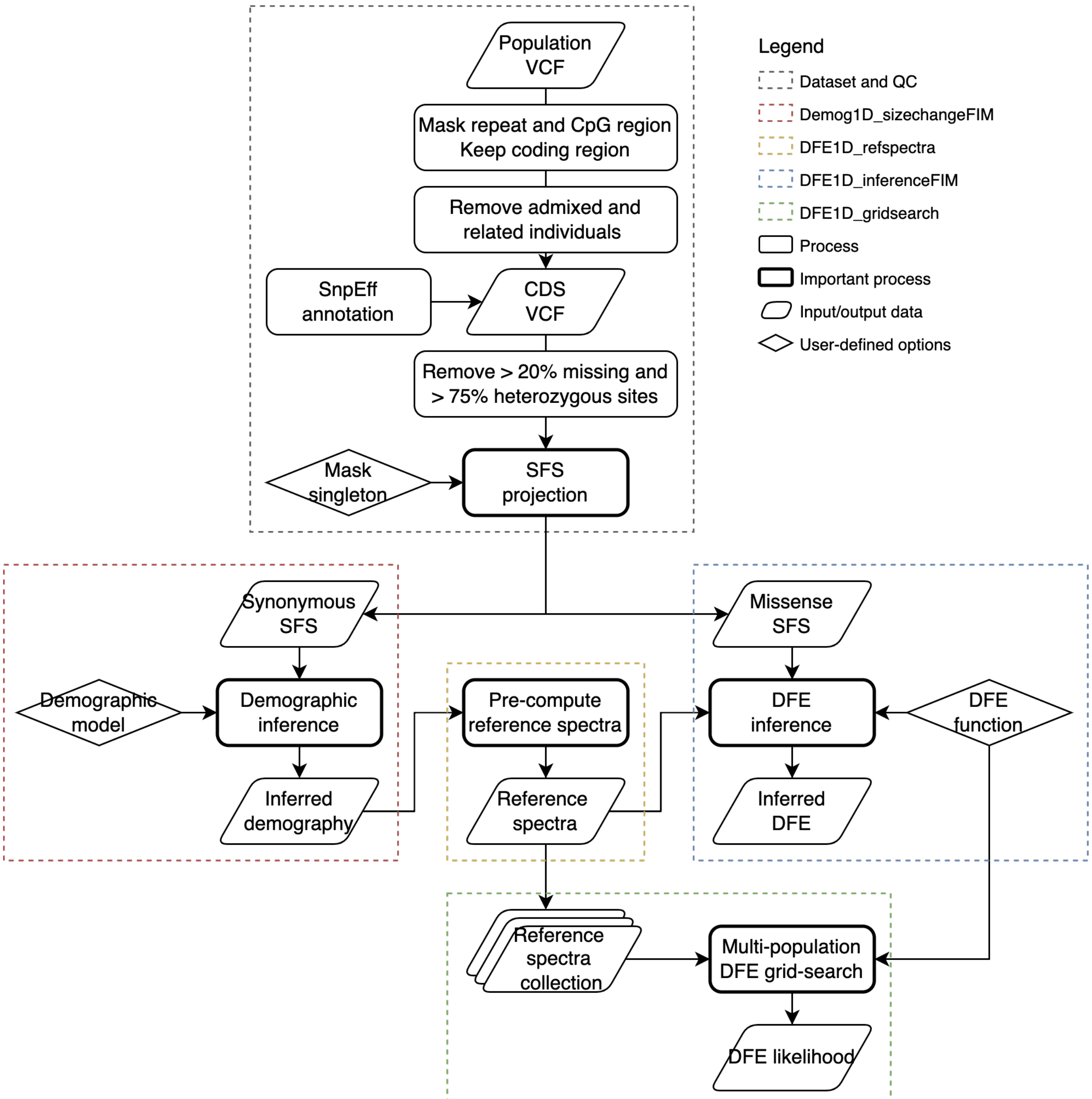

Figure B: The *varDFE* workflow. Each colored dashed rectangle represents a module in *varDFE*. The gray dashed rectangle shows the quality control and preprocessing steps for the eleven (sub)species datasets that are not part of the *varDFE* package.

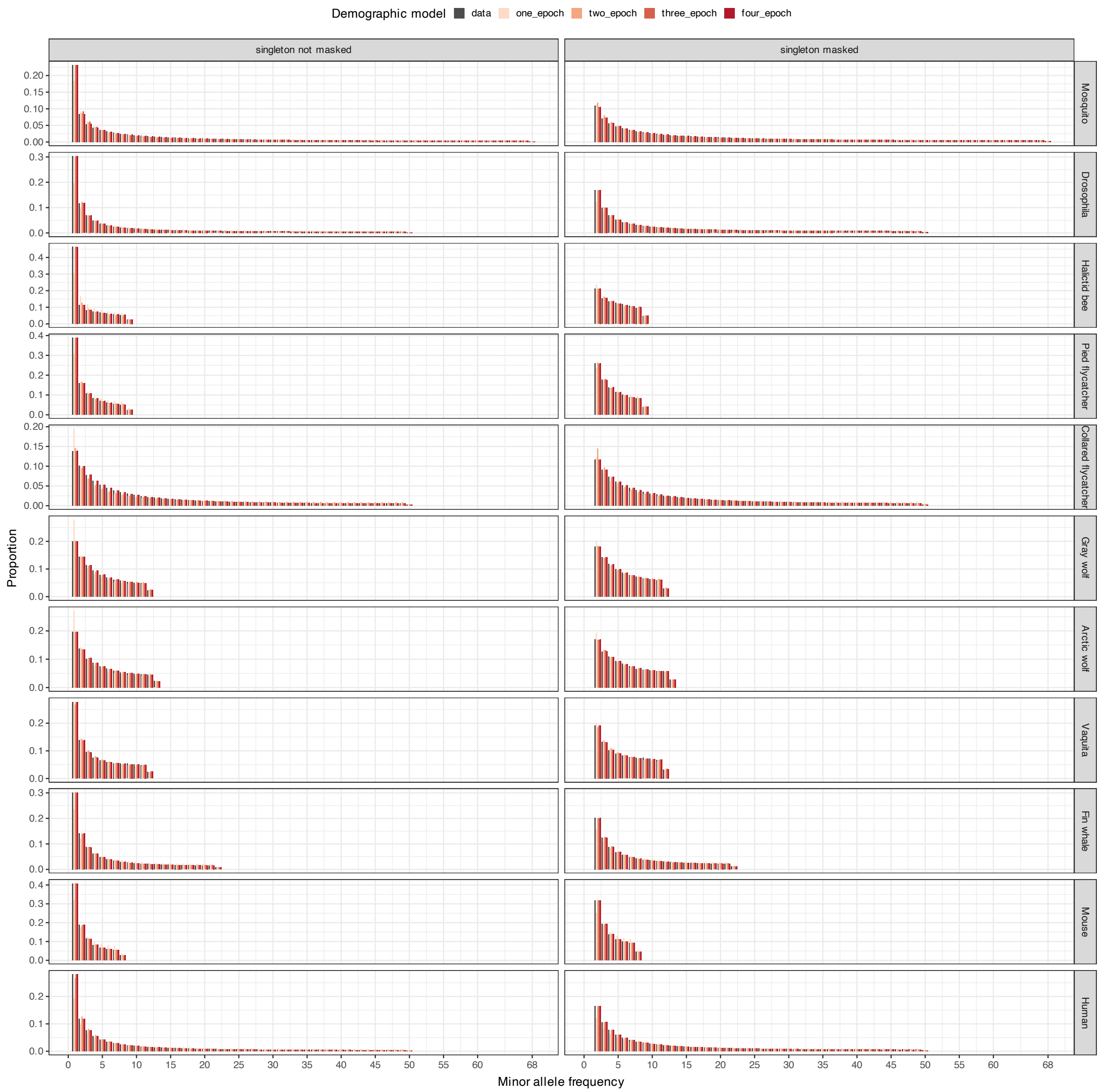

Figure C: Comparison of the observed synonymous SFS (SYN-SFS) to the predicted SFS under different demographic models. For each species (each row), the demographic inference results using the full SFS (left column) or singleton-masked SFS (right column) are shown. Within each panel, the proportional SYN-SFS data is plotted as the left-most bar, and the maximum-likelihood estimate's expected SFS for the standard neutral model (one-epoch), two-epoch, three-epoch and four-epoch models are shown to the right of the data. Note the overall fit of the two-epoch model using the full SFS and the improved fit of respective chosen models for the mosquitoes, halictid bees, gray wolves, and collared flycatchers datasets.

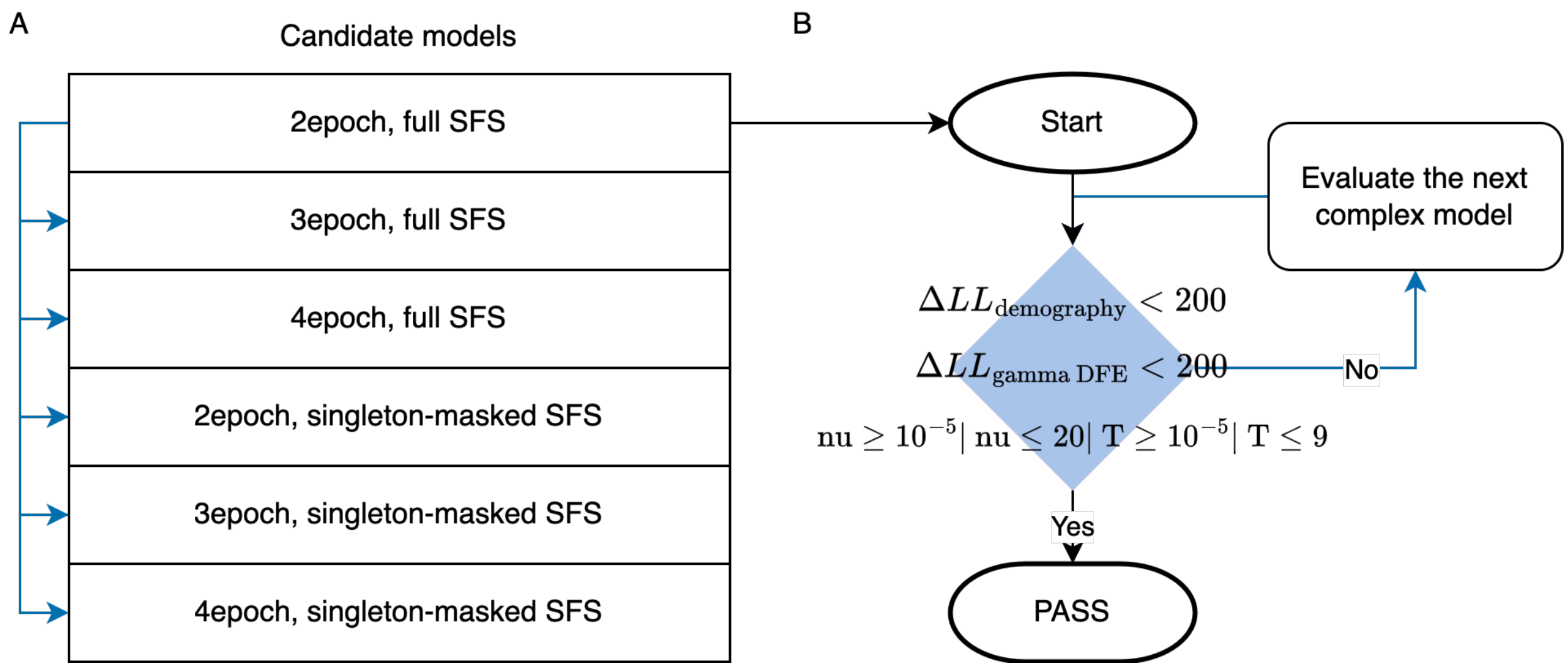

Figure D: The step-wise demographic model selection procedure. (A) To maximize the data available and use the most parsimonious model whenever possible, at the start of model evaluation, the two-epoch demographic model based on the unmasked SYN-SFS run was chosen for all datasets (top row). (B) This "2epoch, full SFS" run was chosen for a dataset (black workflow lines), if it met all three criteria listed in the blue diamond. 1. The log-likelihood differences between the data and model maximum likelihood $\Delta LL = LL_{\text{data}} - LL_{\text{model}}$ for demographic inference is less than 200. 2. The ΔLL for gamma DFE inference based on the current demographic model and singleton-masking treatment is less than 200. 3. The parameter estimates in the current demographic model are realistic (unrealistic parameters defined as nu $< 10^{-5}$ or nu > 20 or T $< 10^{-5}$ or T > 9). (A-B)Otherwise, we proceeded to evaluate the more complex run "3epoch, full SFS" with the same criteria (Blue workflow lines). If the "3epoch, full SFS" failed the criteria again, we proceeded to choose the next model listed in (A) iteratively. If all models based on the full (unmasked) SFS failed to provide a good fit (rows 1-3 in A), we proceeded to mask the singletons in the SFS (rows 4-6 in A).

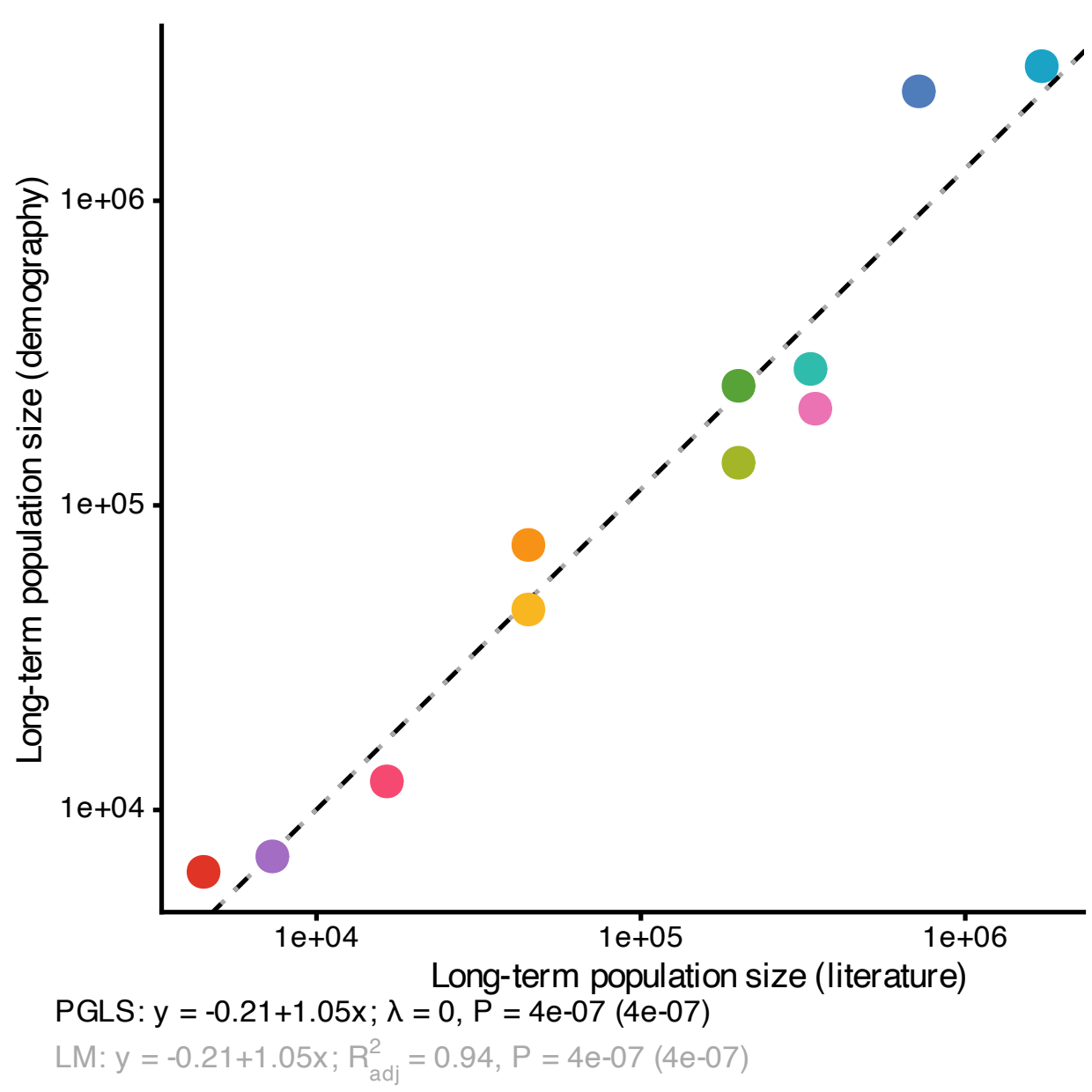

Figure E: Correlation between estimates of the long-term population size based on demographic inference from synonymous variants (demography) and estimates from the literature.

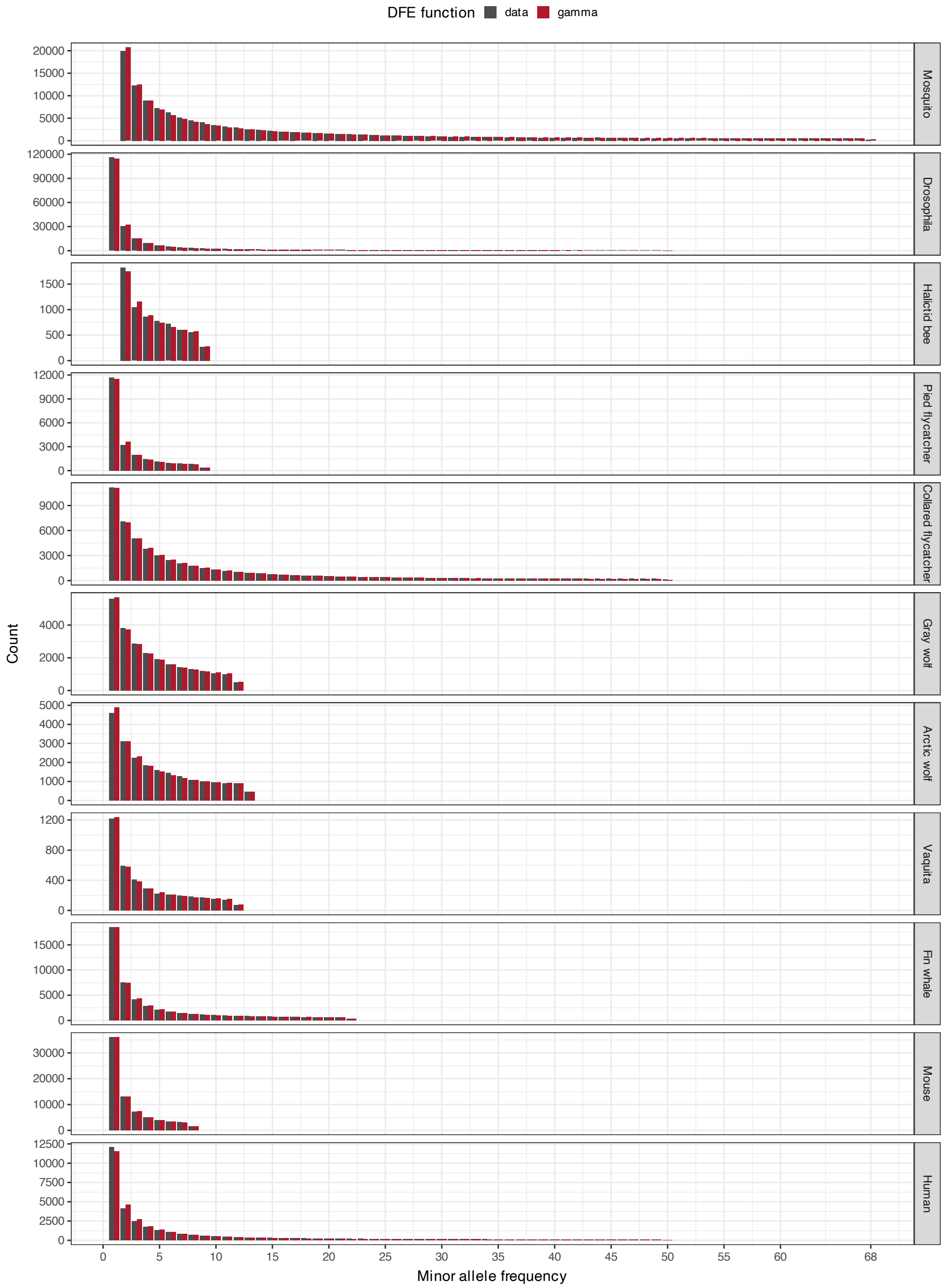

Figure F: Comparison of the observed nonsynonymous SFS (MIS-SFS) to the predicted SFS assuming a gamma DFE. The MIS-SFS data (gray) and expected SFS at the maximum-likelihood parameter estimates (red) are shown for each dataset.

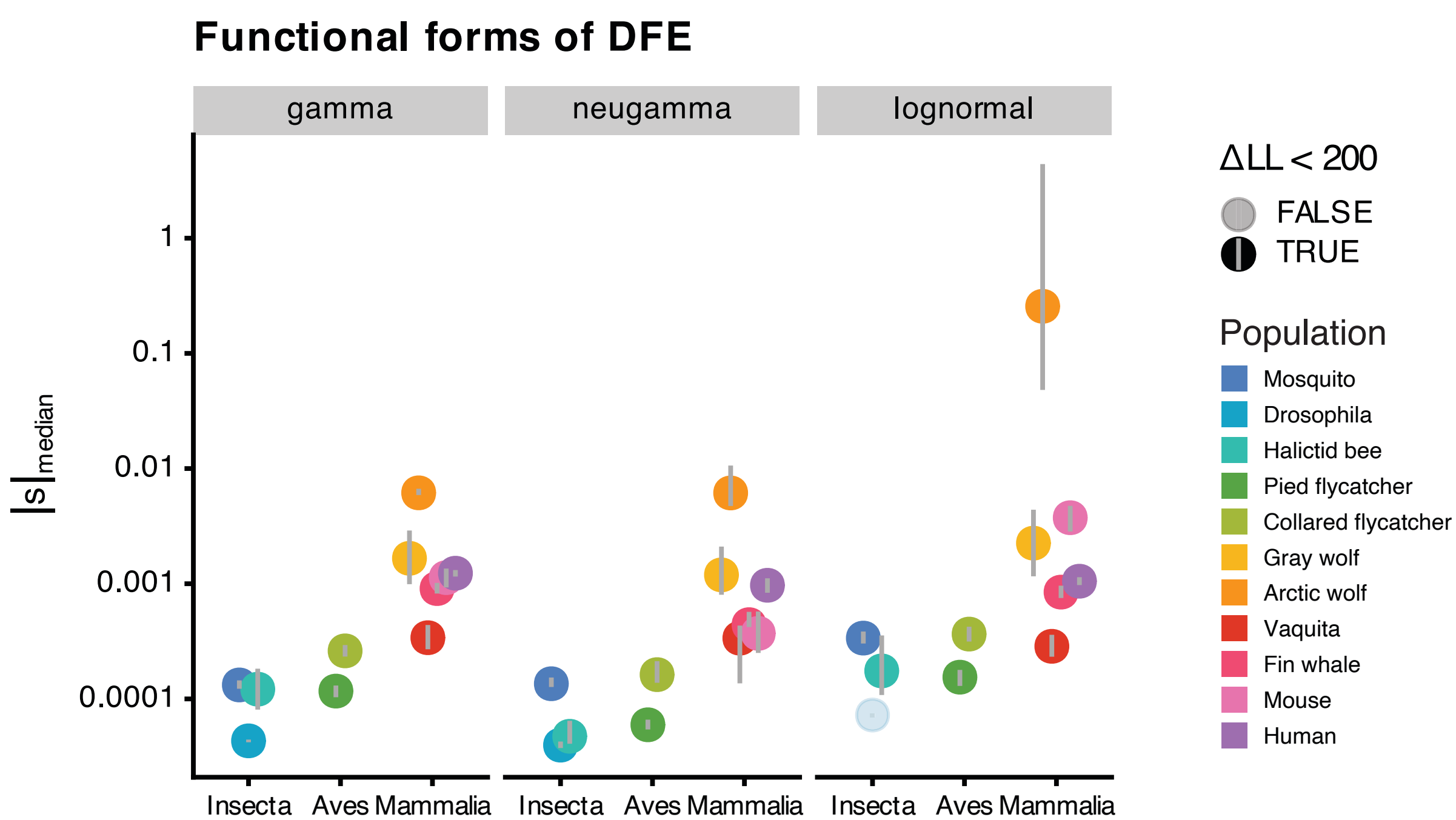

Figure G: The DFE inference is mostly robust to potential confounding factors. The median value of the selection coefficient for each species under different functional forms of the DFE. Points with different colors represent different (sub)species. In DFE inferences that did not fit the data, defined as the log-likelihood difference between the data and model exceeding 200 ($\Delta LL < 200$ is FALSE), points are marked as translucent.

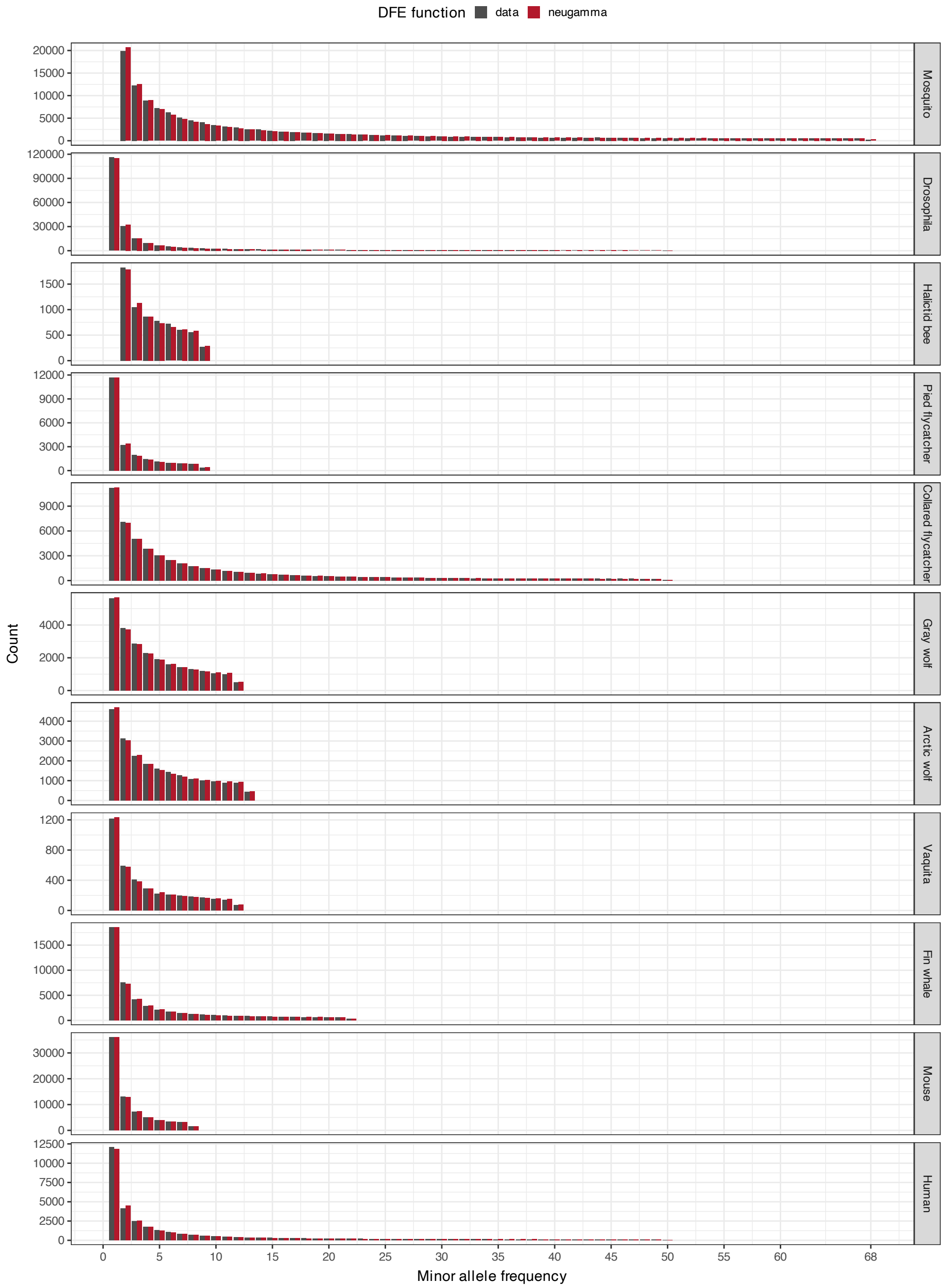

Figure H: Comparison of the observed nonsynonymous SFS (MIS-SFS) to the predicted SFS assuming the DFE is a mixture of a gamma distribution and a neutral point mass (neugamma DFE). The MIS-SFS data (gray) and expected SFS at the maximum-likelihood parameter estimates (red) are shown for each dataset.

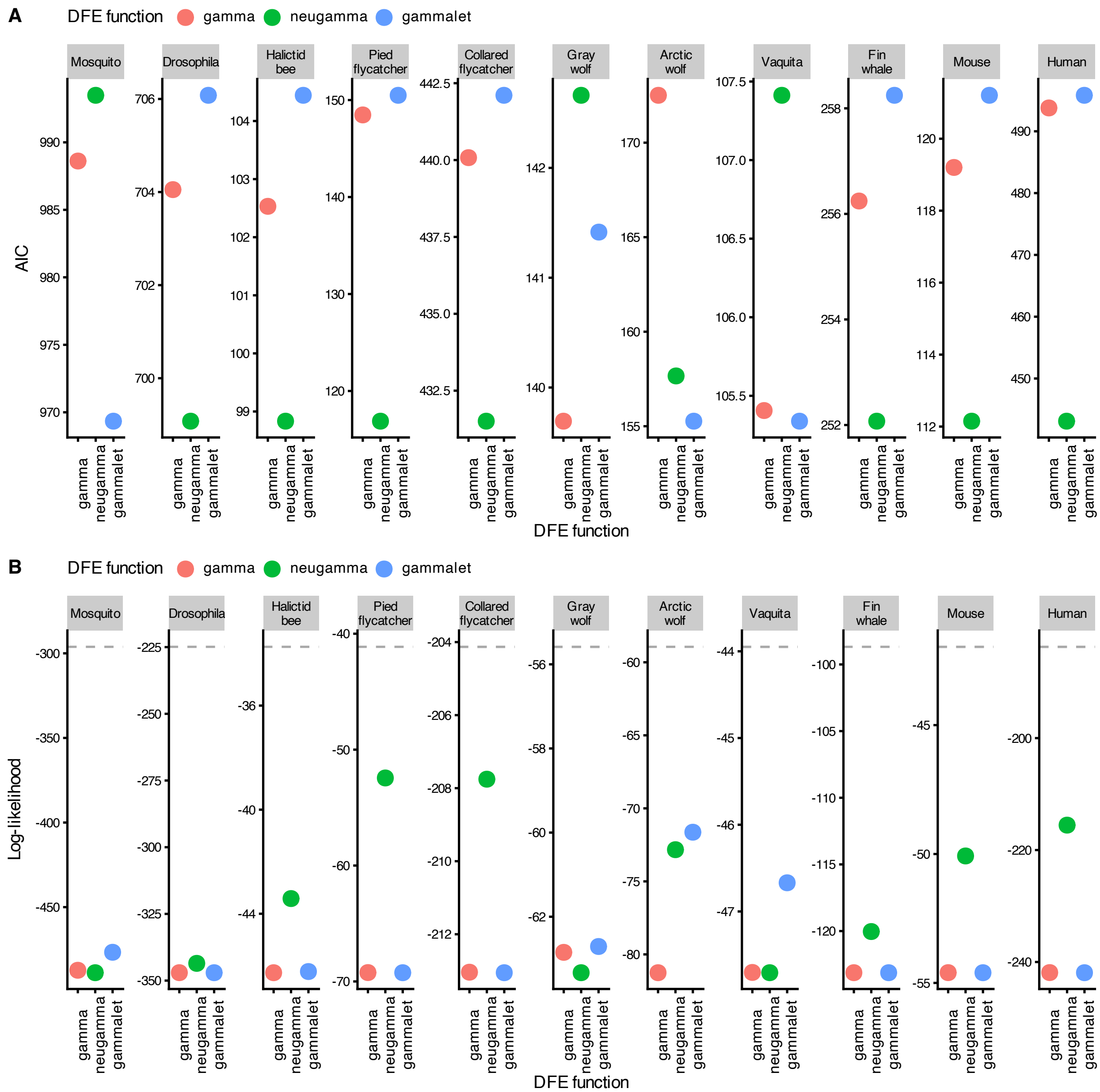

Figure I: Comparisons of the (A) AIC, and (B) log-likelihoods of the DFE inference assuming that the DFE follows a gamma, neugamma, or gammalet distribution. The gray dashed line in (B) represents the maximum log-likelihood derived from the data compared to itself.

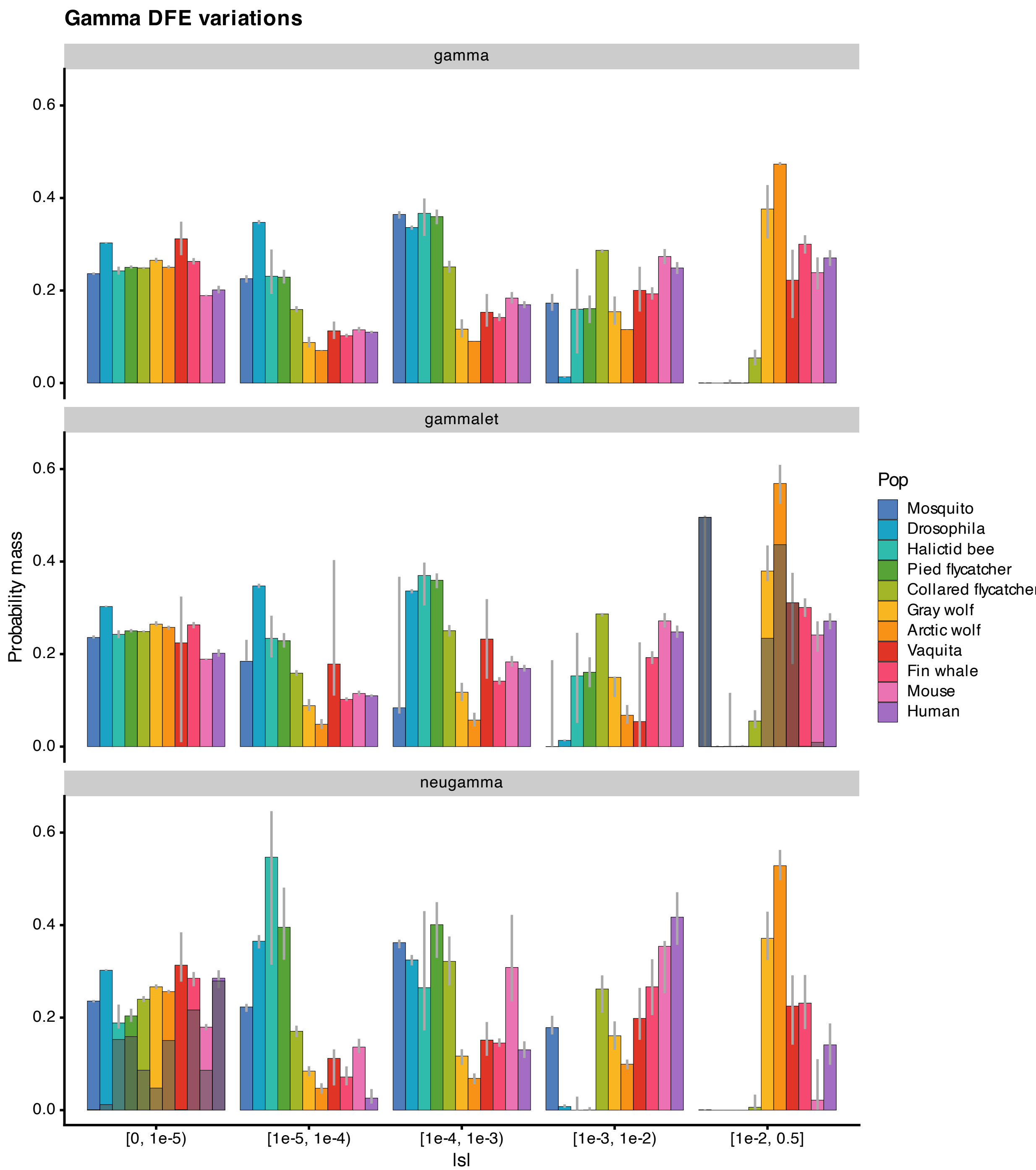

Figure J: Proportions of mutations in various categories of $|s|$. From left to right, mutations range from (nearly) neutral ($-10^{-5} < s \leq 0$) to strongly deleterious ($s \leq -0.01$). Different panels represent different assumptions for the functional forms of the DFE: gamma, neugamma, or gammalet. In the neugamma panel, the darker proportion at the left-most column ($-10^{-5} < s \leq 0$) represents the inferred point mass at neutrality. In the gammalet panel, the darker proportion at the right-most column ($s \leq -0.01$) represents the inferred point mass at lethality. Gray lines represent FIM-derived confidence intervals.

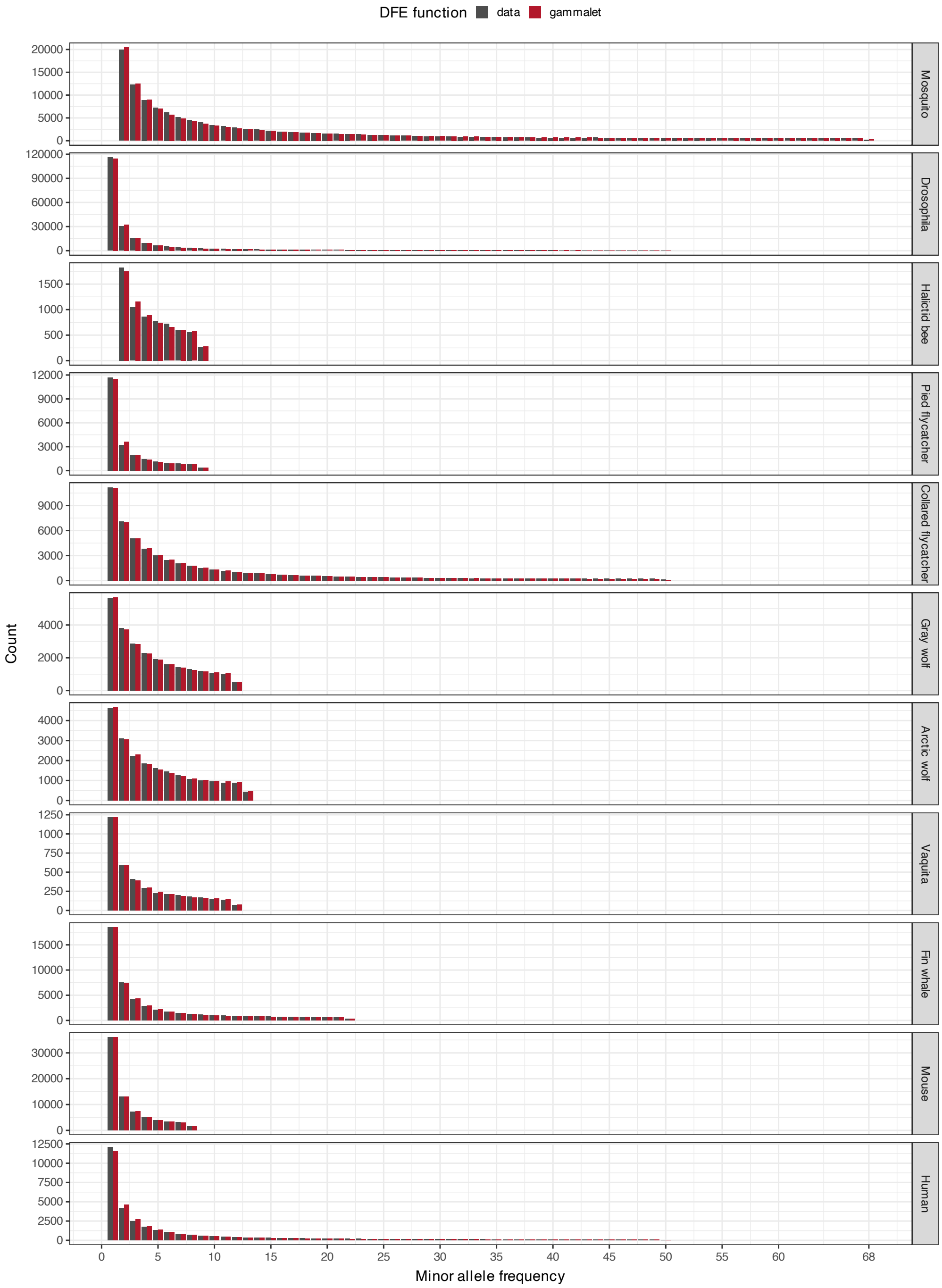

Figure K: Comparison of the observed nonsynonymous SFS (MIS-SFS) to the predicted SFS assuming the DFE is a mixture of a gamma distribution and a lethal point mass (gammalet DFE). The MIS-SFS data (gray) and expected SFS at the maximum-likelihood parameter estimates (red) are shown for each dataset.

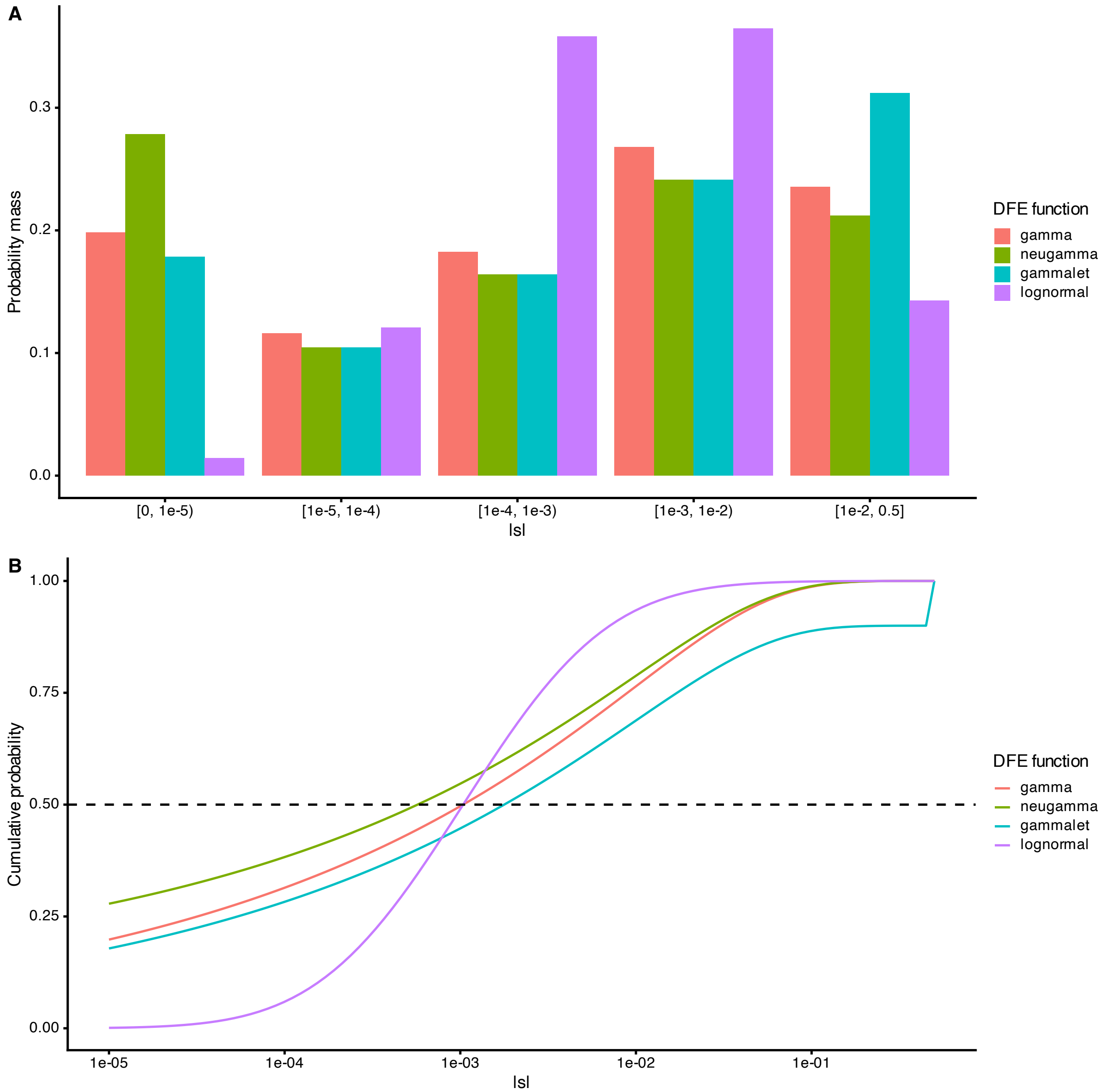

Figure L: (A) Proportions of mutations in various categories of $|s|$ and (B) the cumulative probability distribution for hypothetical deleterious-only DFE functions. The gamma distribution family (gamma, neugamma, and gammalet) is parameterized with shape $\alpha = 0.2$, scale $\beta' = 0.05$. 10% of neutral ($p_{\text{neu}} = 0.1$) or lethal mutations ($p_{\text{let}} = 0.1$) are parameterized for the neugamma and gammalet distribution, respectively. The lognormal distribution is parameterized so that it has similar $|s|_{\text{mean}}$ and median $|s|$ compared with the gamma distribution, with $\mu'_s = -6.87, \sigma = 1.50$. The neugamma and gammalet distributions are similar to the gamma distribution except for the added neutral and lethal weights, respectively. With a similar $|s|_{\text{mean}}$ of 0.01 and median $|s|$ of 0.001 compared to the gamma distribution, the lognormal distribution exhibits greater density at more deleterious $|s|$ values. The median of each distribution is marked with the dashed line in (B).

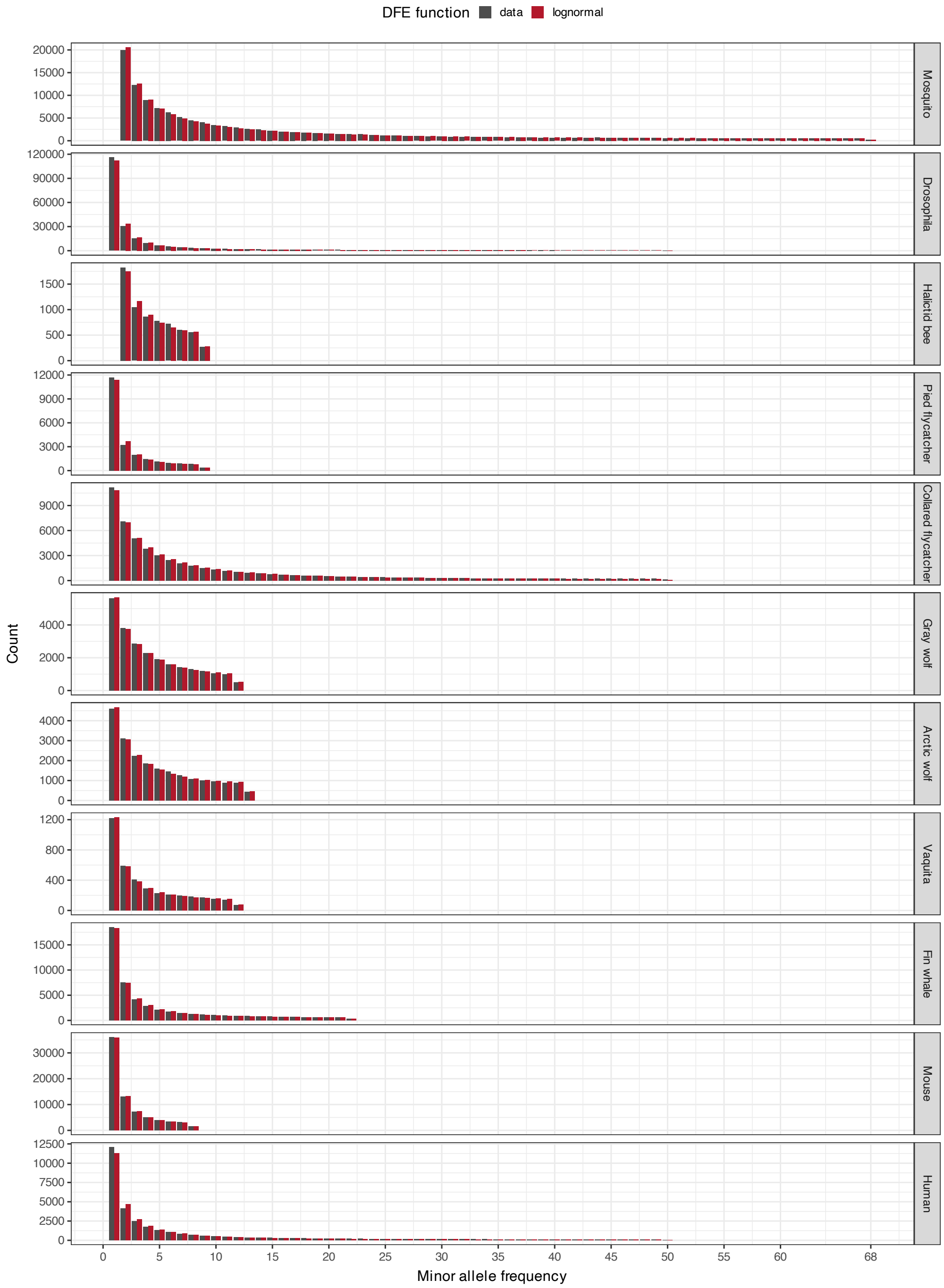

Figure M: Comparison of the observed nonsynonymous SFS (MIS-SFS) to the predicted SFS assuming the DFE follows a lognormal distribution. The MIS-SFS data (gray) and expected SFS at the maximum-likelihood parameter estimates (red) are shown for each dataset.

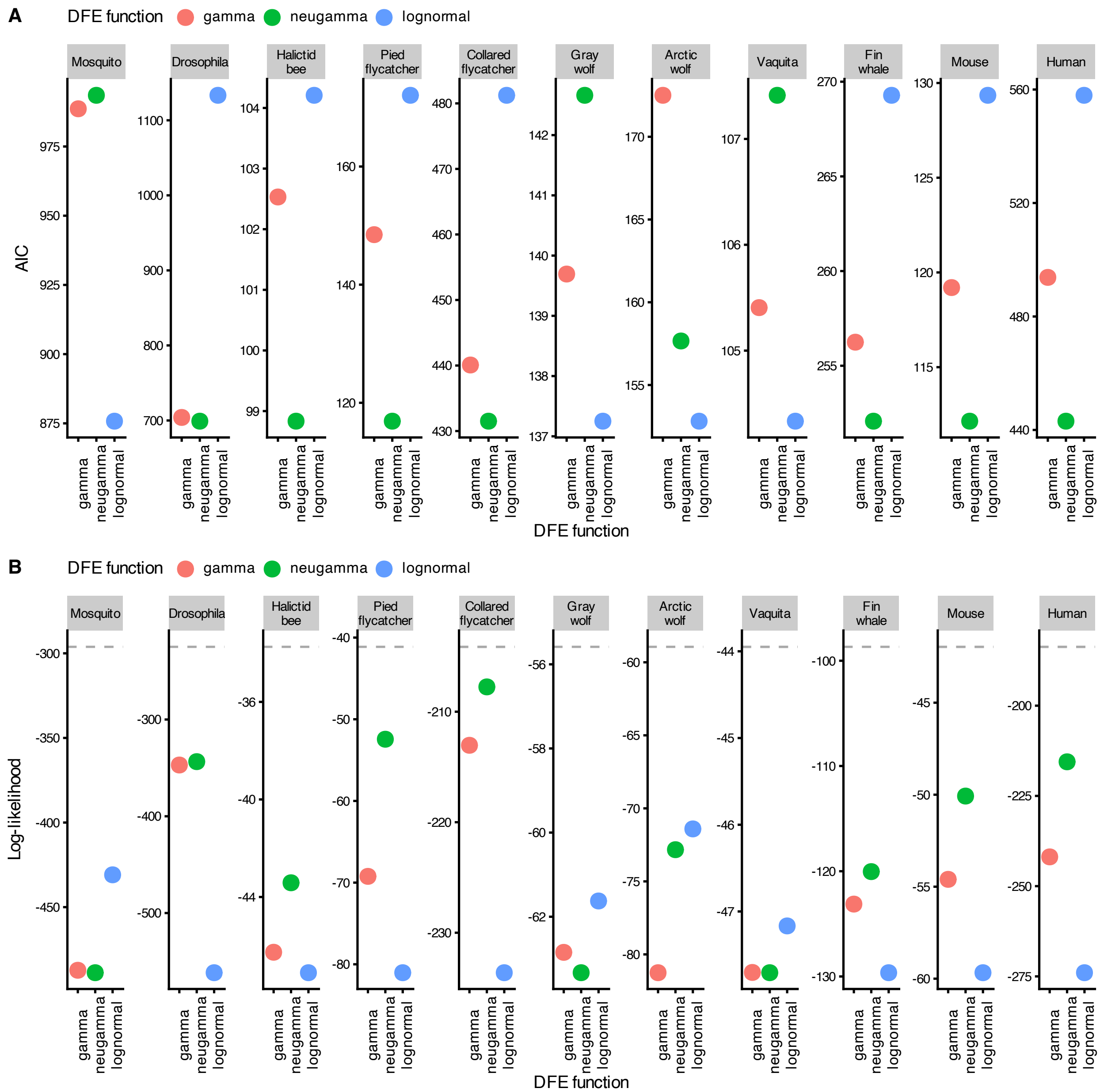

Figure N: Comparisons of the (A) AIC, and (B) log-likelihoods of the DFE inference assuming that the DFE follows a gamma, neugamma, or lognormal distribution. The gray dashed line in (B) represents the maximum log-likelihood derived from the data compared to itself.

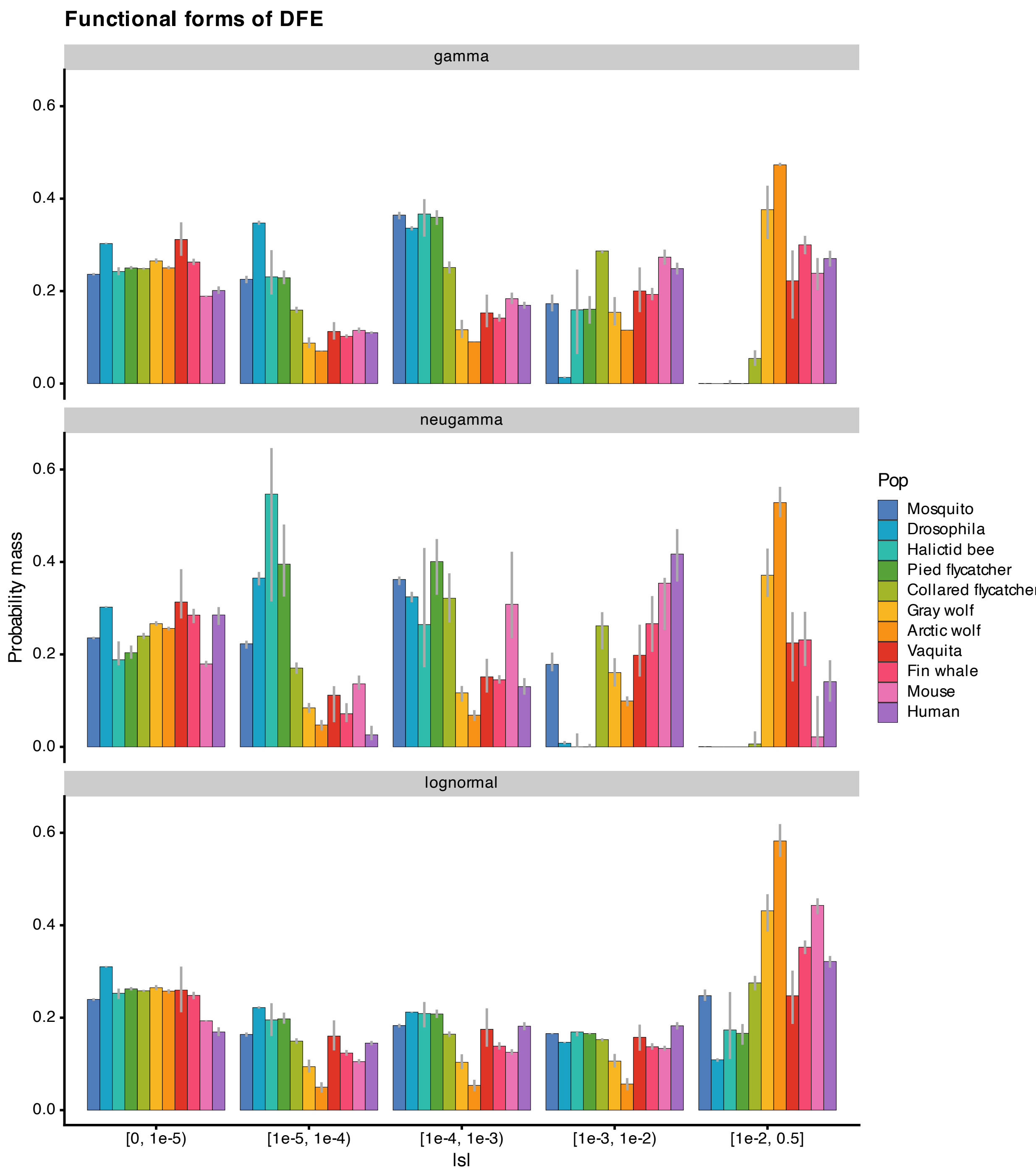

Figure O: Proportions of mutations in various categories of $|s|$. From left to right, mutations range from (nearly) neutral ($-10^{-5} < s \leq 0$) to strongly deleterious ($s \leq -0.01$). Different panels represent different assumptions for the functional forms of the DFE: gamma, neugamma, or lognormal. Gray lines represent FIM-derived confidence intervals.

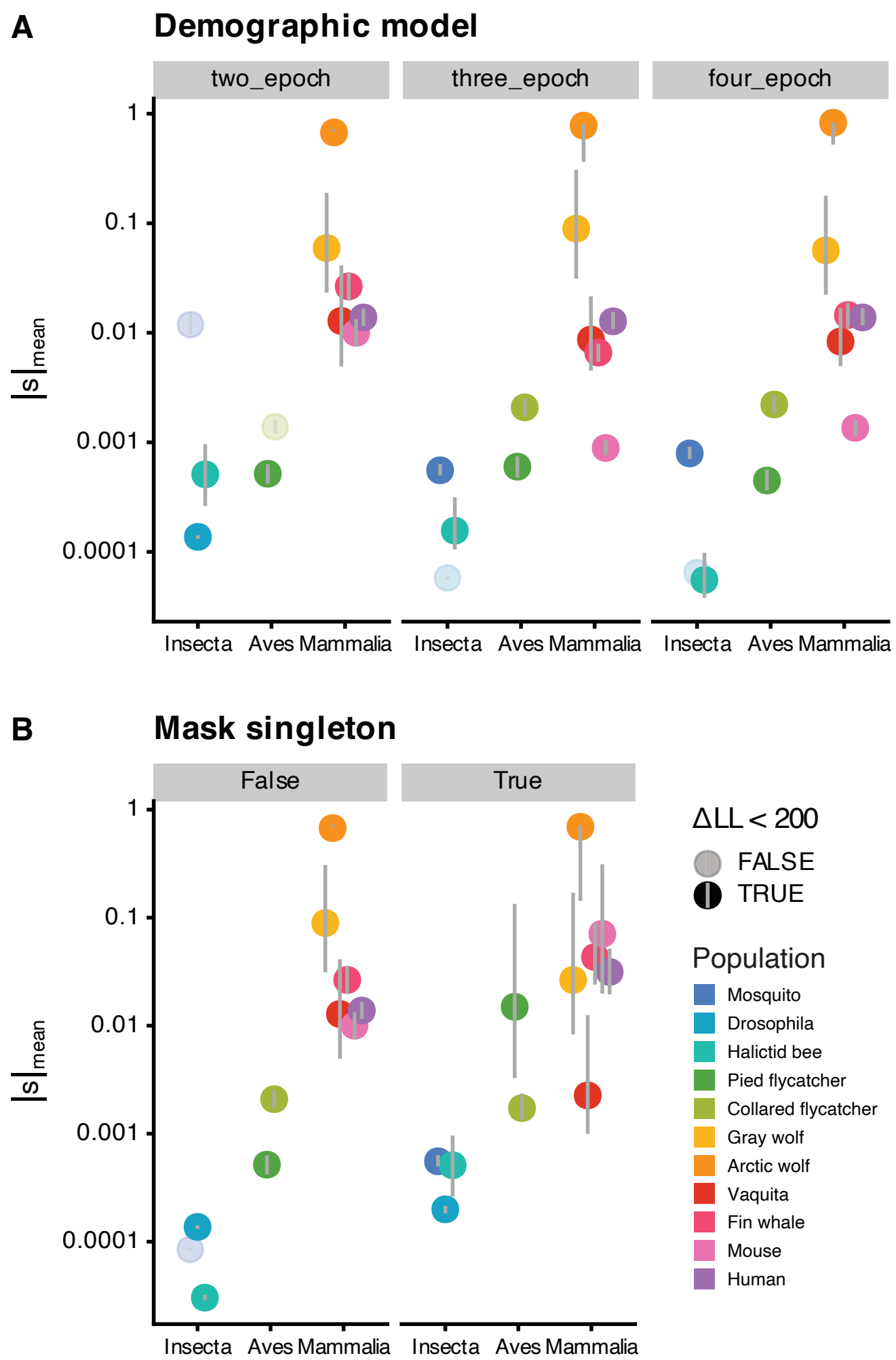

Figure P: The DFE inference is mostly robust to potential confounding factors. Assuming a gamma-distributed DFE, the mean selection coefficient ($|s|_{\text{mean}}$) for each species under different (A) demographic model assumptions and (B) singleton-masking treatments. Points with different colors represent different (sub)species. In DFE inferences that did not fit the data, defined as the log-likelihood difference between the data and model exceeds 200 ($\Delta LL < 200$ is FALSE), points are marked as translucent.

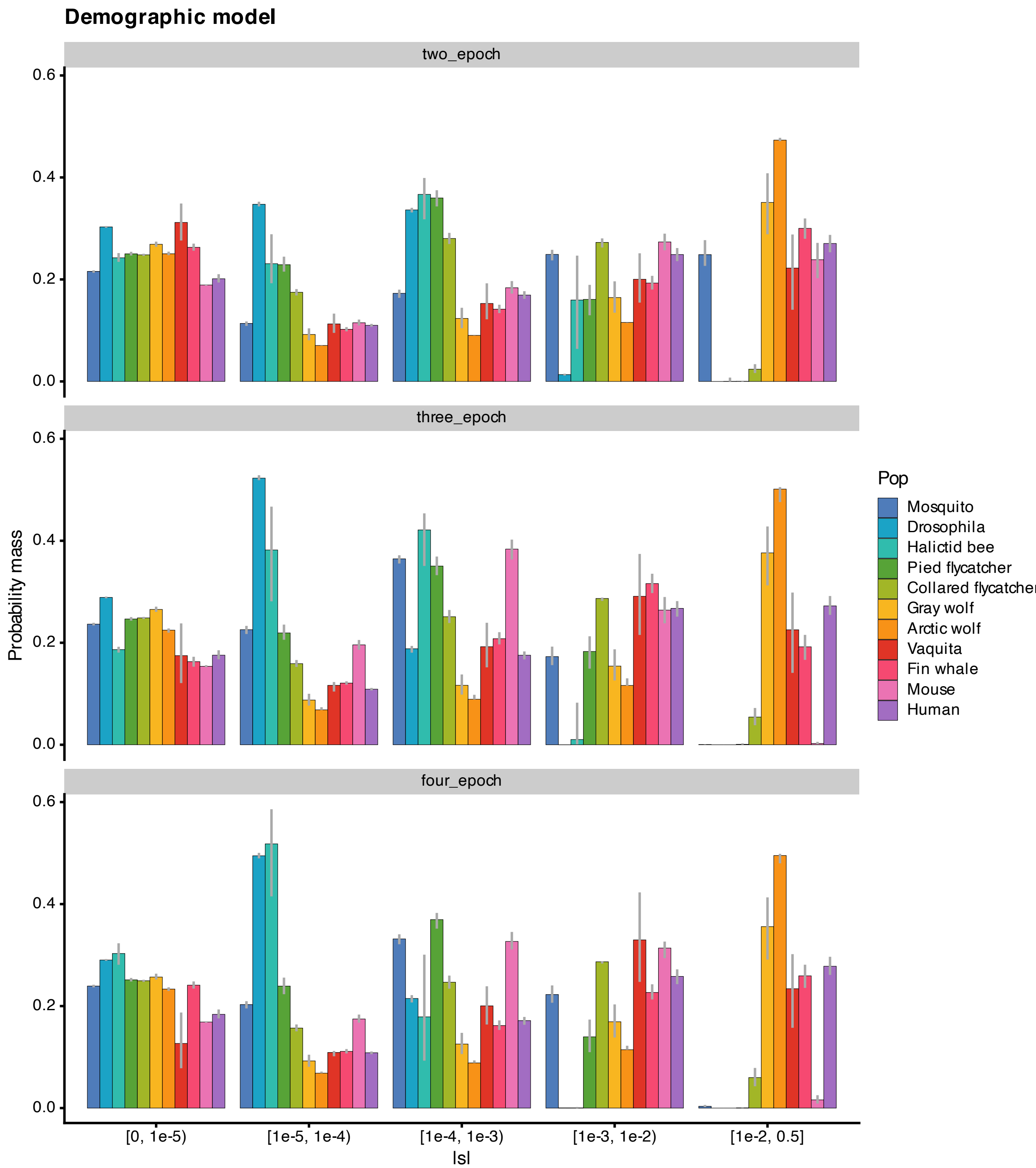

Figure Q: Proportions of mutations in various categories of $|s|$. From left to right, mutations range from (nearly) neutral ($-10^{-5} < s \leq 0$) to strongly deleterious ($s \leq -0.01$). Assuming a gamma-distributed DFE, different panels represent different assumptions for the underlying demographic models of the population: two-, three-, and four-epoch. Gray lines represent FIM-derived confidence intervals.

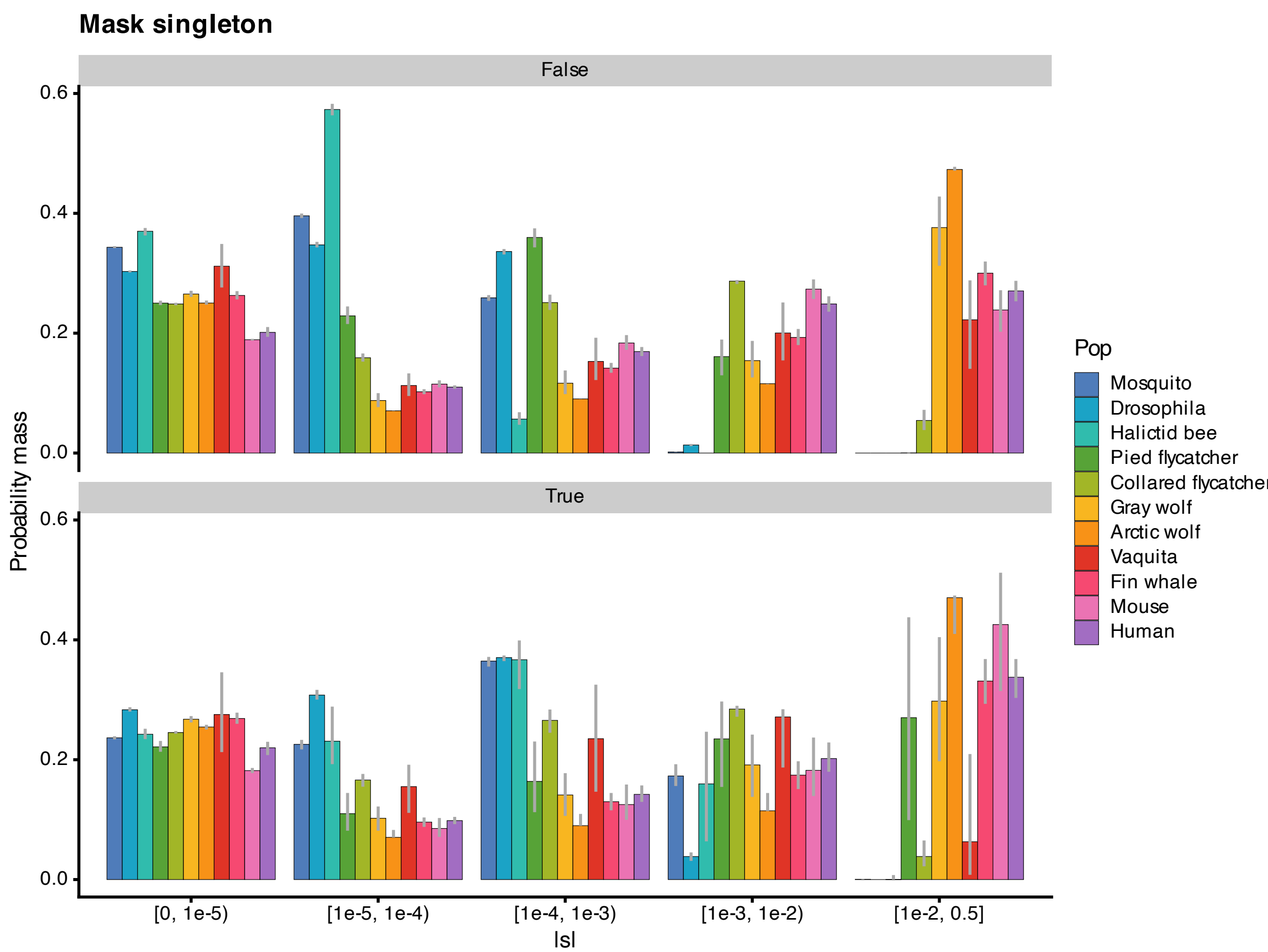

Figure R: Proportions of mutations in various categories of $|s|$. From left to right, mutations range from (nearly) neutral ($-10^{-5} < s \leq 0$) to strongly deleterious ($s \leq -0.01$). Assuming a gamma-distributed DFE, the top panel's inferred proportions are based on the full SFS and the bottom panel represents the inferred proportions of $|s|$ after masking singletons in the SFS. Gray lines represent FIM-derived confidence intervals.

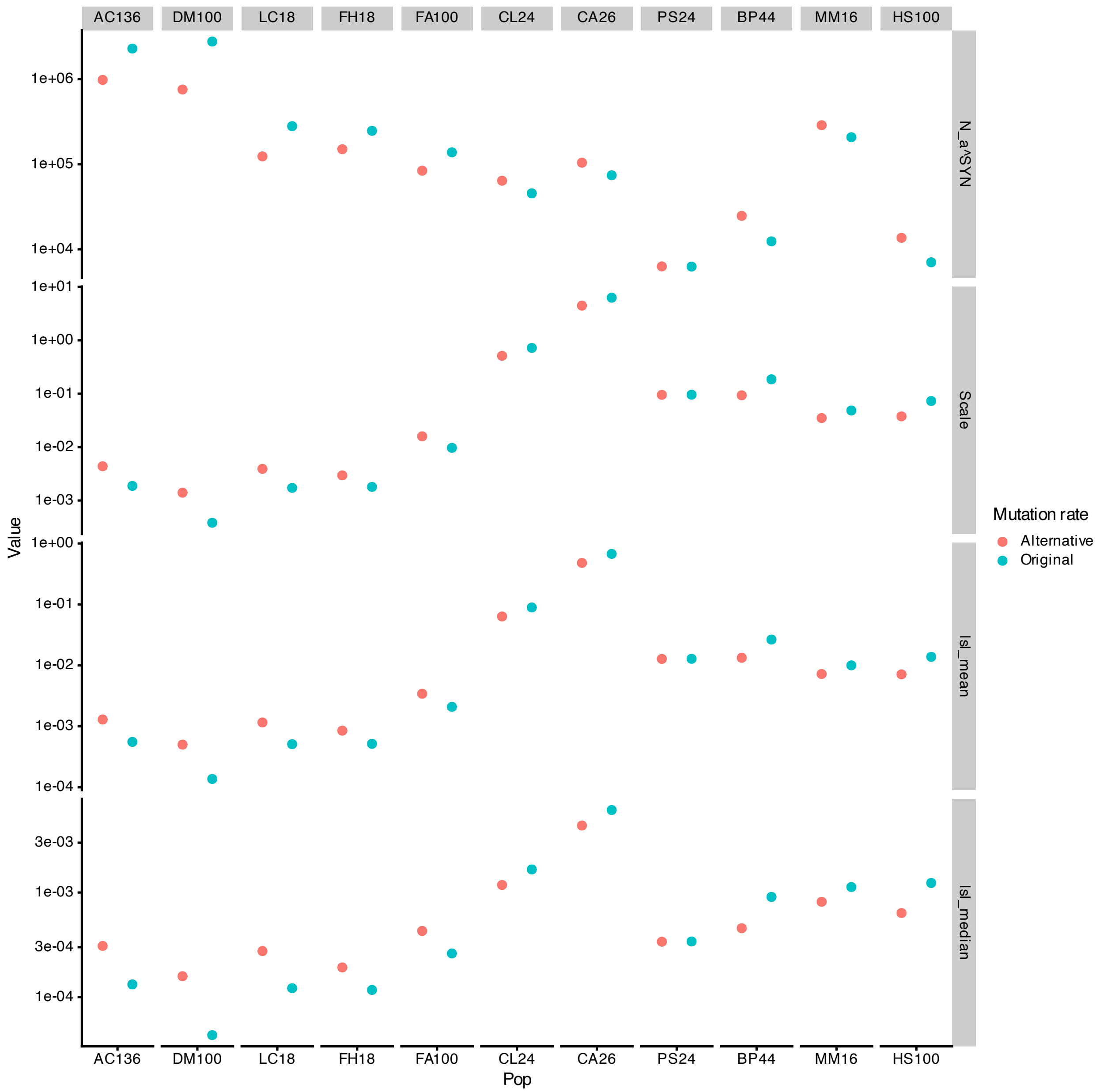

Figure S: Comparison of DFE estimates using the original and alternative mutation rates shown in Text B.

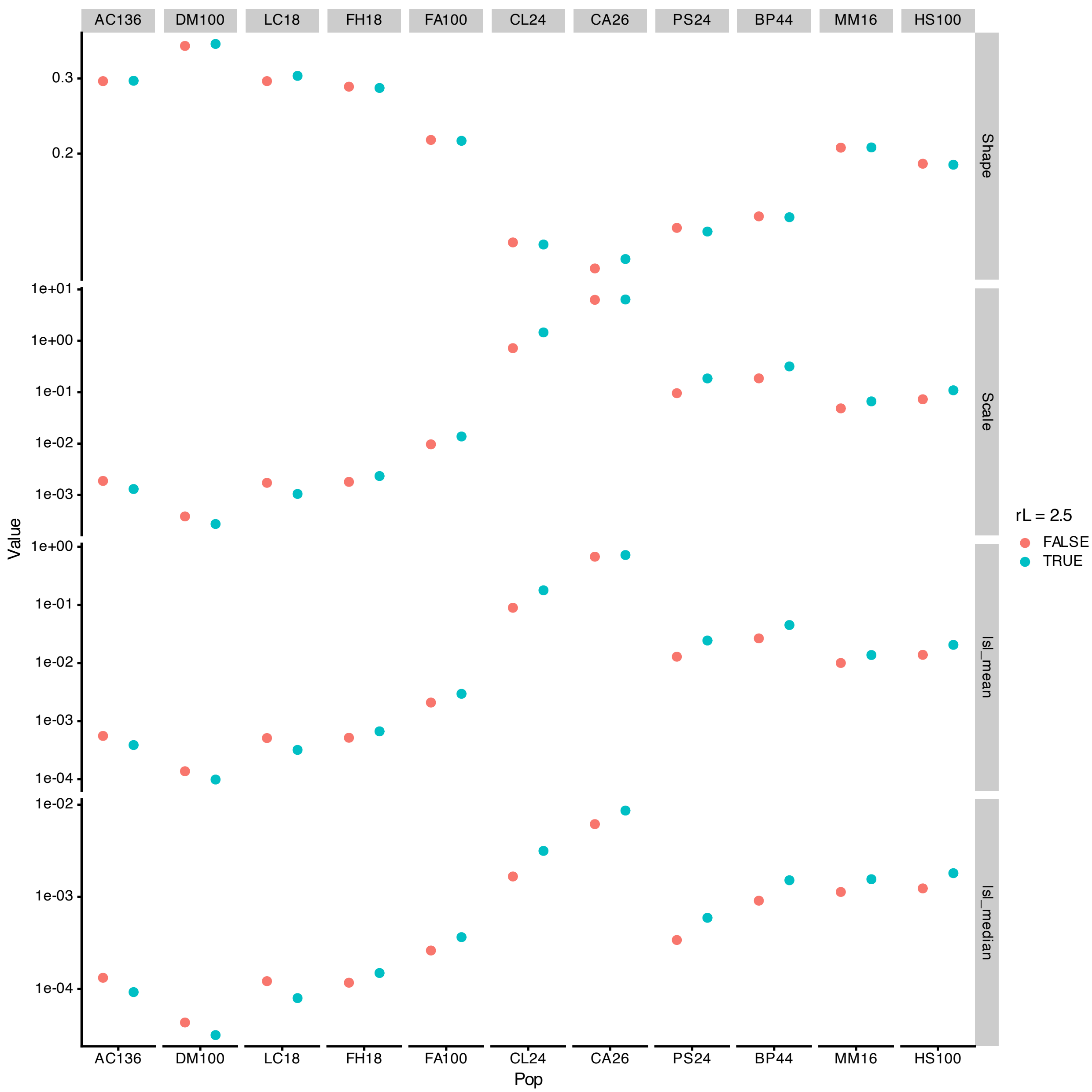

Figure T: Comparison of DFE estimates using the original nonsynonymous to synonymous mutation rate ratio ($r_L = 2.31$ for vertebrates and $r_L = 2.85$ for invertebrates), and the same ratio across species ($r_L = 2.5$).

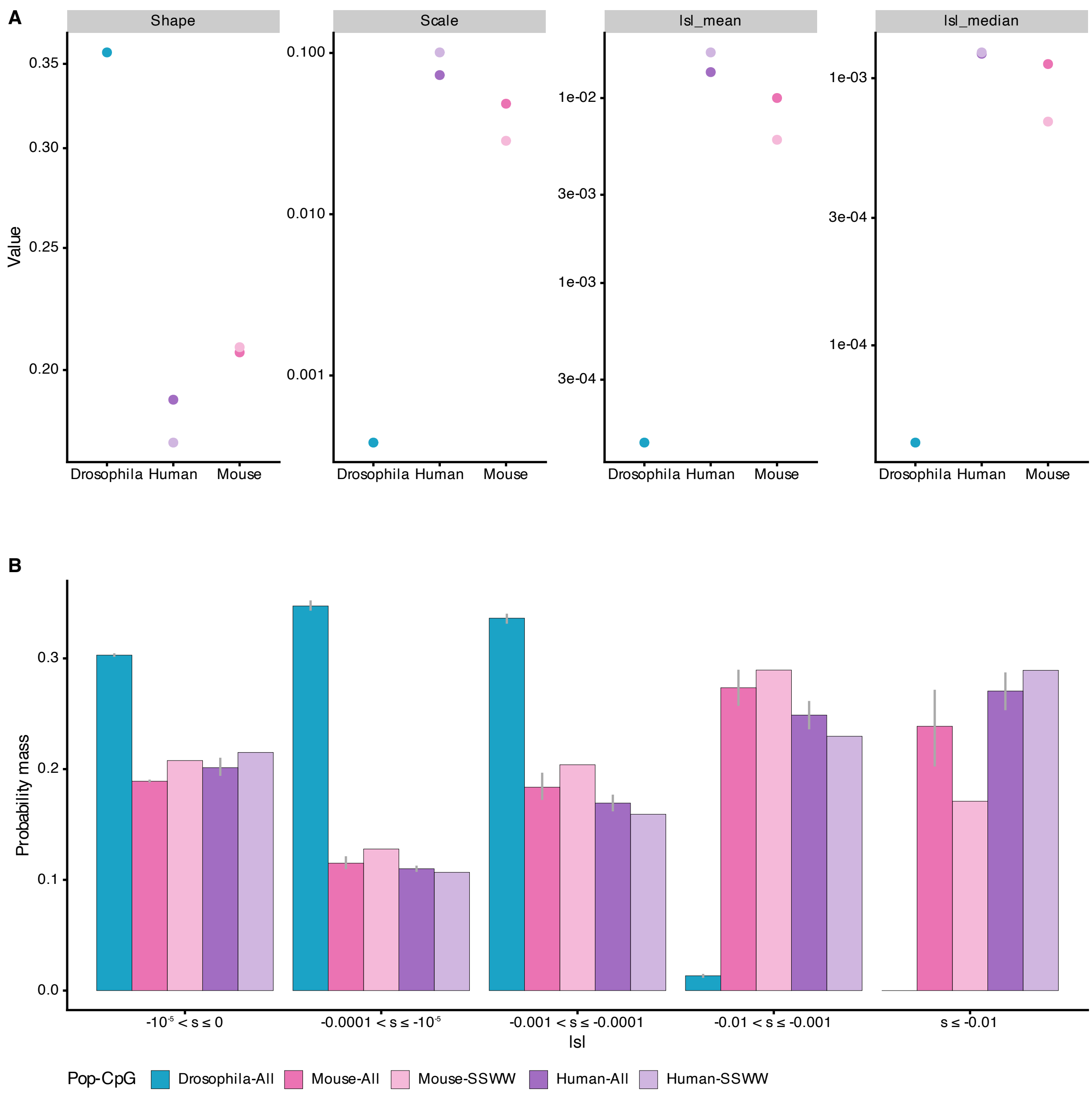

Figure U: Comparison of (A) DFE estimates and (B) proportions of mutations in various categories of $|s|$ using SFS constructed from all mutations or only strong-to-strong and weak-to-weak mutations in humans and mice.

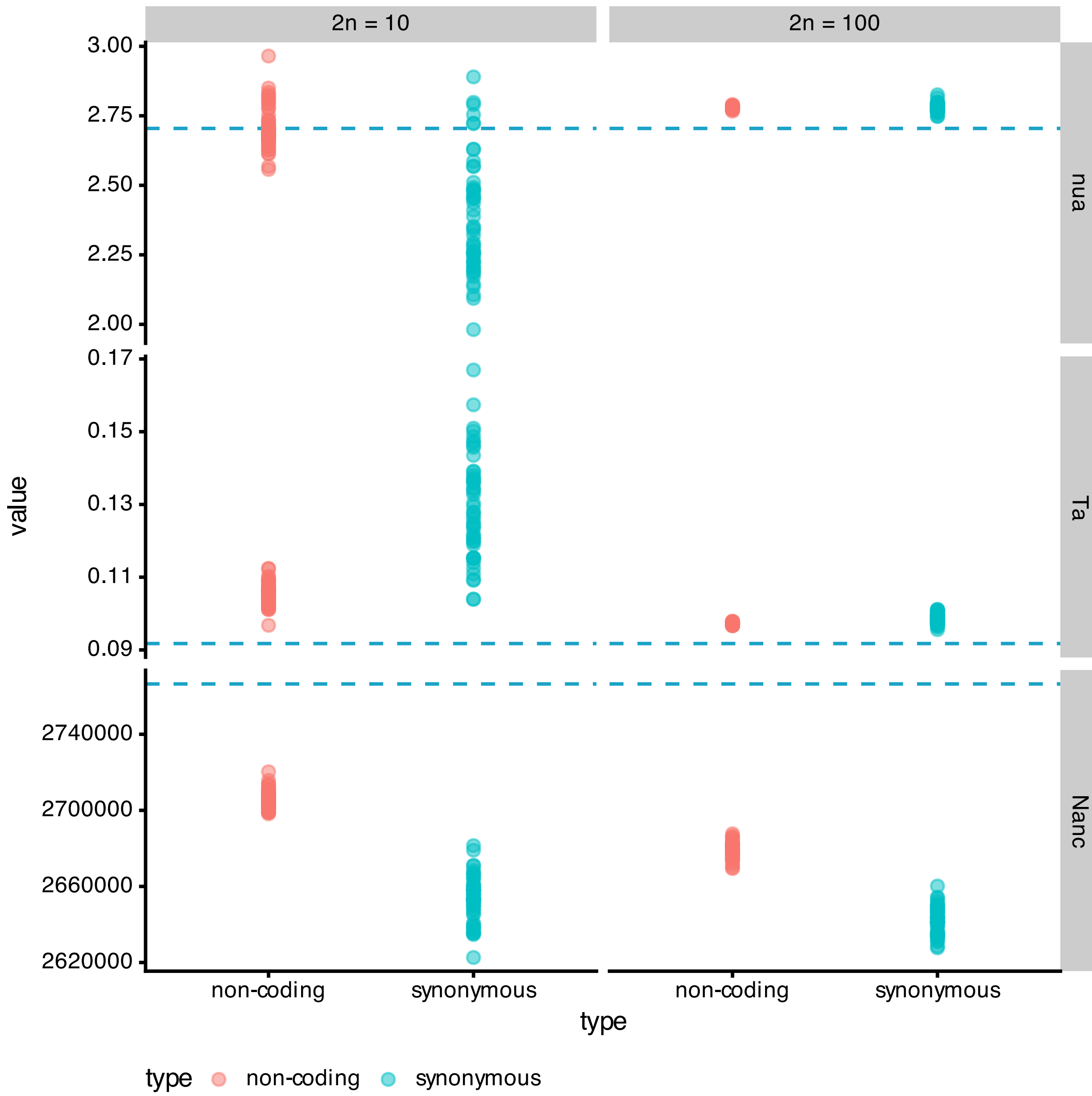

Figure V: Demographic inference from SLiM simulations using *Drosophila* demography and human DFE parameters. The left panel shows inference output from an SFS built from a sample of 10 chromosomes from the simulation, and the right panel is from an SFS from a sample of 100 chromosomes. From top to bottom, the ratio of the current population size to the ancestral population size (nua), the duration of size change (Ta), and the long-term population size (Nanc) are plotted. The true demographic parameter values used to simulate the data are marked with dashed lines.

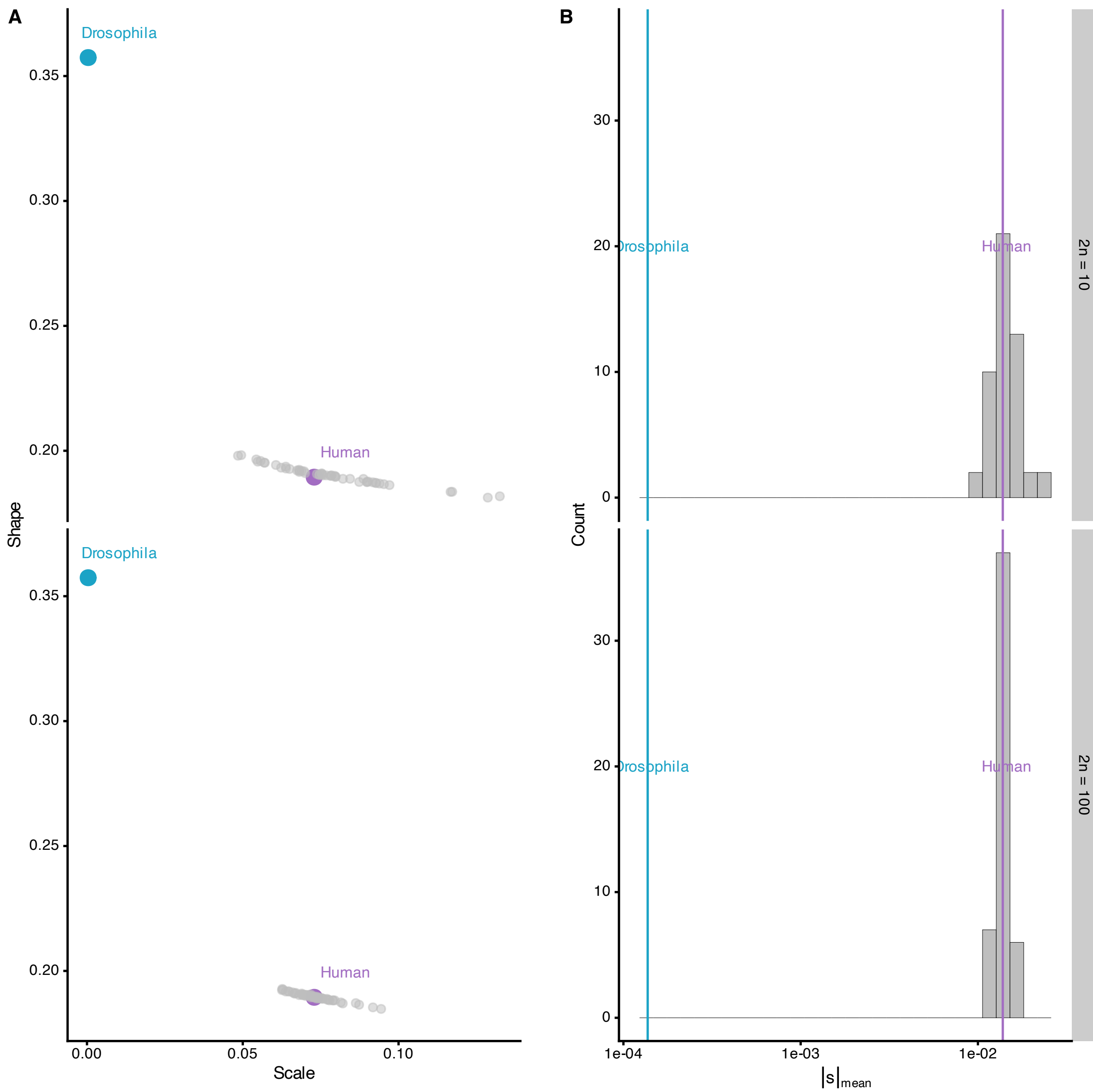

Figure W: DFE inference from SLiM simulations using *Drosophila* demography and human DFE parameters. (A) The inferred gamma DFE shape and scale parameters in the 50 bootstrapped SFSs are plotted against the human shape and scale parameters (purple points) and *Drosophila* shape and scale parameters (blue). The simulation was set with human DFE, and the inferred DFE is more similar to human than *Drosophila*, suggesting our DFE inference methods can detect strongly deleterious mutations even in large populations. (B) The histogram of the inferred $|s|_{\text{mean}}$ in the 50 bootstrapped SFSs (gray) is shown along with the human $|s|_{\text{mean}}$ (purple) and *Drosophila* $|s|_{\text{mean}}$ (blue). The inferred $|s|_{\text{mean}}$ in the simulation recovered the true human $|s|_{\text{mean}}$ rather than the *Drosophila*DFE. The top panel shows inference output from an SFS built from a sample of 10 chromosomes from the simulation, and the bottom panel is from an SFS from a sample of 100 chromosomes.

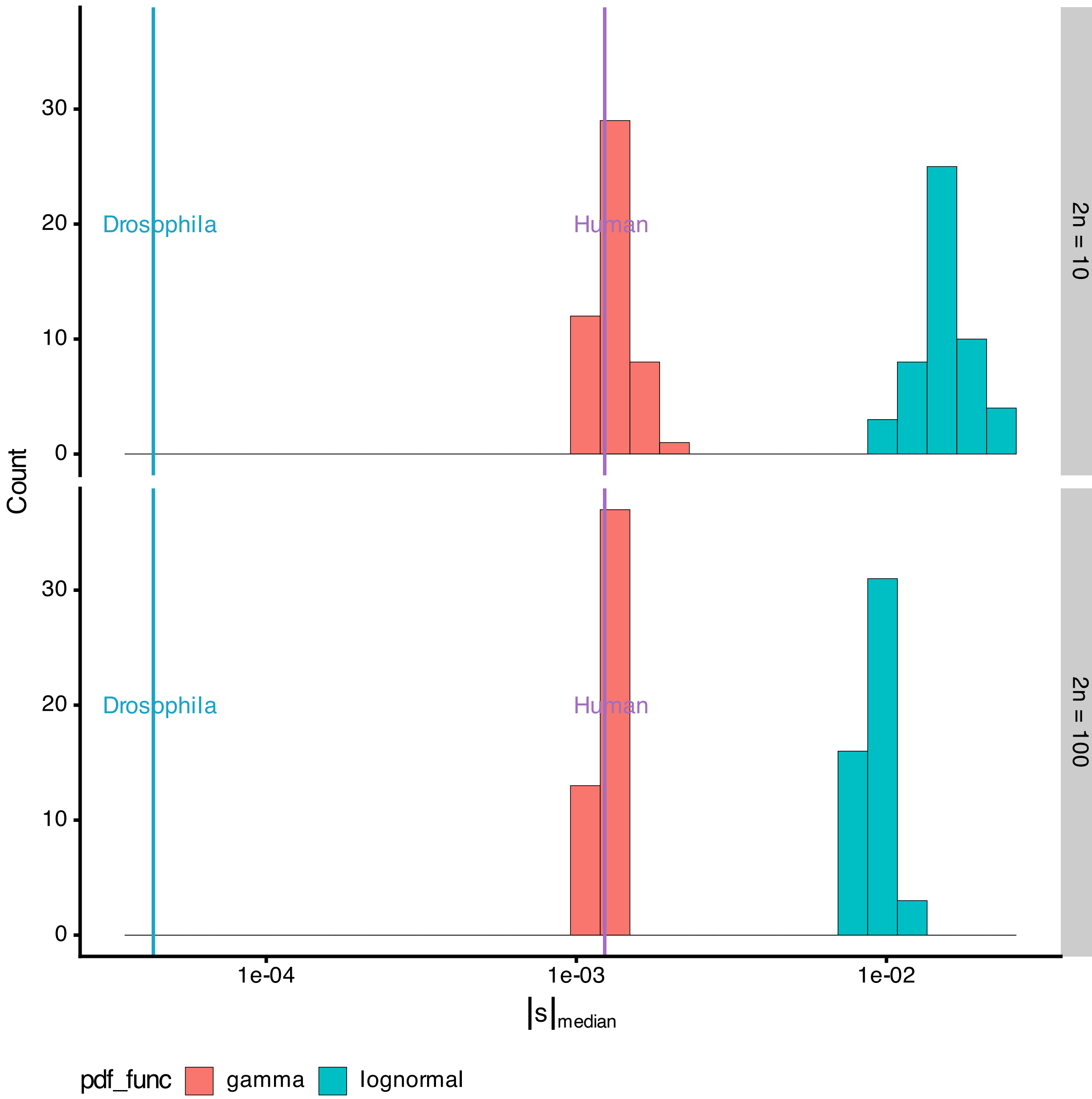

Figure X: DFE inference from SLiM simulations using *Drosophila* demography and a gamma-distributed human DFE parameters. The histogram of the inferred median $|s|$ ($|s|_{\text{median}}$) assuming a gamma-distributed (red) or lognormal-distributed (cyan) DFE. Inference output from the 50 bootstrapped SFSs assuming different DFE functions is shown along with the human $|s|_{\text{median}}$ (purple) and *Drosophila* $|s|_{\text{median}}$ (blue). The inferred gamma-distributed $|s|_{\text{median}}$ in the simulation recovered the assumed human $|s|_{\text{median}}$ rather than *Drosophila*. The inferred lognormal-distributed $|s|_{\text{median}}$ in the simulation is more deleterious than the assumed human $|s|_{\text{median}}$. The top panel shows inference output from an SFS built from a sample of 10 chromosomes from the simulation, and the bottom panel is from an SFS from a sample of 100 chromosomes.

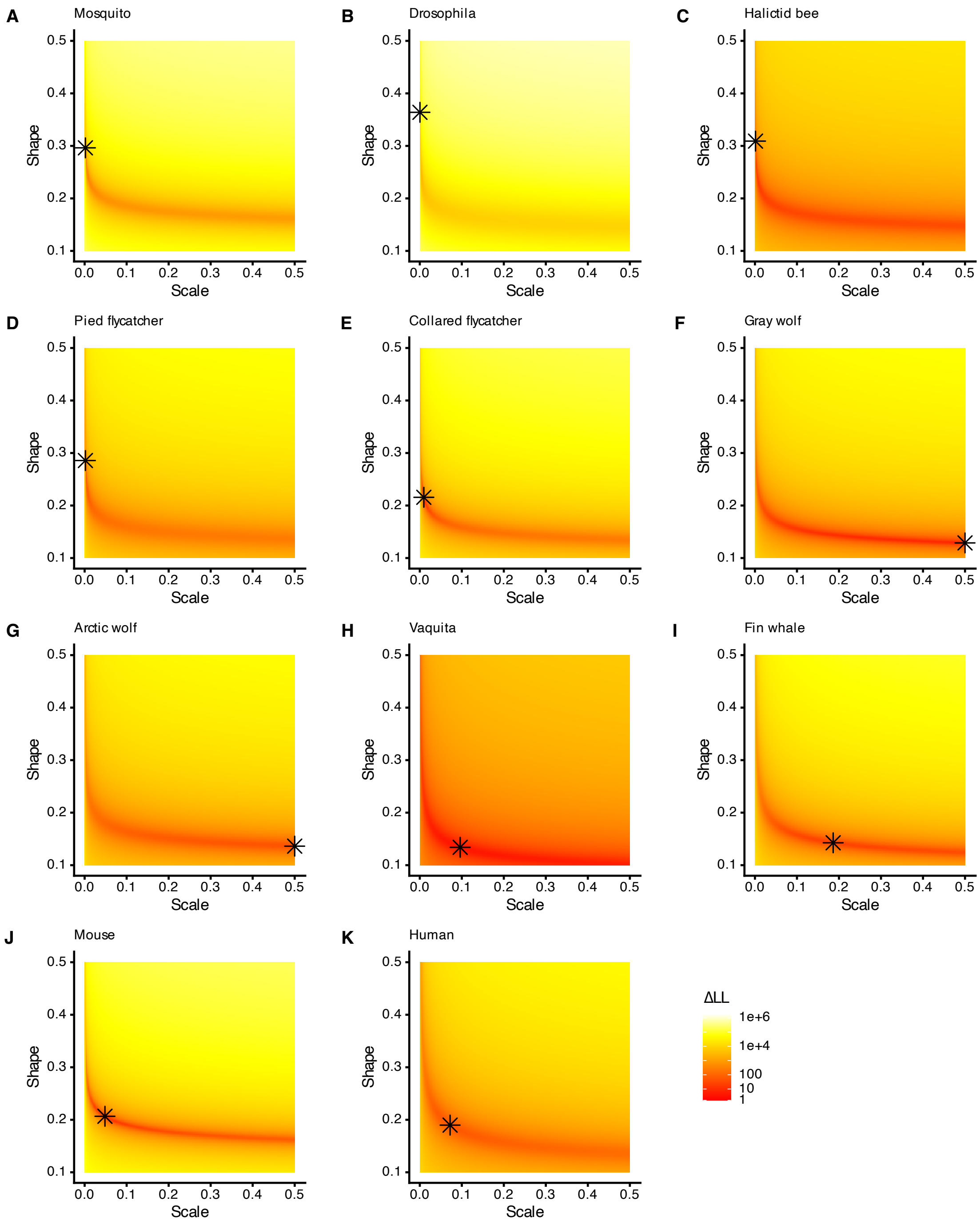

Figure Y: Full grid search results complementary to Fig 2. The log-likelihood surface for the shape (α) and scale (β') parameters is shown for each species. Background colors from yellow to red indicate the differences in log-likelihood for given parameters to data. On each log-likelihood surface, the maximum likelihood estimate (MLE) derived for the respective species is overlaid as the asterisk.

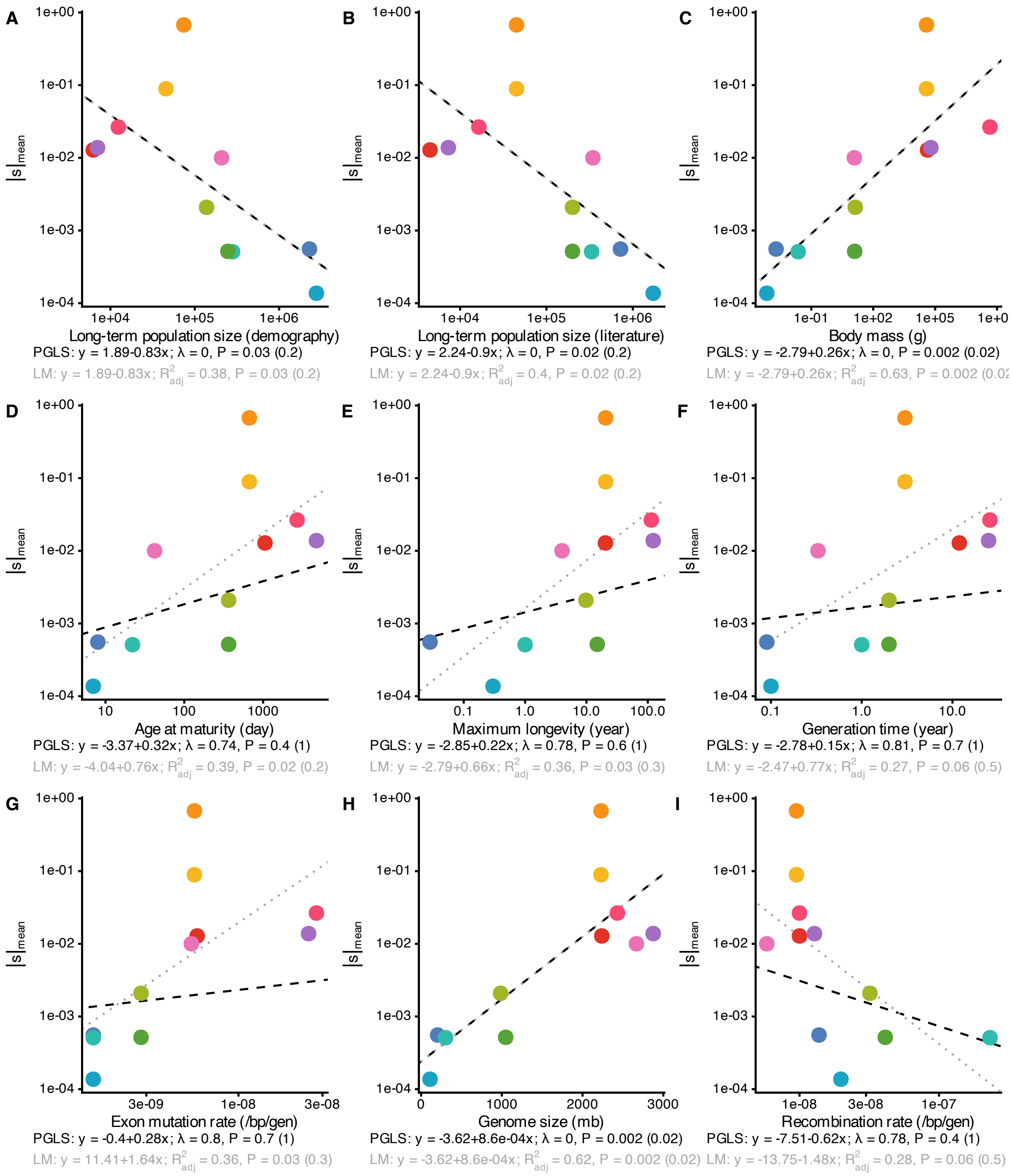

Figure Z: The phylogenetic generalized least squares model with simultaneously inferred phylogenetic signal (PGLS_λ) was fitted with $|s|_{\text{mean}}$ derived from the gamma DFE. The PGLS_λ results are overlaid as a dashed black line, with equations, inferred phylogenetic signal (λ), and likelihood-ratio derived p-value annotated at the bottom. The linear regression results are overlaid as a dotted gray line, with equations and p-values annotated at the bottom. Bonferroni-corrected p-values are annotated in the parenthesis. All axes are on a log10 scale, except for genome sizes.

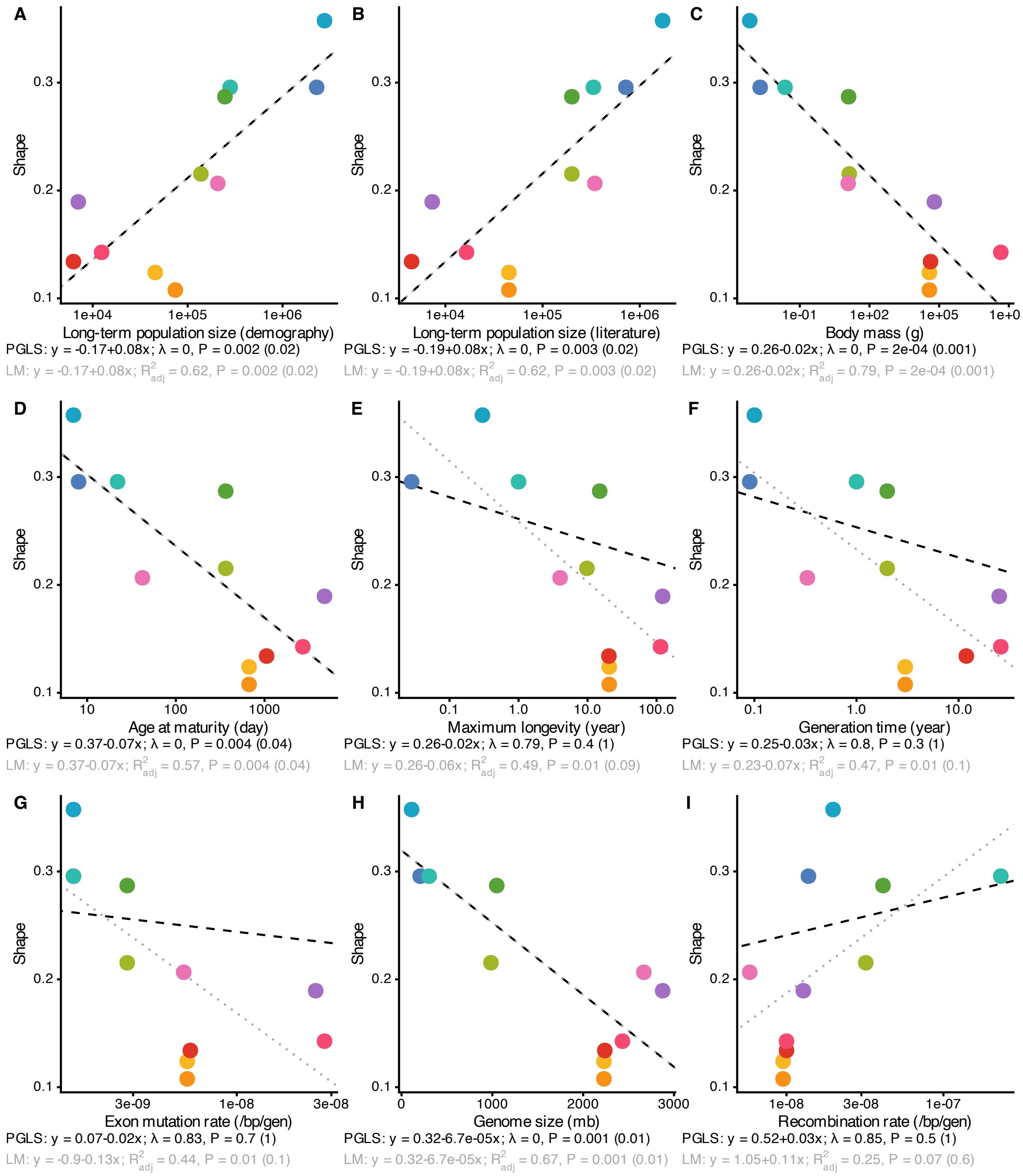

Figure AA: The phylogenetic generalized least squares model with simultaneously inferred phylogenetic signal ($PGLS_\lambda$) was fitted for the inferred gamma DFE's shape (α) parameter. The $PGLS_\lambda$ results are overlaid as a dashed black line, with equations, inferred phylogenetic signal (λ), and likelihood-ratio derived p-value annotated at the bottom. The linear regression results are overlaid as a dotted gray line, with equations and p-values annotated at the bottom. Bonferroni-corrected p-values are annotated in the parenthesis. The x-axes are on a log10 scale, except for genome sizes.

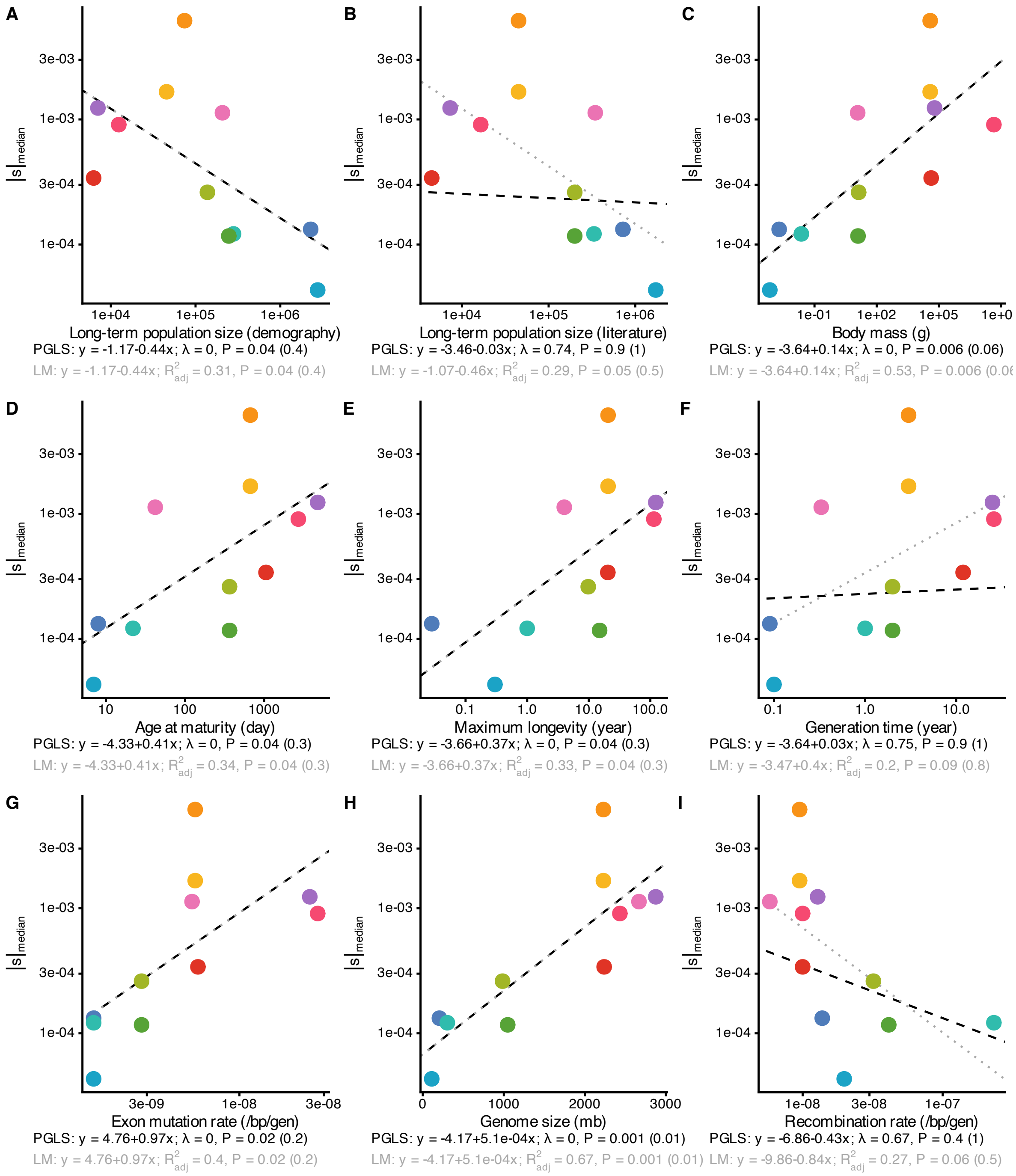

Figure AB: The phylogenetic generalized least squares model with simultaneously inferred phylogenetic signal (PGLS_λ) was fitted with $|s|_{\text{median}}$ derived from the gamma DFE. The PGLS_λ results are overlaid as a dashed black line, with equations, inferred phylogenetic signal (λ), and likelihood-ratio derived p-value annotated at the bottom. The linear regression results are overlaid as a dotted gray line, with equations and p-values annotated at the bottom. Bonferroni-corrected p-values are annotated in the parenthesis. All axes are on a log10 scale, except for genome sizes.

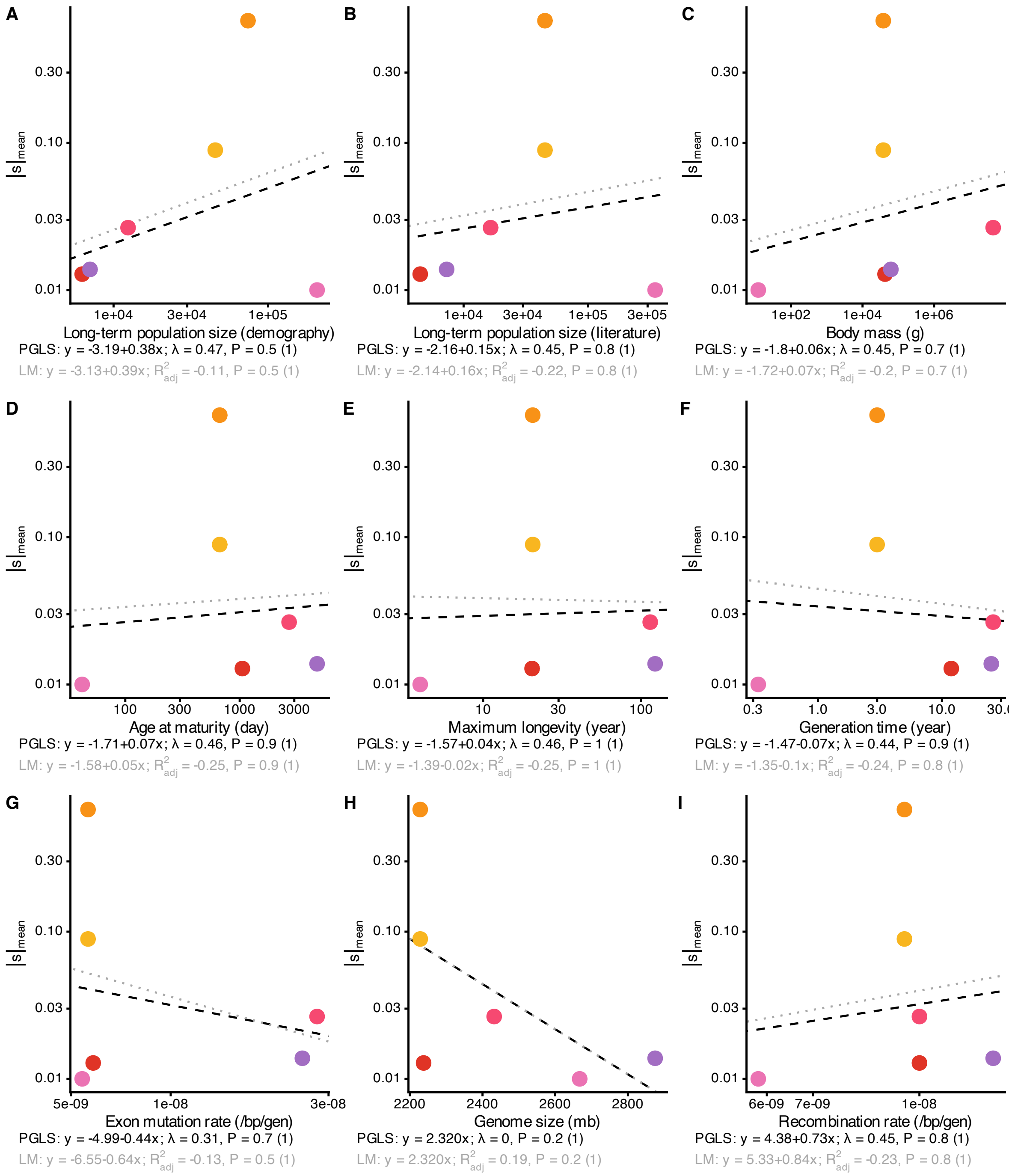

Figure AC: The phylogenetic generalized least squares model with simultaneously inferred phylogenetic signal ($PGLS_\lambda$) was fitted with $|s|_{\text{mean}}$ derived from the gamma DFE restricted to mammals. The $PGLS_\lambda$ results are overlaid as a dashed black line, with equations, inferred phylogenetic signal (λ), and likelihood-ratio derived p-value annotated at the bottom. The linear regression results are overlaid as dotted gray line, with equations and p-values annotated at the bottom. Bonferroni-corrected p-values are annotated in the parenthesis. All axes are on a log10 scale, except for genome sizes.

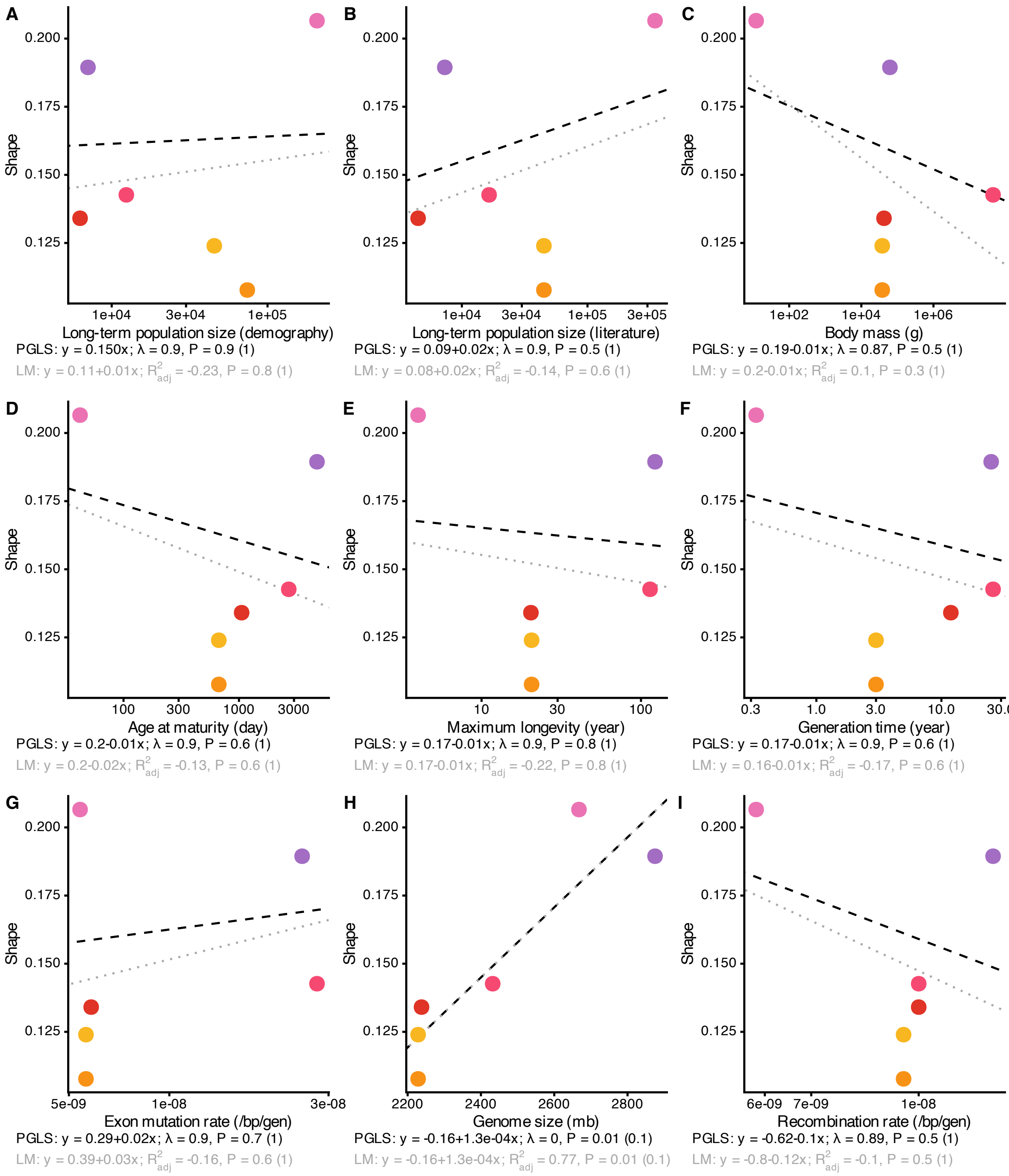

Figure AD: The phylogenetic generalized least squares model with simultaneously inferred phylogenetic signal ($PGLS_{\lambda}$) was fitted for the inferred gamma DFE's shape (α) parameter restricted to mammals. The $PGLS_{\lambda}$ results are overlaid as a dashed black line, with equations, inferred phylogenetic signal (λ), and likelihood-ratio derived p-value annotated at the bottom. The linear regression results are overlaid as a dotted gray line, with equations and p-values annotated at the bottom. Bonferroni-corrected p-values are annotated in the parenthesis. The x-axes are on a log10 scale, except for genome sizes.

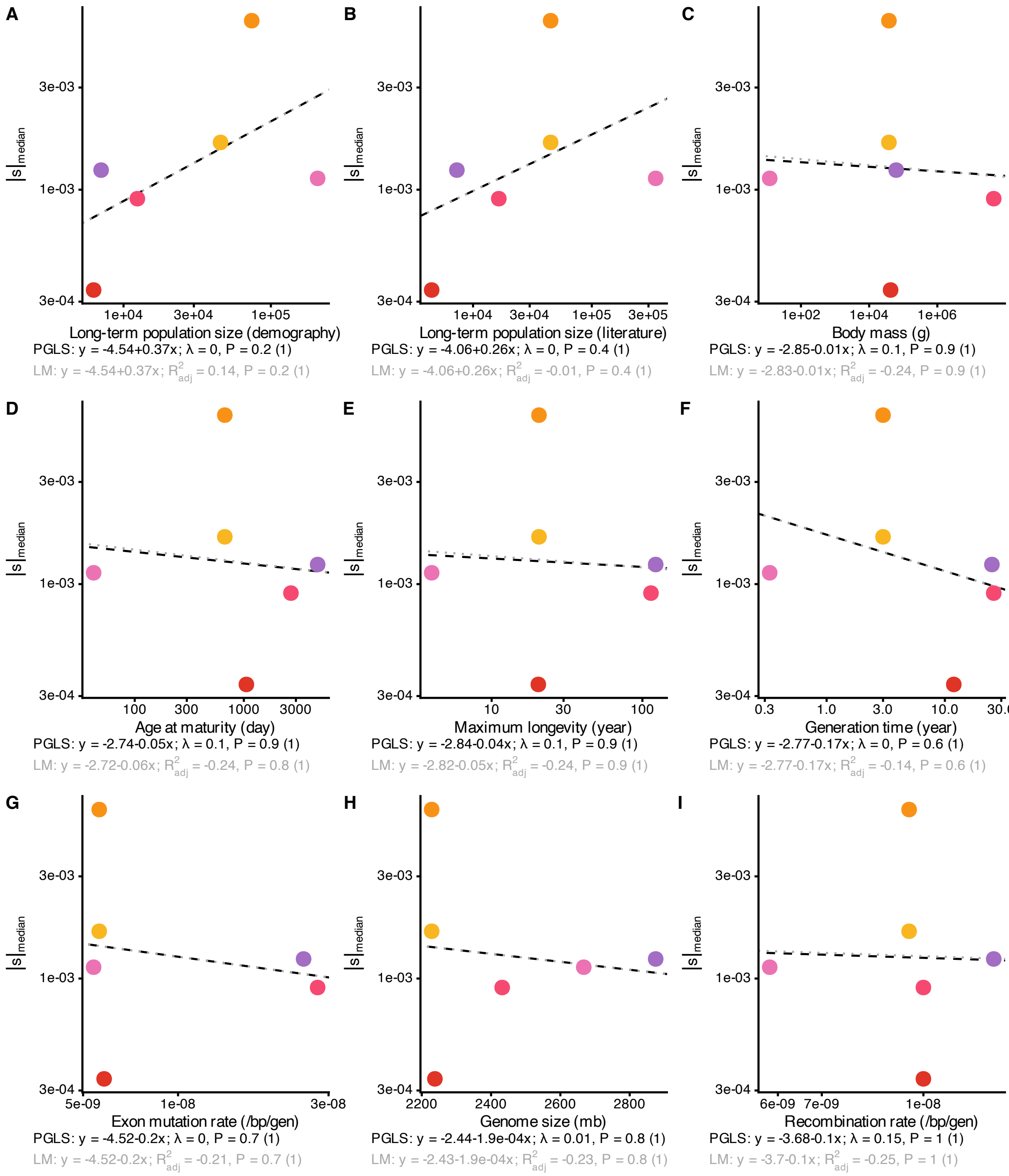

Figure AE: The phylogenetic generalized least squares model with simultaneously inferred phylogenetic signal ($PGLS_\lambda$) was fitted with $|s|_{\text{median}}$ derived from the gamma DFE restricted to mammals. The $PGLS_\lambda$ results are overlaid as a dashed black line, with equations, inferred phylogenetic signal (λ), and likelihood-ratio derived p-value annotated at the bottom. The linear regression results are overlaid as a dotted gray line, with equations and p-values annotated at the bottom. Bonferroni-corrected p-values are annotated in the parenthesis. All axes are on a log10 scale, except for genome sizes.

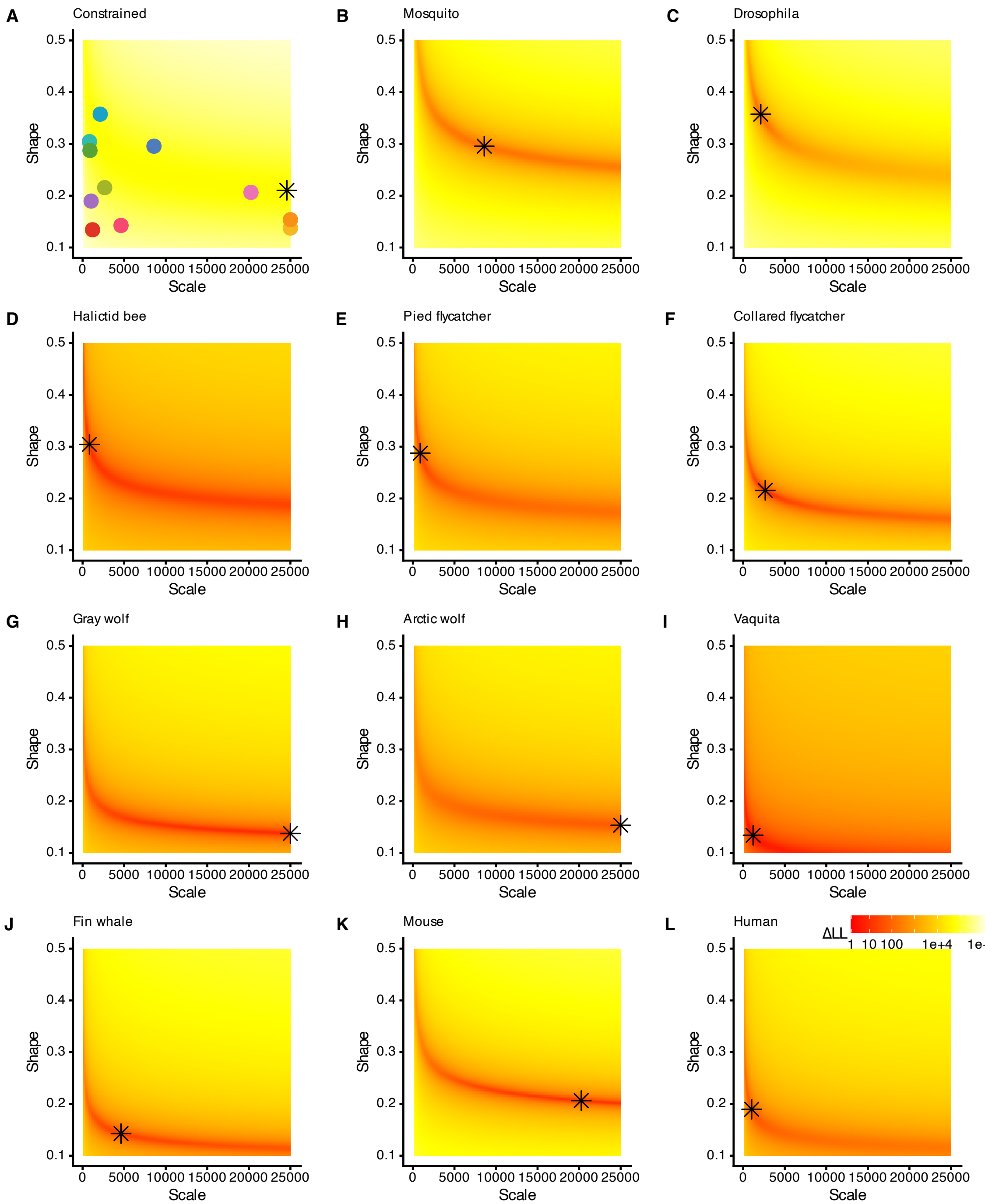

Figure AF: Full grid search results for the population-scaled DFE. The log-likelihood surface for the shape (α) and population-scaled scale (β) parameters are shown (A) under the constrained model where all species have the same parameters, or (B-L) under alternative models allowing each species' parameters to vary across datasets. Background colors from yellow to red indicate the differences in log-likelihood for given parameters to data. The color schemes are consistent with Fig 2. On each log-likelihood surface, the maximum likelihood estimate (MLE) derived for (A) the constrained model or (B-L) respective species is overlaid as the asterisk. In (A), the colored points show MLEs derived from each species' grid search.

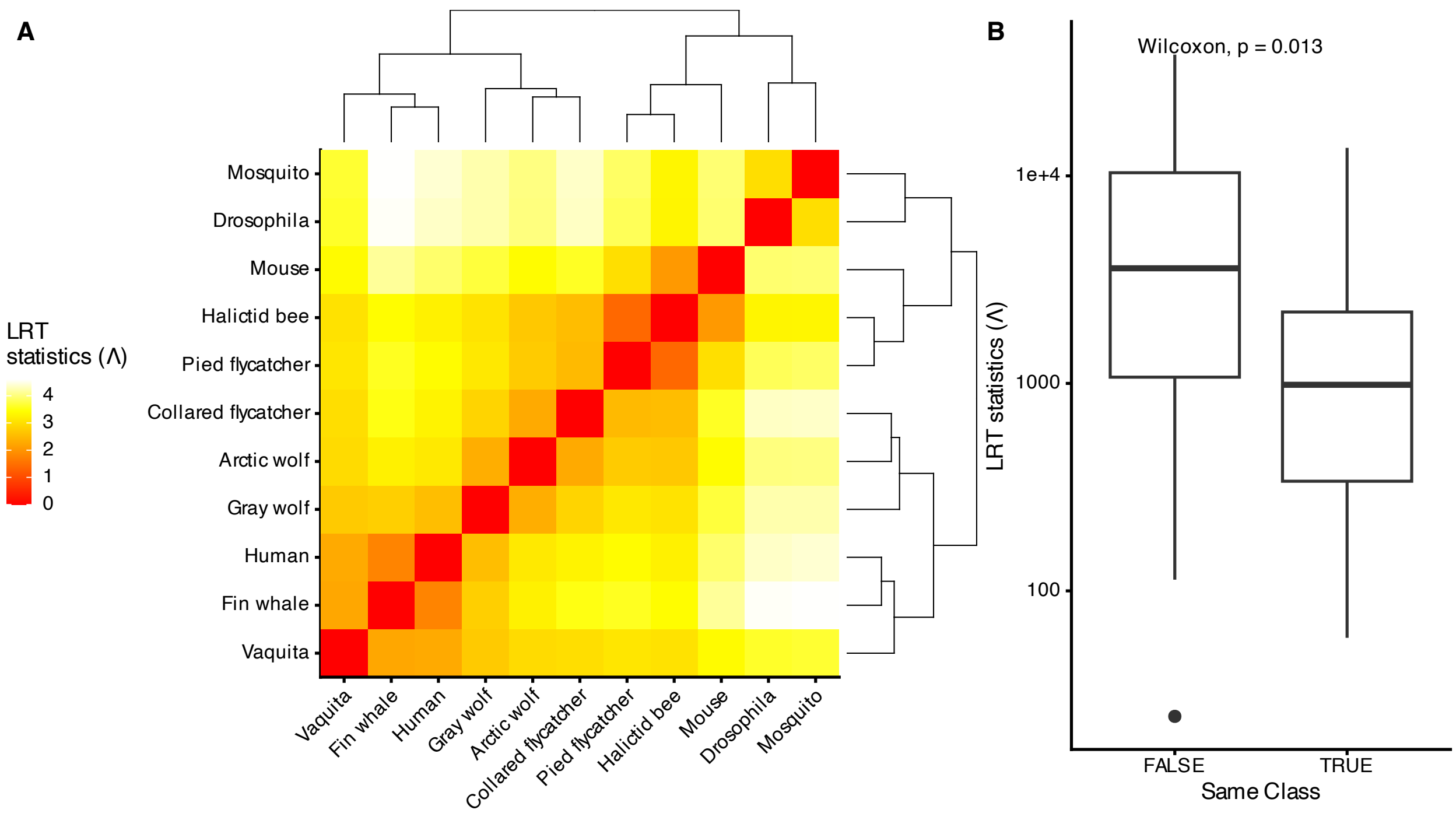

Figure AG: Pairwise likelihood ratio test (LRT) statistics for the population-scaled DFE. (A) Pairwise LRT statistics for each species pair are colored on a log10 scale ($\log_{10}(\Lambda)$) and hierarchically clustered. Darker cells represent more similar population-scaled DFE estimates in the species compared, such as fin whales and humans, whereas lighter cells represent more distinct DFEs, such as fin whales and *Drosophila*. The dendrogram derived from hierarchical clustering is annotated. (B) The boxplot of Λ values, categorized by whether the species pair belongs to the same taxonomic Class. The y-axis is on a log10 scale.

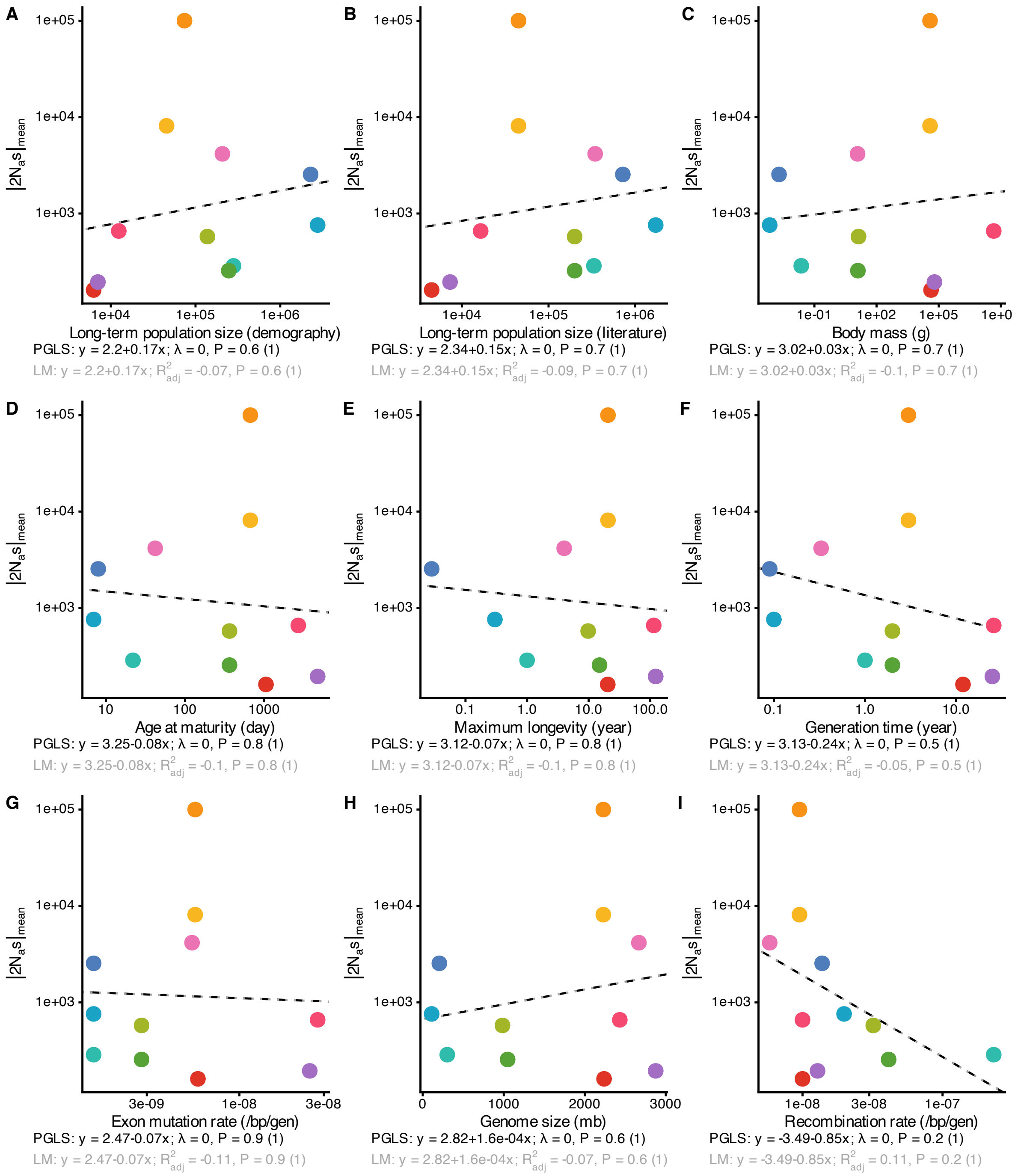

Figure AH: The phylogenetic generalized least squares model with simultaneously inferred phylogenetic signal ($PGLS_\lambda$) was fitted with $|2N_a s|_{\text{mean}}$ derived from the gamma DFE. The $PGLS_\lambda$ results are overlaid as a dashed black line, with equations, inferred phylogenetic signal (λ), and likelihood-ratio derived p-value annotated at the bottom. The linear regression results are overlaid as dotted gray line, with equations and p-values annotated at the bottom. Bonferroni-corrected p-values are annotated in the parenthesis. All axes are on a log10 scale, except for genome sizes.

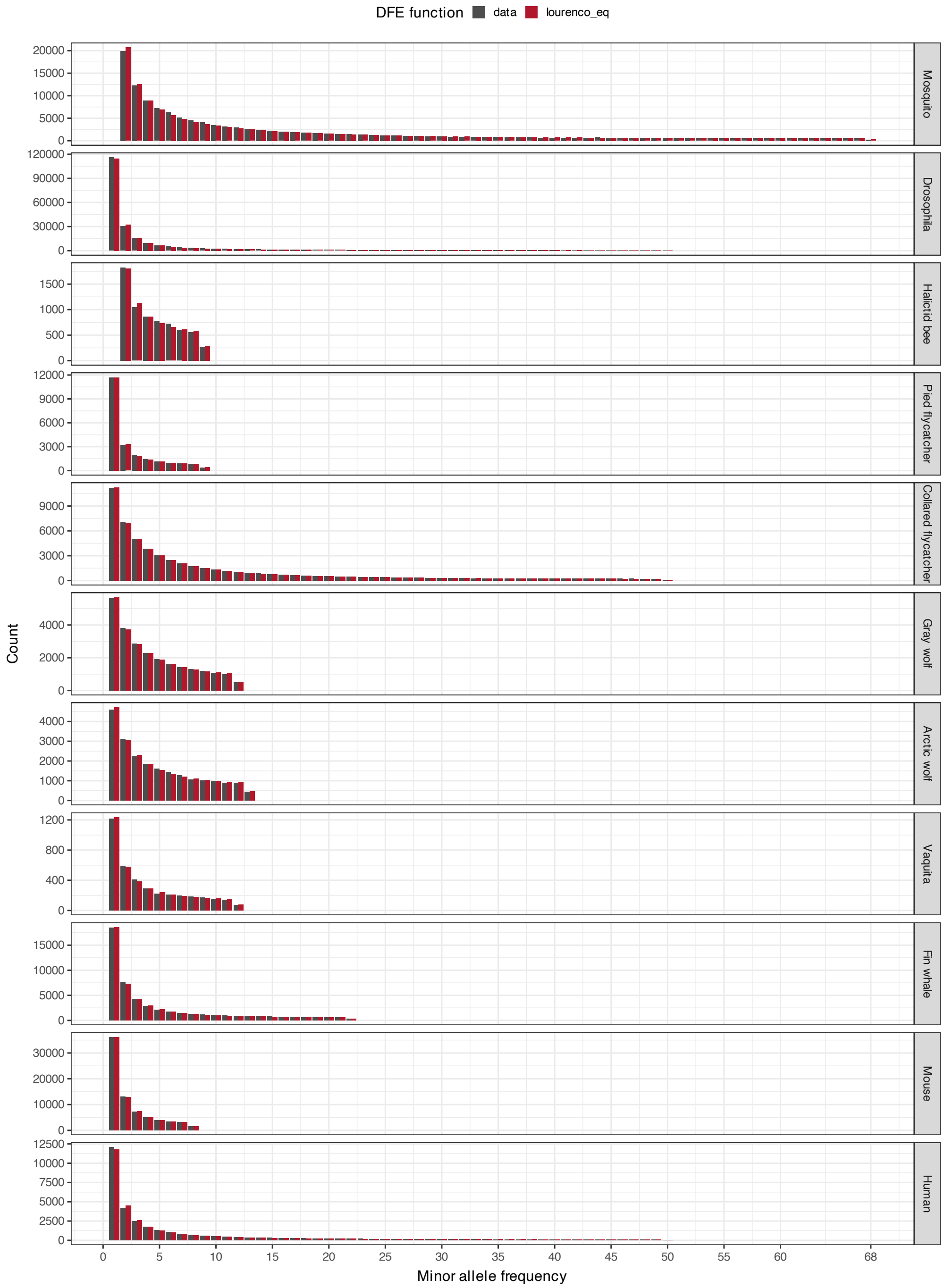

Figure AI: Comparison of the observed nonsynonymous SFS (MIS-SFS) to the predicted SFS assuming the DFE follows the Fisher's geometric model derived distribution [42]. The MIS-SFS data (gray) and expected SFS at the maximum-likelihood parameter estimates (red) are shown for each dataset.

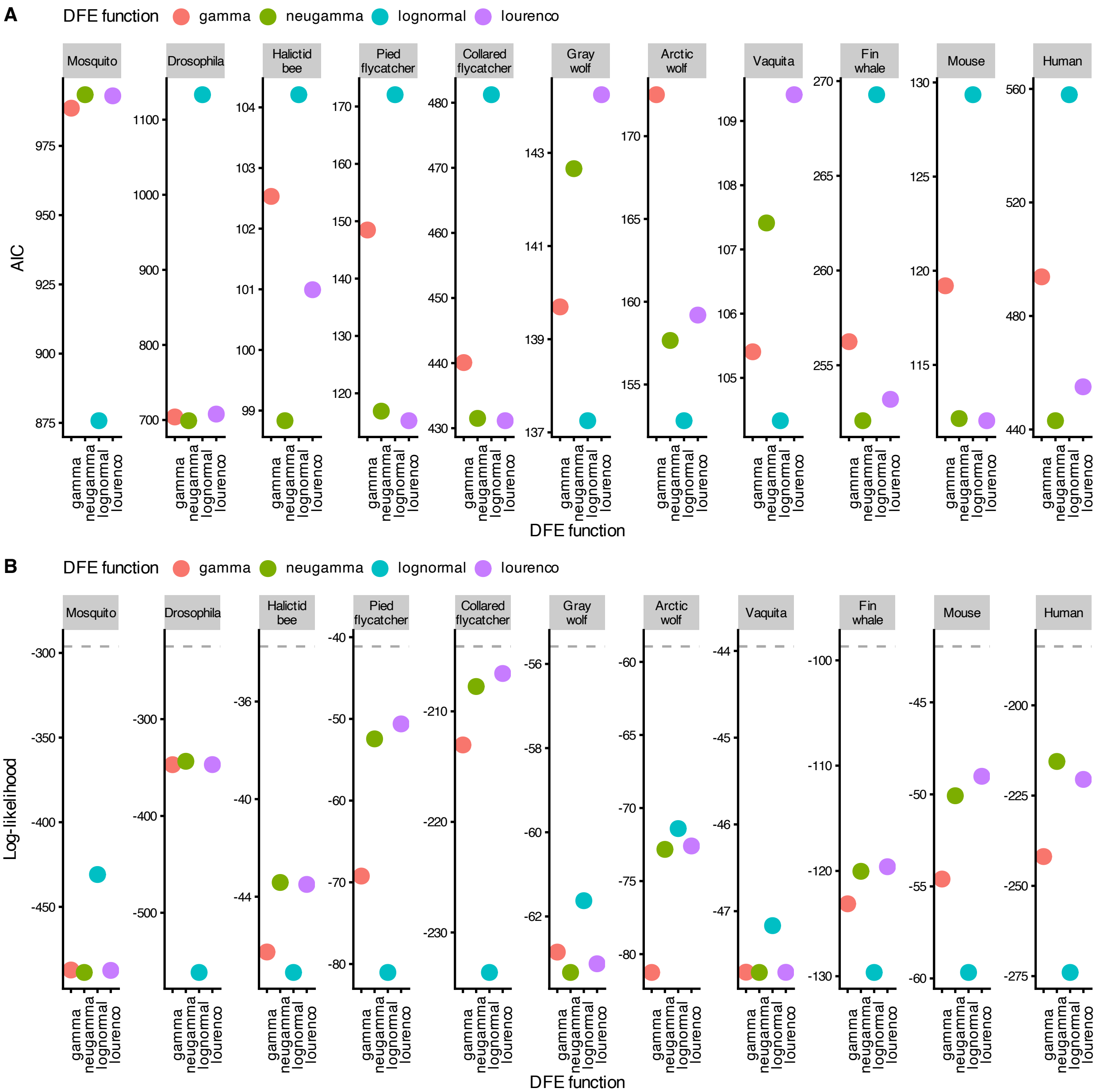

Figure AJ: Comparisons of the (A) AIC, and (B) log-likelihoods of the DFE inference assuming that the DFE follows a gamma, neugamma, lognormal, or FGM-derived (lourenco) distribution. The gray dashed line in (B) represents the maximum log-likelihood derived from the data compared to itself.

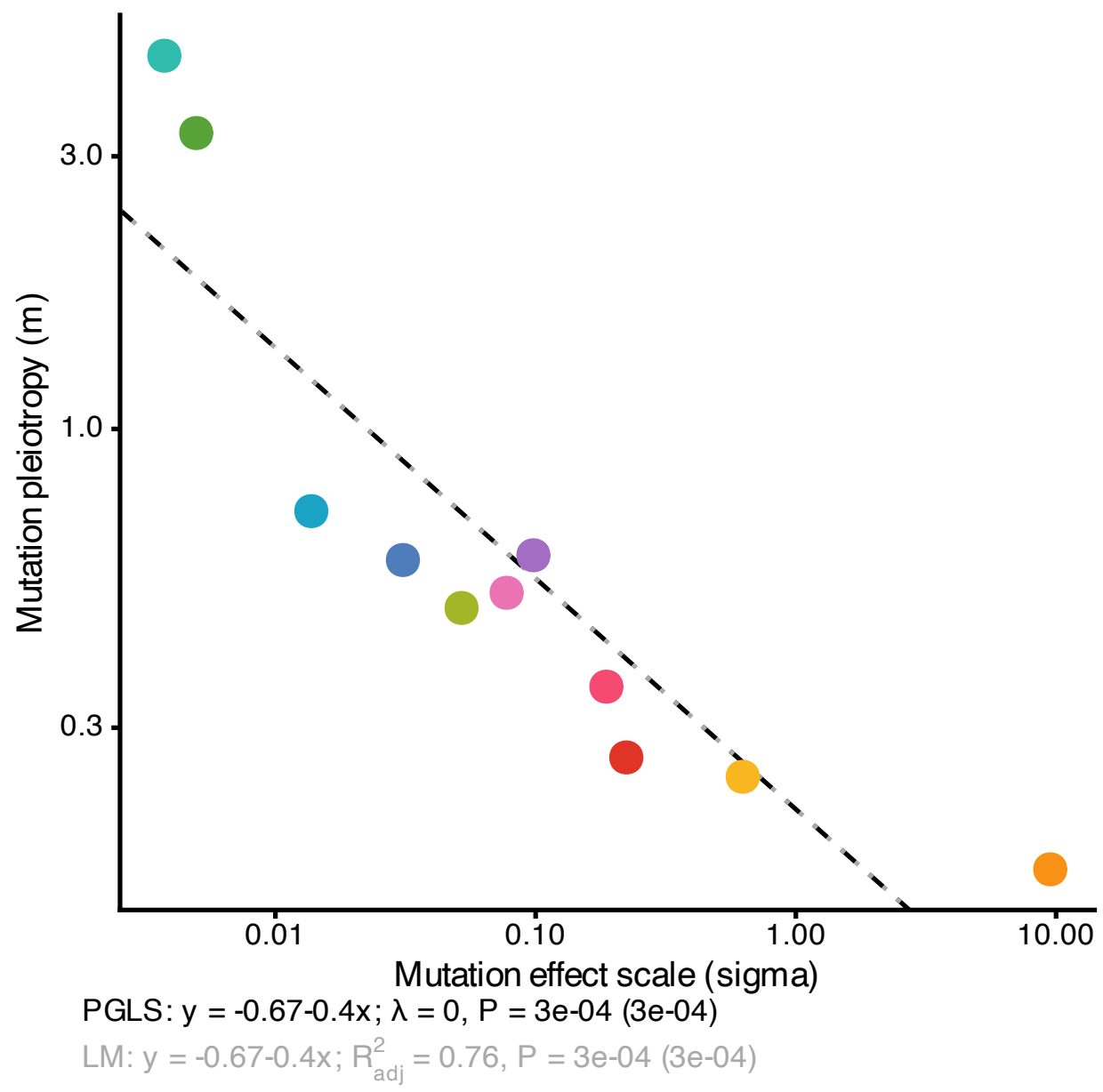

Figure AK: The inferred mutation pleiotropy (m) and mutation effect scale (σ) parameters in the FGM-derived DFE are negatively correlated. Both axes are on a log10 scale. Points are colored by datasets.

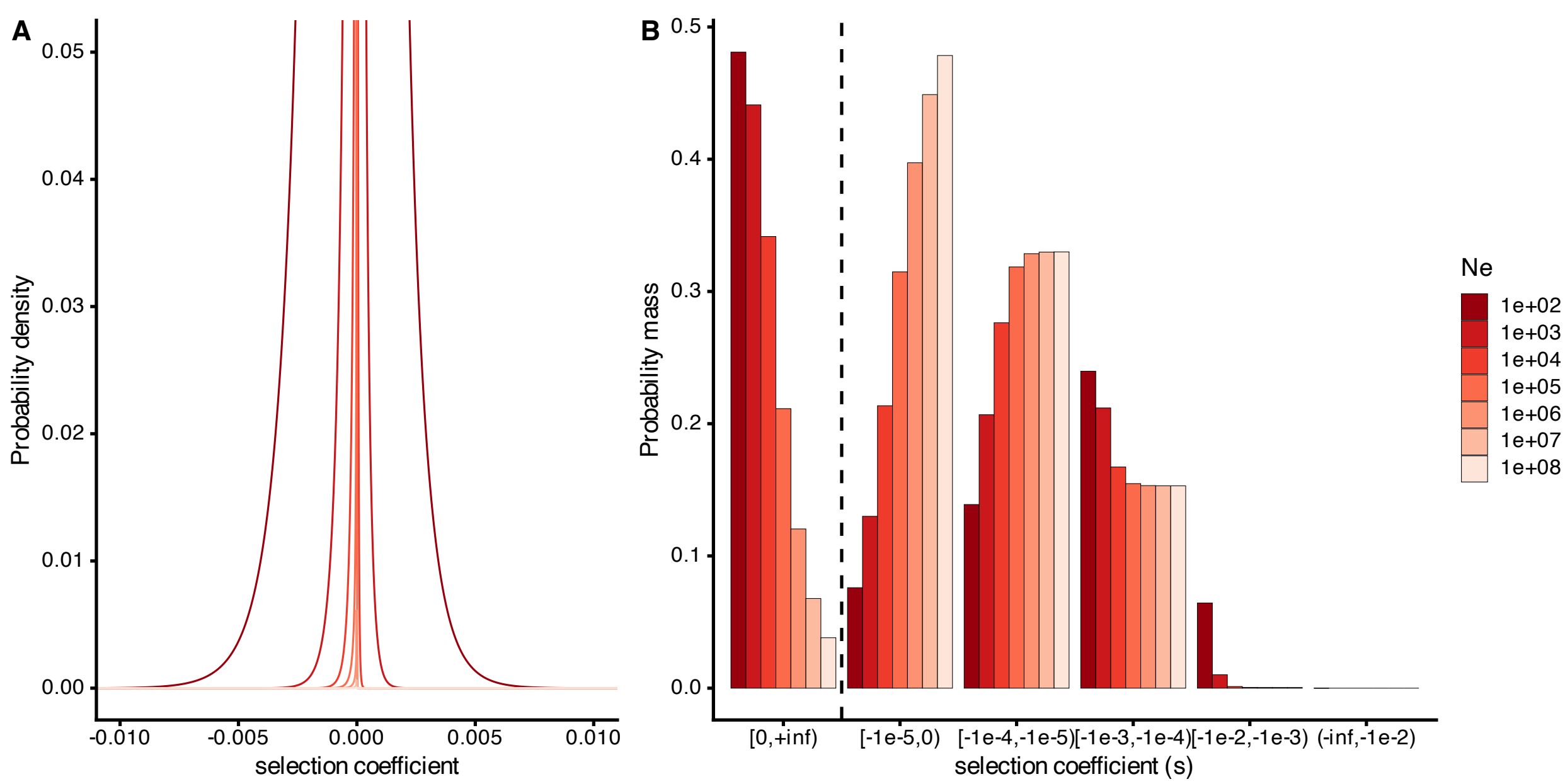

Figure AL: The FGM-derived DFE at mutation-selection-drift equilibrium becomes more dispersed as the long-term population size (N_a) declines. (A) The probability density function near $-0.01 < s < 0.01$ for N_a ranging from 100 to 10^8 (red to light-red). The mutation pleiotropy (m) is set at 0.5 and the mutation effect scale (σ) is set at 0.01. (B) The estimated proportion of each class of mutations with N_a ranging from 100 to 10^8. From left to right, mutations range from beneficial ($s > 0$), (nearly) neutral ($-10^{-5} < s \leq 0$) to strongly deleterious ($s \leq -0.01$). Dashed line marks $s = 0$. As N_a increases from 100 to 10^8, the proportion of beneficial mutation decreases while the proportion of (nearly) neutral mutations increases.

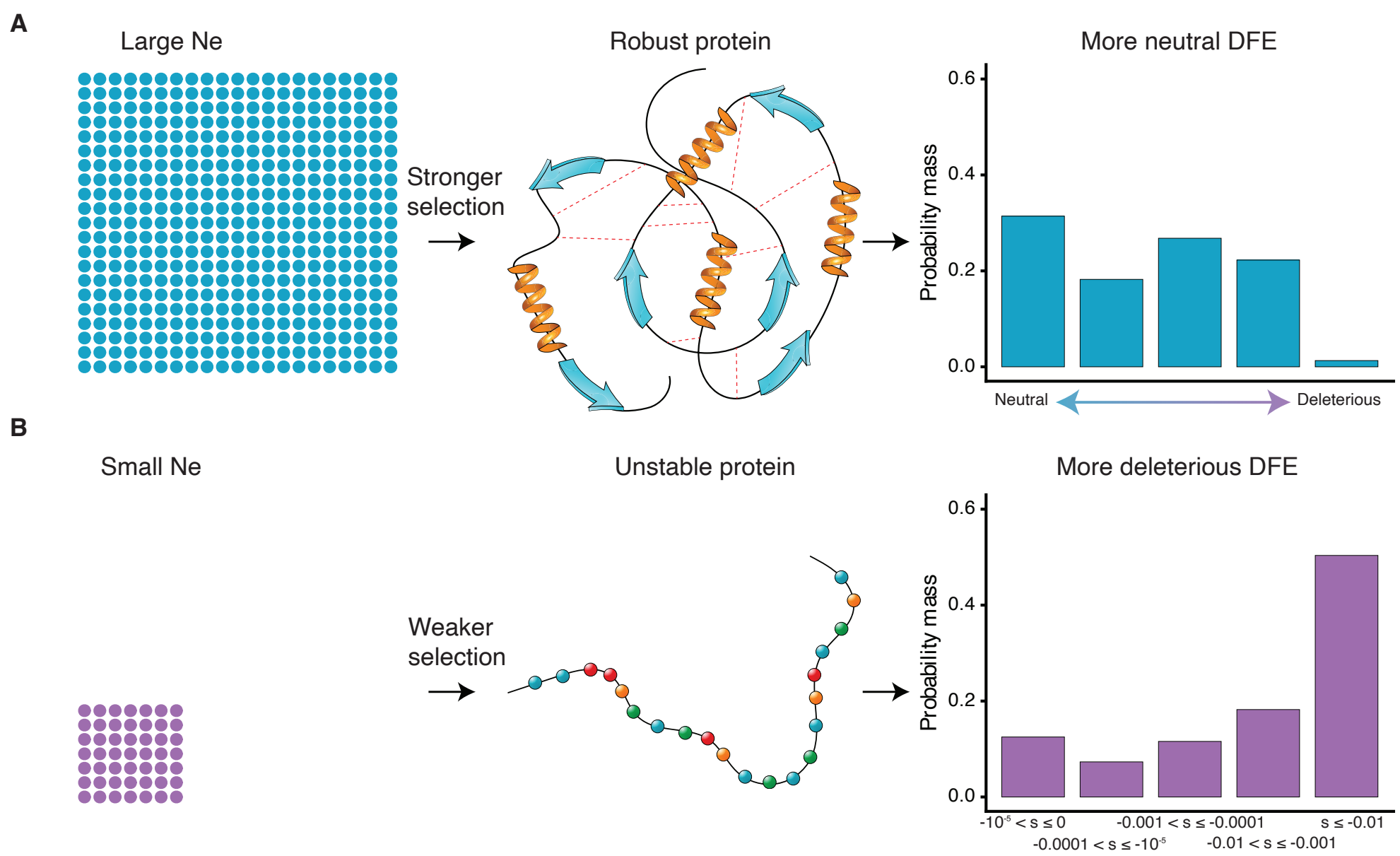

Figure AM: The drift-barrier of DFE evolution. (A) In large populations, efficient natural selection results in robust proteins. Because proteins are robust, subsequent deleterious mutations, whose effects we measured in our study, are only weakly deleterious. The inferred DFE becomes more neutral. (B) In smaller populations, natural selection is less effective at optimizing proteins. Thus, subsequent deleterious mutations, whose effects we measured in our study, are more strongly deleterious. The inferred DFE becomes more deleterious. Proportions of mutations in various categories of $|s|$ are generated assuming a gamma-distributed DFE, with the same population-scaled shape and scale parameters of $\alpha = 0.2, \beta = 5000$, but different N_a of 10^6 in (A) and 10000 in (B).

References

[1] Geraldine A. Van der Auwera et al. "From FastQ Data to High-Confidence Variant Calls: The Genome Analysis Toolkit Best Practices Pipeline". In: *Current Protocols in Bioinformatics* 43.1 (2013), pp. 11.10.1–11.10.33. DOI: 10.1002/0471250953.bi1110s43.

[2] Pablo Cingolani et al. "A Program for Annotating and Predicting the Effects of Single Nucleotide Polymorphisms, SnpEff". In: *Fly* 6.2 (2012), pp. 80–92. DOI: 10.4161/fly.19695.

[3] Christian D. Huber et al. "Determining the Factors Driving Selective Effects of New Nonsynonymous Mutations". In: *Proceedings of the National Academy of Sciences* 114.17 (2017), pp. 4465–4470. DOI: 10.1073/pnas.1619508114.

[4] Jacqueline A. Robinson et al. "The Critically Endangered Vaquita Is Not Doomed to Extinction by Inbreeding Depression". In: *Science* 376.6593 (2022), pp. 635–639. DOI: 10.1126/science.abm1742.

[5] Ying Zhen et al. "Greater Strength of Selection and Higher Proportion of Beneficial Amino Acid Changing Mutations in Humans Compared with Mice and Drosophila Melanogaster". In: *Genome Research* 31.1 (2021), pp. 110–120. DOI: 10.1101/gr.256636.119.

[6] The Anopheles gambiae 1000 Genomes Consortium. "Genetic Diversity of the African Malaria Vector Anopheles Gambiae". In: *Nature* 552.7683 (2017), pp. 96–100. DOI: 10.1038/nature24995.

[7] The Anopheles gambiae 1000 Genomes Consortium. "Genome Variation and Population Structure among 1142 Mosquitoes of the African Malaria Vector Species Anopheles Gambiae and Anopheles Coluzzii". In: *Genome Research* 30.10 (2020), pp. 1533–1546. DOI: 10.1101/gr.262790.120.

[8] Arlete Dina Albano Troco. "Resistência a insecticidas em Anopheles gambiae s.l. na região de Luanda, Angola". MA thesis. Instituto de Higiene e Medicina Tropical, 2012.

[9] Xiuwen Zheng et al. "A High-Performance Computing Toolset for Relatedness and Principal Component Analysis of SNP Data". In: *Bioinformatics* 28.24 (2012), pp. 3326–3328. DOI: 10.1093/bioinformatics/bts606.

[10] Sarah D. Kocher et al. "The Draft Genome of a Socially Polymorphic Halictid Bee, Lasioglossum Albipes". In: *Genome Biology* 14.12 (2013), R142. DOI: 10.1186/gb-2013-14-12-r142.

[11] AFA Smit, R Hubley, and P Green. *RepeatMasker Open-4.0*. 2013.

[12] Madeline A. Chase, Hans Ellegren, and Carina F. Mugal. "Positive Selection Plays a Major Role in Shaping Signatures of Differentiation across the Genomic Landscape of Two Independent Ficedula Flycatcher Species Pairs". In: *Evolution* 75.9 (2021), pp. 2179–2196. DOI: 10.1111/evo.14234.

[13] Madeline A. Chase, Hans Ellegren, and Carina F. Mugal. *Data from: Positive Selection Plays a Major Role in Shaping Signatures of Differentiation across the Genomic Landscape of Two Independent Ficedula Flycatcher Species Pairs*. 2021. DOI: 10.5061/DRYAD.N2Z34TMW6.

[14] Aleksandr Morgulis et al. "WindowMasker: Window-Based Masker for Sequenced Genomes". In: *Bioinformatics* 22.2 (2006), pp. 134–141. DOI: 10.1093/bioinformatics/bti774.

[15] M. Gardiner-Garden and M. Frommer. "CpG Islands in Vertebrate Genomes". In: *Journal of Molecular Biology* 196.2 (1987), pp. 261–282. DOI: 10.1016/0022-2836(87)90689-9.

[16] Krystyna Nadachowska-Brzyska et al. "PSMC Analysis of Effective Population Sizes in Molecular Ecology and Its Application to Black-and-White Ficedula Flycatchers". In: *Molecular Ecology* 25.5 (2016), pp. 1058–1072. DOI: 10.1111/mec.13540.

[17] Tanya N. Phung et al. "Complex Patterns of Sex-Biased Demography in Canines". In: *Proceedings of the Royal Society B: Biological Sciences* 286.1903 (2019), p. 20181976. DOI: 10.1098/rspb.2018.1976.

[18] Jacqueline A. Robinson et al. "Genomic Signatures of Extensive Inbreeding in Isle Royale Wolves, a Population on the Threshold of Extinction". In: *Science Advances* 5.5 (2019), eaau0757. DOI: 10.1126/sciadv.aau0757.

[19] Ani Manichaikul et al. "Robust Relationship Inference in Genome-Wide Association Studies". In: *Bioinformatics* 26.22 (2010), pp. 2867–2873. DOI: 10.1093/bioinformatics/btq559.

[20] Shaun Purcell et al. "PLINK: A Tool Set for Whole-Genome Association and Population-Based Linkage Analyses". In: *American Journal of Human Genetics* 81.3 (2007), pp. 559–575.

[21] Linnéa Smeds. *Data from: Whole-genome Analyses Provide No Evidence for Dog Introgression in Fennoscandian Wolf Populations.* 2020. DOI: 10.5061/DRYAD.8GTHT76N6.

[22] Linnéa Smeds et al. "Whole-Genome Analyses Provide No Evidence for Dog Introgression in Fennoscandian Wolf Populations". In: *Evolutionary Applications* 14.3 (2021), pp. 721–734. DOI: 10.1111/eva.13151.

[23] Sergio F. Nigenda-Morales et al. "The Genomic Footprint of Whaling and Isolation in Fin Whale Populations". In: *Nature Communications* 14.1 (2023), p. 5465. DOI: 10.1038/s41467-023-40052-z.

[24] Robert Vaser et al. "SIFT Missense Predictions for Genomes". In: *Nature Protocols* 11.1 (2016), pp. 1–9. DOI: 10.1038/nprot.2015.123.

[25] Aaron McKenna et al. "The Genome Analysis Toolkit: A MapReduce Framework for Analyzing Next-Generation DNA Sequencing Data". In: *Genome Research* 20.9 (2010), pp. 1297–1303. DOI: 10.1101/gr.107524.110.

[26] Petr Danecek et al. "Twelve Years of SAMtools and BCFtools". In: *GigaScience* 10.2 (2021), giab008. DOI: 10.1093/gigascience/giab008.

[27] Ryan N. Gutenkunst et al. "Inferring the Joint Demographic History of Multiple Populations from Multidimensional SNP Frequency Data". In: *PLOS Genetics* 5.10 (2009), e1000695. DOI: 10.1371/journal.pgen.1000695.

[28] Linnéa Smeds, Ilpo Kojola, and Hans Ellegren. "The Evolutionary History of Grey Wolf Y Chromosomes". In: *Molecular Ecology* 28.9 (2019), pp. 2173–2191. DOI: 10.1111/mec.15054.

[29] Peter D Keightley and Adam Eyre-Walker. "Joint Inference of the Distribution of Fitness Effects of Deleterious Mutations and Population Demography Based on Nucleotide Polymorphism Frequencies". In: *Genetics* 177.4 (2007), pp. 2251–2261. DOI: 10.1534/genetics.107.080663.

[30] Paula Tataru et al. "Inference of Distribution of Fitness Effects and Proportion of Adaptive Substitutions from Polymorphism Data". In: *Genetics* 207.3 (2017), pp. 1103–1119. DOI: 10.1534/genetics.117.300323.

[31] Paula Tataru and Thomas Bataillon. "polyDFEv2.0: Testing for Invariance of the Distribution of Fitness Effects within and across Species". In: *Bioinformatics* 35.16 (2019), pp. 2868–2869. DOI: 10.1093/bioinformatics/bty1060.

[32] Hadley Wickham. *ggplot2: Elegant Graphics for Data Analysis.* Springer-Verlag New York, 2016.

[33] R Core Team. *R: A Language and Environment for Statistical Computing*. R Foundation for Statistical Computing. Vienna, Austria, 2024.

[34] Lucie A. Bergeron et al. “Evolution of the Germline Mutation Rate across Vertebrates”. In: *Nature* 615.7951 (2023), pp. 285–291. DOI: 10.1038/s41586-023-05752-y.

[35] Bernard Y. Kim, Christian D. Huber, and Kirk E. Lohmueller. “Inference of the Distribution of Selection Coefficients for New Nonsynonymous Mutations Using Large Samples”. In: *Genetics* 206.1 (2017), pp. 345–361. DOI: 10.1534/genetics.116.197145.

[36] Guillaume Martin and Thomas Lenormand. “A General Multivariate Extension of Fisher’s Geometrical Model and the Distribution of Mutation Fitness Effects Across Species”. In: *Evolution* 60.5 (2006), pp. 893–907. DOI: 10.1111/j.0014-3820.2006.tb01169.x.

[37] Adam R. Boyko et al. “Assessing the Evolutionary Impact of Amino Acid Mutations in the Human Genome”. In: *PLOS Genetics* 4.5 (2008), e1000083. DOI: 10.1371/journal.pgen.1000083.

[38] Liam J. Revell. “Phytools: An R Package for Phylogenetic Comparative Biology (and Other Things)”. In: *Methods in Ecology and Evolution* 3.2 (2012), pp. 217–223. DOI: 10.1111/j.2041-210X.2011.00169.x.

[39] Liam J. Revell. “Phylogenetic Signal and Linear Regression on Species Data”. In: *Methods in Ecology and Evolution* 1.4 (2010), pp. 319–329. DOI: 10.1111/j.2041-210X.2010.00044.x.

[40] Emmanuel Paradis and Klaus Schliep. “Ape 5.0: An Environment for Modern Phylogenetics and Evolutionary Analyses in R”. In: *Bioinformatics* 35.3 (2019), pp. 526–528. DOI: 10.1093/bioinformatics/bty633.

[41] Jose Pinheiro et al. *nlme: Linear and Nonlinear Mixed Effects Models*. R package version 3.1-149. 2020.

[42] João Lourenço, Nicolas Galtier, and Sylvain Glémin. “Complexity, Pleiotropy, and the Fitness Effect of Mutations”. In: *Evolution* 65.6 (2011), pp. 1559–1571. DOI: 10.1111/j.1558-5646.2011.01237.x.

[43] Jeffrey R Adrion et al. “A community-maintained standard library of population genetic models”. In: *eLife* 9 (2020), e54967. DOI: 10.7554/eLife.54967.

[44] Hyung-Soon Yim et al. “Minke whale genome and aquatic adaptation in cetaceans”. In: *Nature Genetics* 46.1 (2014), pp. 88–92. DOI: 10.1038/ng.2835.

[45] Peter D. Keightley et al. “Analysis of the genome sequences of three *Drosophila* melanogaster spontaneous mutation accumulation lines”. In: *Genome Research* 19.7 (2009), pp. 1195–1201. DOI: 10.1101/gr.091231.109.

[46] Daniel R. Schrider et al. “Rates and Genomic Consequences of Spontaneous Mutational Events in *Drosophila* melanogaster”. In: *Genetics* 194.4 (2013), pp. 937–954. DOI: 10.1534/genetics.113.151670.

[47] Sihai Yang et al. “Parent-progeny sequencing indicates higher mutation rates in heterozygotes”. In: *Nature* 523.7561 (2015), pp. 463–467. DOI: 10.1038/nature14649.

[48] Linnéa Smeds, Anna Qvarnström, and Hans Ellegren. “Direct estimate of the rate of germline mutation in a bird”. In: *Genome Research* 26.9 (2016), pp. 1211–1218. DOI: 10.1101/gr.204669.116.

[49] Pontus Skoglund et al. “Ancient Wolf Genome Reveals an Early Divergence of Domestic Dog Ancestors and Admixture into High-Latitude Breeds”. In: *Current Biology* 25.11 (2015), pp. 1515–1519. DOI: 10.1016/j.cub.2015.04.019.

[50] Úlfur Árnason et al. “Whole-genome sequencing of the blue whale and other rorquals finds signatures for introgressive gene flow”. In: *Science Advances* 4.4 (2018), eaap9873. DOI: 10.1126/sciadv.aap9873.

[51] Sarah J. Lindsay et al. “Similarities and differences in patterns of germline mutation between mice and humans”. In: *Nature Communications* 10.1 (2019), p. 4053. DOI: 10.1038/s41467-019-12023-w.

[52] Xiaowen Tian, Brian L. Browning, and Sharon R. Browning. “Estimating the Genome-wide Mutation Rate with Three-Way Identity by Descent”. In: *The American Journal of Human Genetics* 105.5 (2019), pp. 883–893. DOI: 10.1016/j.ajhg.2019.09.012.

[53] Emma E Wade et al. “Quantifying the Fraction of New Mutations That Are Recessive Lethal”. In: *Evolution* 77.7 (2023), pp. 1539–1549. DOI: 10.1093/evolut/qpad061.

[54] Sean R. Eddy. “The C-value paradox, junk DNA and ENCODE”. In: *Current Biology* 22.21 (2012), R898–R899. DOI: 10.1016/j.cub.2012.10.002.

[55] Tabula Sapiens Consortium. “The Tabula Sapiens: A multiple-organ, single-cell transcriptomic atlas of humans”. In: *Science* 376.6594 (2022), eabl4896. DOI: 10.1126/science.abl4896.

[56] Fei Wang et al. “Cross-species comparison of amniote single-cell transcriptomes reveals evolutionary conservation and divergence in the chicken immune system”. In: *Nature Communications* 17.1 (2026), p. 6031. DOI: 10.1038/s41467-026-72642-y.

[57] Hongjie Li et al. “Fly Cell Atlas: A single-nucleus transcriptomic atlas of the adult fruit fly”. In: *Science* 375.6584 (2022), eabk2432. DOI: 10.1126/science.abk2432.

[58] David Alvarez-Ponce and Subramanian Krishnamurthy. “Organismal complexity strongly correlates with the number of protein families and domains”. In: *Proceedings of the National Academy of Sciences* 122.5 (2025), e2404332122. DOI: 10.1073/pnas.2404332122.

[59] Rebeca de la Fuente et al. “Alternative splicing across the tree of life”. In: *eLife* 13 (2025), RP94802. DOI: 10.7554/eLife.94802.

[60] Florian Bénitière, Anamaria Necsulea, and Laurent Duret. “Random genetic drift sets an upper limit on mRNA splicing accuracy in metazoans”. In: *eLife* 13 (2024), RP93629. DOI: 10.7554/eLife.93629.

[61] Katja Luck et al. “A reference map of the human binary protein interactome”. In: *Nature* 580.7803 (2020), pp. 402–408.

[62] Hong-Wen Tang et al. “Next-generation large-scale binary protein interaction network for Drosophila melanogaster”. In: *Nature Communications* 14.1 (2023), p. 2162.
